# Meta-Analytic Methodology for Basic Research: A Practical Guide

**DOI:** 10.3389/fphys.2019.00203

**Published:** 2019-03-27

**Authors:** Nicholas Mikolajewicz, Svetlana V. Komarova

**Affiliations:** ^1^Faculty of Dentistry, McGill University, Montreal, QC, Canada; ^2^Shriners Hospital for Children-Canada, Montreal, QC, Canada

**Keywords:** meta-analysis, basic research, rapid review, systematic review, MATLAB, methodology

## Abstract

Basic life science literature is rich with information, however methodically quantitative attempts to organize this information are rare. Unlike clinical research, where consolidation efforts are facilitated by systematic review and meta-analysis, the basic sciences seldom use such rigorous quantitative methods. The goal of this study is to present a brief theoretical foundation, computational resources and workflow outline along with a working example for performing systematic or rapid reviews of basic research followed by meta-analysis. Conventional meta-analytic techniques are extended to accommodate methods and practices found in basic research. Emphasis is placed on handling heterogeneity that is inherently prevalent in studies that use diverse experimental designs and models. We introduce *MetaLab*, a meta-analytic toolbox developed in MATLAB R2016b which implements the methods described in this methodology and is provided for researchers and statisticians at Git repository (https://github.com/NMikolajewicz/MetaLab). Through the course of the manuscript, a rapid review of intracellular ATP concentrations in osteoblasts is used as an example to demonstrate workflow, intermediate and final outcomes of basic research meta-analyses. In addition, the features pertaining to larger datasets are illustrated with a systematic review of mechanically-stimulated ATP release kinetics in mammalian cells. We discuss the criteria required to ensure outcome validity, as well as exploratory methods to identify influential experimental and biological factors. Thus, meta-analyses provide informed estimates for biological outcomes and the range of their variability, which are critical for the hypothesis generation and evidence-driven design of translational studies, as well as development of computational models.

## Introduction

Evidence-based medical practice aims to consolidate best research evidence with clinical and patient expertise. Systematic reviews and meta-analyses are essential tools for synthesizing evidence needed to inform clinical decision making and policy. Systematic reviews summarize available literature using specific search parameters followed by critical appraisal and logical synthesis of multiple primary studies (Gopalakrishnan and Ganeshkumar, 2013). Meta-analysis refers to the statistical analysis of the data from independent primary studies focused on the same question, which aims to generate a quantitative estimate of the studied phenomenon, for example, the effectiveness of the intervention (Gopalakrishnan and Ganeshkumar, 2013). In clinical research, systematic reviews and meta-analyses are a critical part of evidence-based medicine. However, in basic science, attempts to evaluate prior literature in such rigorous and quantitative manner are rare, and narrative reviews are prevalent. The goal of this manuscript is to provide a brief theoretical foundation, computational resources and workflow outline for performing a systematic or rapid review followed by a meta-analysis of basic research studies.

**Figure 1:**
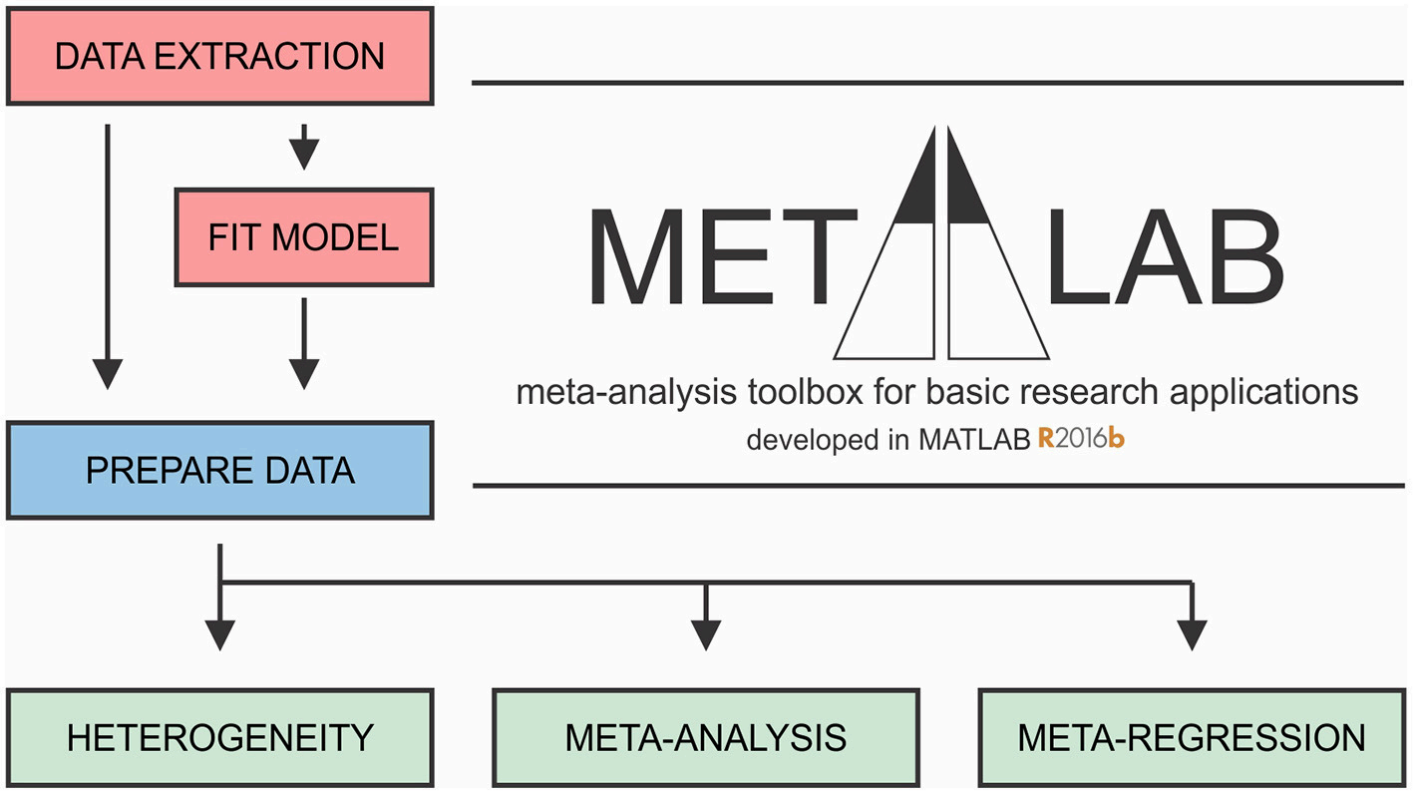
General framework of *MetaLab*. The *Data Extraction* module assists with graphical data extraction from study figures. *Fit Model* module applies Monte-Carlo error propagation approach to fit complex datasets to model of interest. Prior to further analysis, reviewers have opportunity to manually curate and consolidate data from all sources. *Prepare Data* module imports datasets from a spreadsheet into MATLAB in a standardized format. *Heterogeneity, Meta-analysis* and *Meta-regression* modules facilitate meta-analytic synthesis of data.

Meta-analyses can be a challenging undertaking, requiring tedious screening and statistical understanding. There are several guides available that outline how to undertake a meta-analysis in clinical research (Higgins and Green, 2011). Software packages supporting clinical meta-analyses include the Excel plugins MetaXL (Barendregt and Doi, 2009) and Mix 2.0 (Bax, 2016), Revman (Cochrane Collaboration, 2011), Comprehensive Meta-Analysis Software [CMA (Borenstein et al., 2005)], JASP (JASP Team, 2018) and MetaFOR library for R (Viechtbauer, 2010). While these packages can be adapted to basic science projects, difficulties may arise due to specific features of basic science studies, such as large and complex datasets and heterogeneity in experimental methodology. To address these limitations, we developed a software package aimed to facilitate meta-analyses of basic research, *MetaLab* in MATLAB R2016b, with an intuitive graphical interface that permits users with limited statistical and coding background to proceed with a meta-analytic project. We organized *MetaLab* into six modules (Figure 1), each focused on different stages of the meta-analytic process, including graphical-data extraction, model parameter estimation, quantification and exploration of heterogeneity, data-synthesis, and meta-regression.

In the present manuscript, we describe each step of the meta-analytic process with emphasis on specific considerations made when conducting a review of basic research. The complete workflow of parameter estimation using *MetaLab* is demonstrated for evaluation of intracellular ATP content in osteoblasts (OB [ATP]_ic_ dataset) based on a rapid literature review. In addition, the features pertaining to larger datasets are explored with the ATP release kinetics from mechanically-stimulated mammalian cells (ATP release dataset) obtained as a result of a systematic review in our prior work (Mikolajewicz et al., 2018).

MetaLab can be freely accessed at Git repository (https://github.com/NMikolajewicz/MetaLab), and a detailed documentation of how to use MetaLab together with a working example is available in the Supporting materials.

## Validity of Evidence in the Basic Sciences

To evaluate the translational potential of basic research, the validity of evidence must first be assessed, usually by examining the approach taken to collect and evaluate the data. Studies in the basic sciences are broadly grouped as hypothesis-generating and hypothesis-driven. The former tend to be small-sampled proof-of-principle studies and are typically exploratory and less valid than the latter. An argument can even be made that studies that report novel findings fall into this group as well, since their findings remain subject to external validation prior to being accepted by the broader scientific community. Alternatively, hypothesis-driven studies build upon what is known or strongly suggested by earlier work. These studies can also validate prior experimental findings with incremental contributions. Although such studies are often overlooked and even dismissed due to a lack of substantial novelty, their role in external validation of prior work is critical for establishing the translational potential of findings.

**Figure 2:**
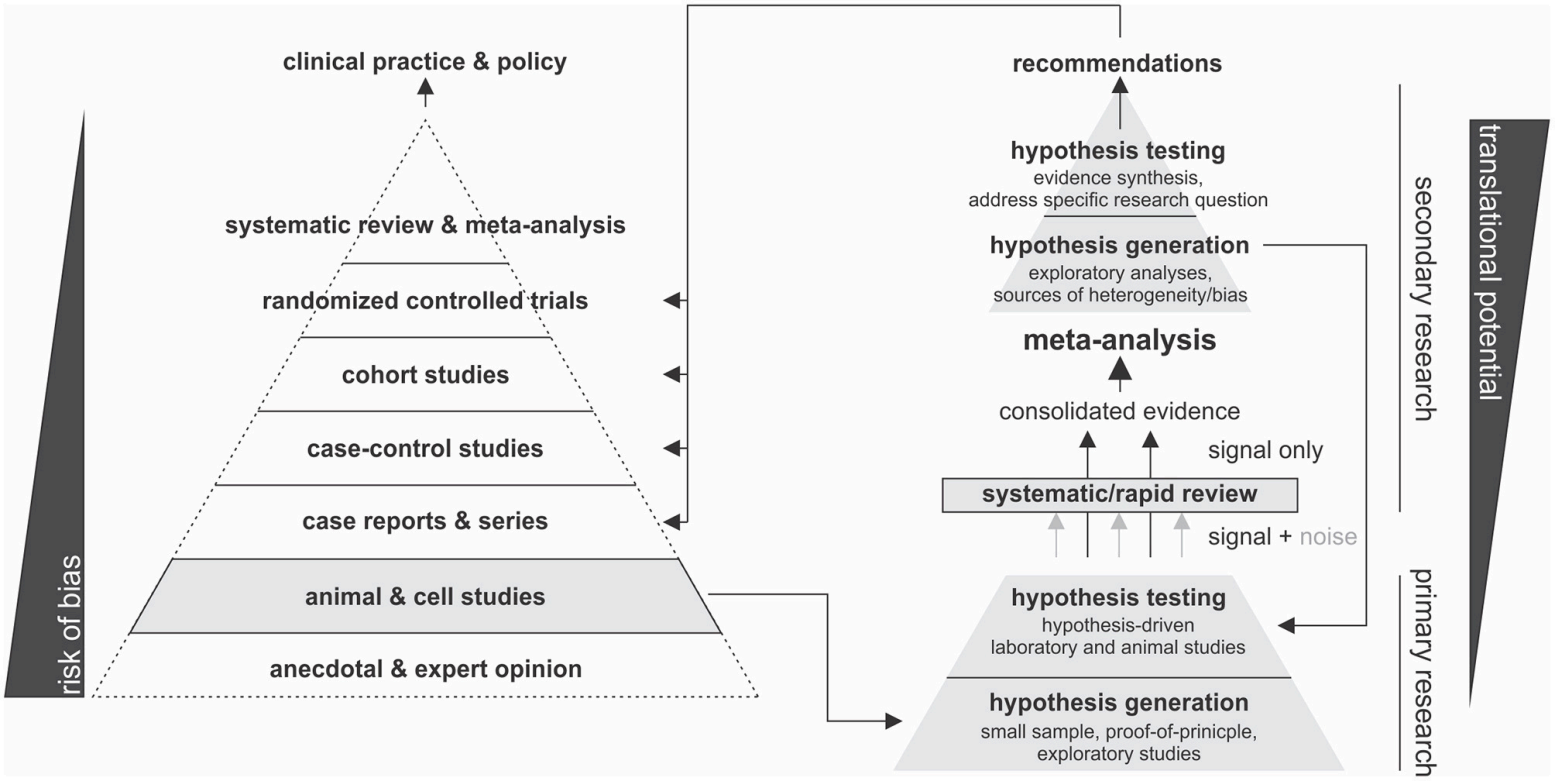
Schematic of proposed hierarchy of translational potential in basic research.

Another dimension to the validity of evidence in the basic sciences is the selection of experimental model. The human condition is near-impossible to recapitulate in a laboratory setting, therefore experimental models (e.g., cell lines, primary cells, animal models) are used to mimic the phenomenon of interest, albeit imperfectly. For these reasons, the best quality evidence comes from evaluating the performance of several independent experimental models. This is accomplished through systematic approaches that consolidate evidence from multiple studies, thereby filtering the signal from the noise and allowing for side-by-side comparison. While systematic reviews can be conducted to accomplish a qualitative comparison, meta-analytic approaches employ statistical methods which enable hypothesis generation and testing. When a meta-analysis in the basic sciences is hypothesis-driven, it can be used to evaluate the translational potential of a given outcome and provide recommendations for subsequent translational- and clinical-studies. Alternatively, if meta-analytic hypothesis testing is inconclusive, or exploratory analyses are conducted to examine sources of inconsistency between studies, novel hypotheses can be generated, and subsequently tested experimentally. Figure 2 summarizes this proposed framework.

## Steps in Quantitative Literature Review

All meta-analytic efforts prescribe to a similar workflow, outlined as follows:

1. **Formulate research question**
  - Define primary and secondary objectives
  - Determine breadth of question
2. **Identify relevant literature**
  - Construct search strategy: rapid or systematic search
  - Screen studies and determine eligibility
3. **Extract and consolidate study-level data**
  - Extract data from relevant studies
  - Collect relevant study-level characteristics and experi-mental covariates
  - Evaluate quality of studies
  - Estimate model parameters for complex relation-ships (optional)
4. **Data appraisal and preparation**
  - Compute appropriate outcome measure
  - Evaluate extent of between-study inconsistency (heterogeneity)
  - Perform relevant data transformations
  - Select meta-analytic model
5. **Synthesize study-level data into summary measure**
  - Pool data and calculate summary measure and confidence interval
6. **Exploratory analyses**
  - Explore potential sources of heterogeneity (ex. biological or experimental)
  - Subgroup and meta-regression analyses
7. **Knowledge synthesis**
  - Interpret findings
  - Provide recommendations for future work

## Meta-Analysis Methodology

### Search and Selection Strategies

The first stage of any review involves formulating a primary objective in the form of a research question or hypothesis. Reviewers must explicitly define the objective of the review before starting the project, which serves to reduce the risk of data dredging, where reviewers later assign meaning to significant findings. Secondary objectives may also be defined; however, precaution must be taken as the search strategies formulated for the primary objective may not entirely encompass the body of work required to address the secondary objective. Depending on the purpose of a review, reviewers may choose to undertake a rapid or systematic review. While the meta-analytic methodology is similar for systematic and rapid reviews, the scope of literature assessed tends to be significantly narrower for rapid reviews permitting the project to proceed faster.

#### Systematic Review and Meta-Analysis

Systematic reviews involve comprehensive search strategies that enable reviewers to identify all relevant studies on a defined topic (DeLuca et al., 2008). Meta-analytic methods then permit reviewers to quantitatively appraise and synthesize outcomes across studies to obtain information on statistical significance and relevance. Systematic reviews of basic research data have the potential of producing information-rich databases which allow extensive secondary analysis. To comprehensively examine the pool of available information, search criteria must be sensitive enough not to miss relevant studies. Key terms and concepts that are expressed as synonymous keywords and index terms, such as Medical Subject Headings (MeSH), must be combined using Boolean operators AND, OR and NOT (Ecker and Skelly, 2010). Truncations, wildcards, and proximity operators can also help refine a search strategy by including spelling variations and different wordings of the same concept (Ecker and Skelly, 2010). Search strategies can be validated using a selection of expected relevant studies. If the search strategy fails to retrieve even one of the selected studies, the search strategy requires further optimization. This process is iterated, updating the search strategy in each iterative step until the search strategy performs at a satisfactory level (Finfgeld-Connett and Johnson, 2013). A comprehensive search is expected to return a large number of studies, many of which are not relevant to the topic, commonly resulting in a specificity of <10% (McGowan and Sampson, 2005). Therefore, the initial stage of sifting through the library to select relevant studies is time-consuming (may take 6 months to 2 years) and prone to human error. At this stage, it is recommended to include at least two independent reviewers to minimize selection bias and related errors. Nevertheless, systematic reviews have a potential to provide the highest quality quantitative evidence synthesis to directly inform the experimental and computational basic, preclinical and translational studies.

#### Rapid Review and Meta-Analysis

The goal of the rapid review, as the name implies, is to decrease the time needed to synthesize information. Rapid reviews are a suitable alternative to systematic approaches if reviewers prefer to get a general idea of the state of the field without an extensive time investment. Search strategies are constructed by increasing search specificity, thus reducing the number of irrelevant studies identified by the search at the expense of search comprehensiveness (Haby et al., 2016). The strength of a rapid review is in its flexibility to adapt to the needs of the reviewer, resulting in a lack of standardized methodology (Mattivi and Buchberger, 2016). Common shortcuts made in rapid reviews are: (i) narrowing search criteria, (ii) imposing date restrictions, (iii) conducting the review with a single reviewer, (iv) omitting expert consultation (i.e., librarian for search strategy development), (v) narrowing language criteria (ex. English only), (vi) foregoing the iterative process of searching and search term selection, (vii) omitting quality checklist criteria and (viii) limiting number of databases searched (Ganann et al., 2010). These shortcuts will limit the initial pool of studies returned from the search, thus expediting the selection process, but also potentially resulting in the exclusion of relevant studies and introduction of selection bias. While there is a consensus that rapid reviews do not sacrifice quality, or synthesize misrepresentative results (Haby et al., 2016), it is recommended that critical outcomes be later verified by systematic review (Ganann et al., 2010). Nevertheless, rapid reviews are a viable alternative when parameters for computational modeling need to be estimated. While systematic and rapid reviews rely on different strategies to select the relevant studies, the statistical methods used to synthesize data from the systematic and rapid review are identical.

#### Screening and Selection

**Figure 3:**
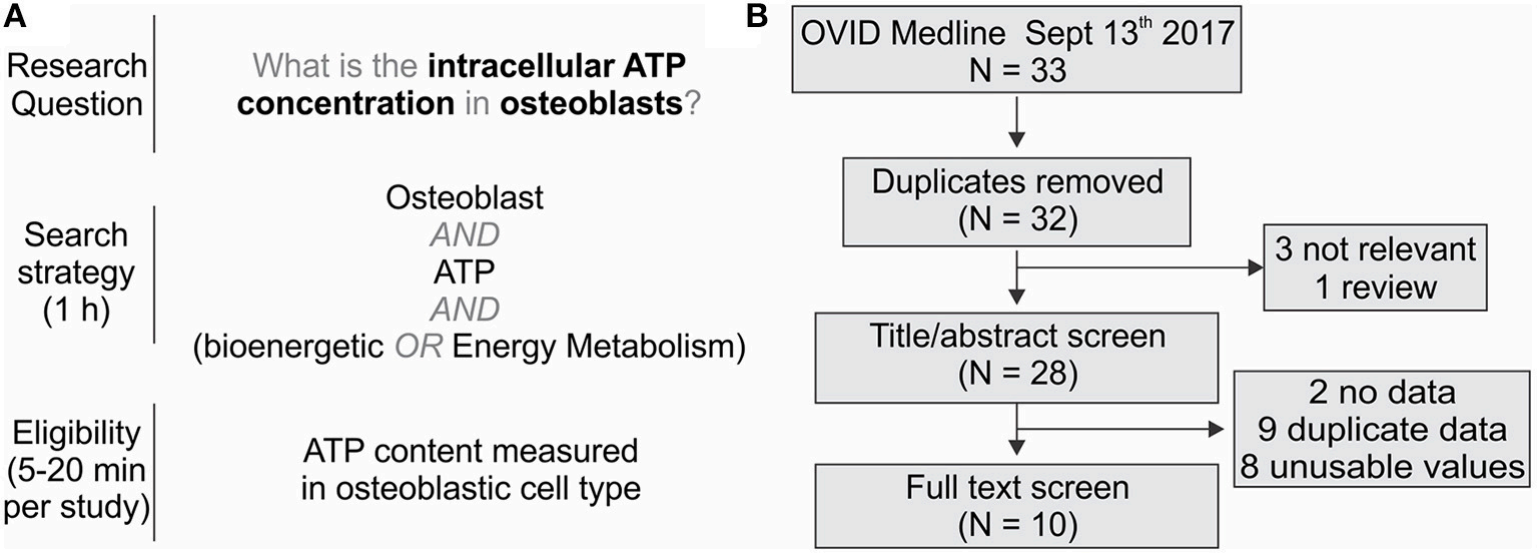
Example of the rapid review literature search. **(A)** Development of the search parameters to find literature on the intracellular ATP content in osteoblasts. **(B)** PRISMA diagram for the information flow.

When the literature search is complete (the date articles were retrieved from the databases needs to be recorded), articles are extracted and stored in a reference manager for screening. Before study screening, the inclusion and exclusion criteria must be defined to ensure consistency in study identification and retrieval, especially when multiple reviewers are involved. The critical steps in screening and selection are (1) removing duplicates, (2) screening for relevant studies by title and abstract, and (3) inspecting full texts to ensure they fulfill the eligibility criteria. There are several reference managers available including Mendeley and Rayyan, specifically developed to assist with screening systematic reviews. However, 98% of authors report using Endnote, Reference Manager or RefWorks to prepare their reviews (Lorenzetti and Ghali, 2013). Reference managers often have deduplication functions; however, these can be tedious and error-prone (Kwon et al., 2015). A protocol for faster and more reliable de-duplication in Endnote has been recently proposed (Bramer et al., 2016). The selection of articles should be sufficiently broad not to be dominated by a single lab or author. In basic research articles, it is common to find data sets that are reused by the same group in multiple studies. Therefore, additional precautions should be taken when deciding to include multiple studies published by a single group. At the end of the search, screening and selection process, the reviewer obtains a complete list of eligible full-text manuscripts. The entire screening and selection process should be reported in a PRISMA diagram, which maps the flow of information throughout the review according to prescribed guidelines published elsewhere (Moher et al., 2009). Figure 3 provides a summary of the workflow of search and selection strategies using the OB [ATP]_ic_ rapid review and meta-analysis as an example.

### Data Extraction, Initial Appraisal, and Preparation

#### Identification of Parameters to be Extracted

It is advised to predefine analytic strategies before data extraction and analysis. However, the availability of reported effect measures and study designs will often influence this decision. When reviewers aim to estimate the absolute mean difference (absolute effect), normalized mean difference, response ratio or standardized mean difference (ex. Hedges' g), they need to extract study-level means (θ_*i*_), standard deviations (*sd*(θ_*i*_)), and sample sizes (*n*_*i*_), for control (denoted $\theta_{i}^{c}$, $sd\left(\theta_{i}^{c}\right)$, and $n_{i}^{c}$) and intervention (denoted $\theta_{i}^{r}$, $sd\left(\theta_{i}^{r}\right)$, and $n_{i}^{r}$) groups, for studies *i*. To estimate absolute mean effect, only the mean ($\theta_{i}^{r}$), standard deviation $\left(sd\left(\theta_{i}^{r}\right)\right)$, and sample size ($n_{i}^{r}$) are required. In basic research, it is common for a single study to present variations of the same observation (ex. measurements of the same entity using different techniques). In such cases, each point may be treated as an individual observation, or common outcomes within a study can be pooled by taking the mean weighted by the sample size. Another consideration is inconsistency between effect size units reported on the absolute scale, for example, protein concentrations can be reported as g/cell, mol/cell, g/g wet tissue or g/g dry tissue. In such cases, conversion to a common representation is required for comparison across studies, for which appropriate experimental parameters and calibrations need to be extracted from the studies. While some parameters can be approximated by reviewers, such as cell-related parameters found in BioNumbers database (Milo et al., 2010) and equipment-related parameters presumed from manufacturer manuals, reviewers should exercise caution when making such approximations as they can introduce systematic errors that manifest throughout the analysis. When data conversion is judged to be difficult but negative/basal controls are available, scale-free measures (i.e., normalized, standardized, or ratio effects) can still be used in the meta-analysis without the need to convert effects to common units on the absolute scale. In many cases, reviewers may only be able to decide on a suitable effect size measure after data extraction is complete.

It is regrettably common to encounter unclear or incomplete reporting, especially for the sample sizes and uncertainties. Reviewers may choose to reject studies with such problems due to quality concerns or to employ conservative assumptions to estimate missing data. For example, if it is unclear if a study reports the standard deviation or standard error of the mean, it can be assumed to be a standard error, which provides a more conservative estimate. If a study does not report uncertainties but is deemed important because it focuses on a rare phenomenon, imputation methods have been proposed to estimate uncertainty terms (Chowdhry et al., 2016). If a study reports a range of sample sizes, reviewers should extract the lowest value. Strategies to handle missing data should be pre-defined and thoroughly documented.

In addition to identifying relevant primary parameters, *a priori* defined study-level characteristics that have a potential to influence the outcome, such as species, cell type, specific methodology, should be identified and collected in parallel to data extraction. This information is valuable in subsequent exploratory analyses and can provide insight into influential factors through between-study comparison.

#### Quality Assessment

Formal quality assessment allows the reviewer to appraise the quality of identified studies and to make informed and methodical decision regarding exclusion of poorly conducted studies. In general, based on initial evaluation of full texts, each study is scored to reflect the study's overall quality and scientific rigor. Several quality-related characteristics have been described (Sena et al., 2007), such as: (i) published in peer-reviewed journal, (ii) complete statistical reporting, (iii) randomization of treatment or control, (iv) blinded analysis, (v) sample size calculation prior to the experiment, (vi) investigation of a dose-response relationship, and (vii) statement of compliance with regulatory requirements. We also suggest that the reviewers of basic research studies assess (viii) objective alignment between the study in question and the meta-analytic project. This involves noting if the outcome of interest was the primary study objective or was reported as a supporting or secondary outcome, which may not receive the same experimental rigor and is subject to expectation bias (Sheldrake, 1997). Additional quality criteria specific to experimental design may be included at the discretion of the reviewer. Once study scores have been assembled, study-level aggregate quality scores are determined by summing the number of satisfied criteria, and then evaluating how outcome estimates and heterogeneity vary with study quality. Significant variation arising from poorer quality studies may justify study omission in subsequent analysis.

#### Extraction of Tabular and Graphical Data

**Figure 4:**
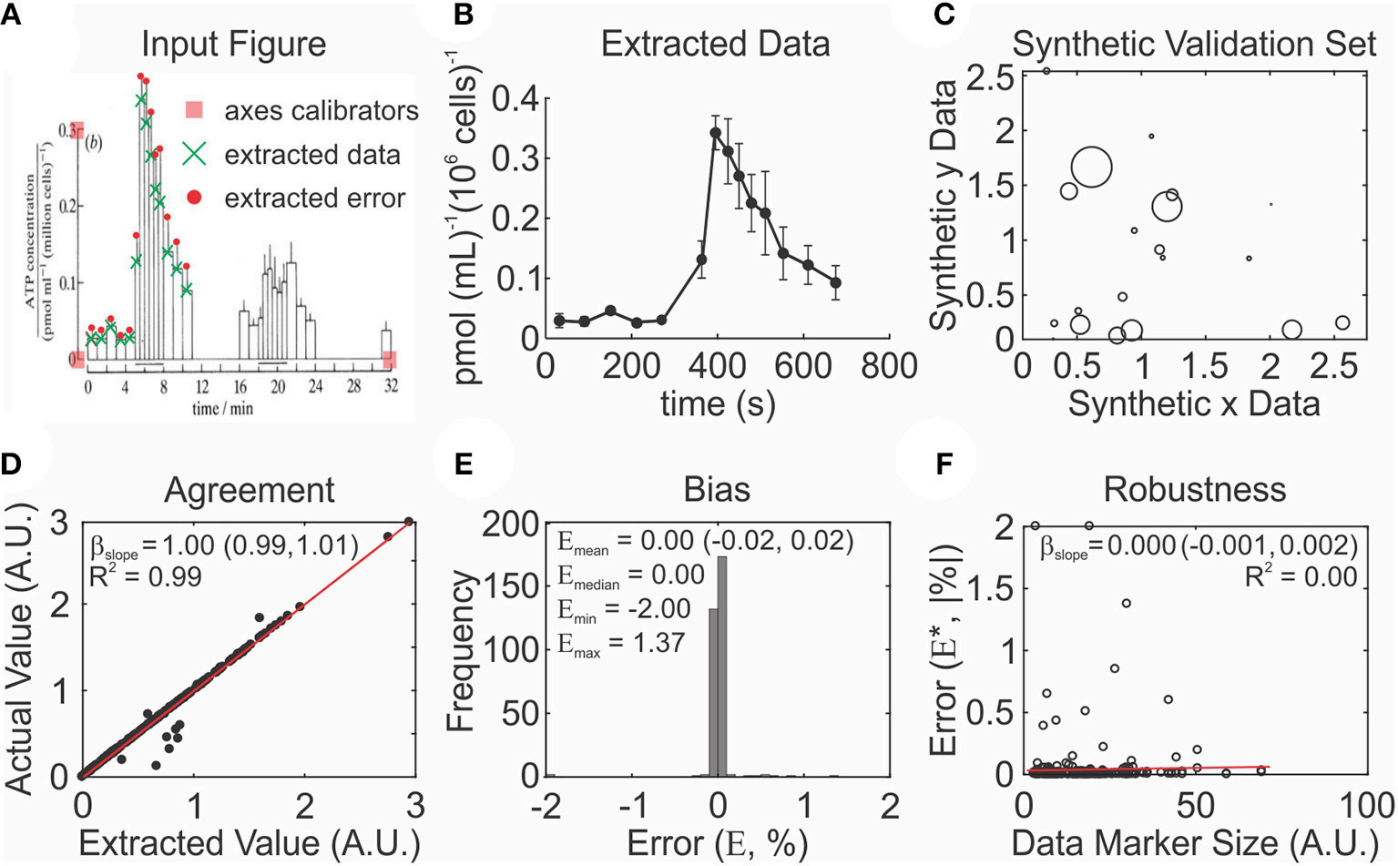
MetaLab data extraction procedure is accurate, unbiased and robust to quality of data presentation. **(A,B)** Example of graphical data extraction using MetaLab. **(A)** Original figure (Bodin et al., 1992) with axes, data points and corresponding errors marked by reviewer. **(B)** Extracted data with error terms. **(C–F)** Validation of MetaLab data-extraction module. **(C)** Synthetic datasets were constructed using randomly generated data coordinates and marker sizes. **(D)** Extracted values were consistent with true values evaluated by linear regression with the slope β_slope_, red line: line of equality. **(E)** Data extraction was unbiased, evaluated with distribution of percent errors between true and extracted values. E_mean_, E_median_, E_min_, and E_max_ are mean, median, minimum, and maximum % error respectively. **(F)** The absolute errors of extracted data were independent of data marker size, red line: line regression with the slope β_slope_.

The next step is to compile the meta-analytic data set, which reviewers will use in subsequent analysis. For each study, the complete dataset which includes parameters required to estimate the target outcome, study characteristics, as well as data necessary for unit conversion needs to be extracted. Data reporting in basic research are commonly tabular or graphical. Reviewers can accurately extract tabular data from the text or tables. However, graphical data often must be extracted from the graph directly using time consuming and error prone methods. The Data Extraction Module in *MetaLab* was developed to facilitate systematic and unbiased data extraction; Reviewers provide study figures as inputs, then specify the reference points that are used to calibrate the axes and extract the data (Figures 4A,B).

To validate the performance of the *MetaLab* Data Extraction Module, we generated figures using 319 synthetic data points plotted with varying markers sizes (Figure 4C). Extracted and actual values were correlated (*R*^2^ = 0.99) with the relationship slope estimated as 1.00 (95% CI: 0.99 to 1.01) (Figure 4D). Bias was absent, with a mean percent error of 0.00% (95% CI: −0.02 to 0.02%) (Figure 4E). The narrow range of errors between −2.00 and 1.37%, and consistency between the median and mean error indicated no skewness. Data marker size did not contribute to the extraction error, as 0.00% of the variation in absolute error was explained by marker size, and the slope of the relationship between marker size and extraction error was 0.000 (95% CI: −0.001, 0.002) (Figure 4F). There data demonstrate that graphical data can be reliably extracted using *MetaLab*.

#### Extracting Data From Complex Relationships

Basic science often focuses on natural processes and phenomena characterized by complex relationships between a series of inputs (e.g., exposures) and outputs (e.g., response). The results are commonly explained by an accepted model of the relationship, such as Michaelis-Menten model of enzyme kinetics which involves two parameters–V_max_ for the maximum rate and K_m_ for the substrate concentration half of V_max_. For meta-analysis, model parameters characterizing complex relationships are of interest as they allow direct comparison of different multi-observational datasets. However, study-level outcomes for complex relationships often (i) lack consistency in reporting, and (ii) lack estimates of uncertainties for model parameters. Therefore, reviewers wishing to perform a meta-analysis of complex relationships may need to fit study-level data to a unified model *y* = *f*(*x*, β) to estimate parameter set β characterizing the relationship (Table 1), and assess the uncertainty in β.

**Table 1:** Commonly used models of complex relationships in basic sciences.

**Model**	**Equation**	**Parameter meaning**	**Applications**
Linear model	*y* = *β*_1_*x* + *β*_2_	β_1_: slope, magnitude of relationship β_2_: intercept, response at x = 0	Reaction rates
Quadratic model (vertex form)	$y=\beta_{1}{\left(x-\beta_{2}\right)}^{2}+\beta_{3}$	β_1_: curvature factor β_2_: x at global max/min β_3_: global maxima/minimal	Trajectory modeling
Exponential model	$y=\beta_{1}e^{\beta_{2}x}$	β_1_: intercept, response at x = 0 β_2_: decay/growth constant	Population decay/growth
Michaelis-Menten, hyperbolic curve	$y=\frac{\beta_{1}x}{\beta_{2}+x}$	β_1_: max response β_2_: x at half max response	Enzyme kinetics, reaction rates, infection rates, drug clearance
Sigmoidal E_max_ Model, Hill Function	$y=\;\frac{\beta_{1}x^{\beta_{3}}}{{\left(\beta_{2}\right)}^{\beta_{3}}+x^{\beta_{3}}}$	β_1_: max response β_2_: x at half max response β_3_: slope-related term	Dose-response relationships, pharmaco dynamics

**Figure 5:**
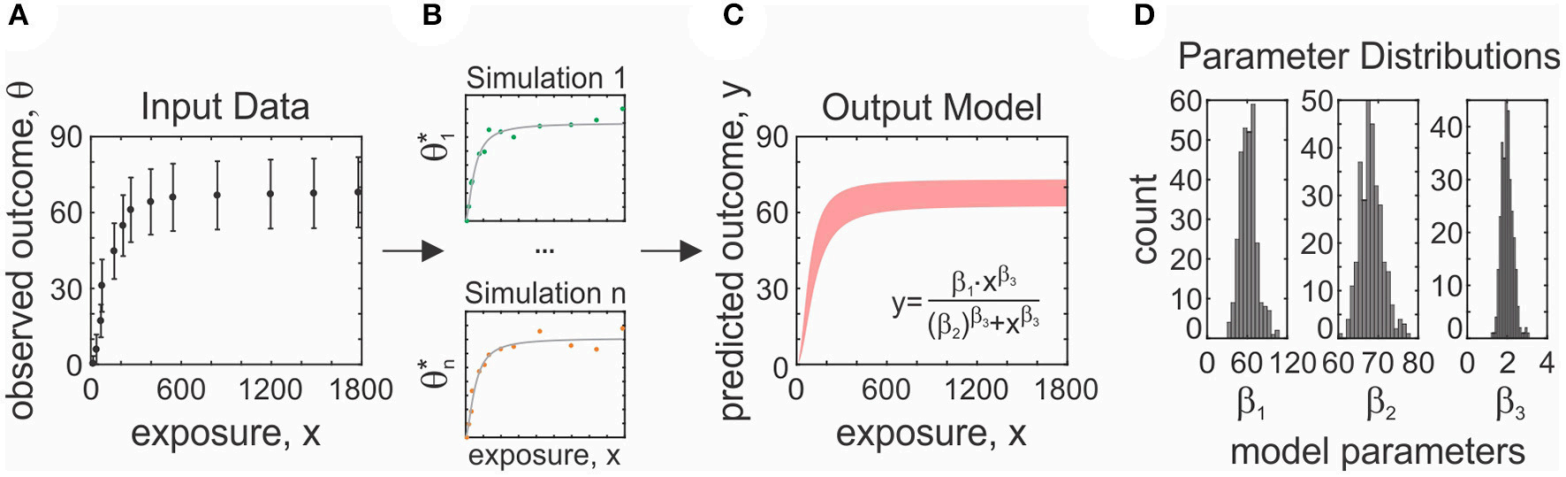
Model parameter estimation with Monte-Carlo error propagation method. **(A)** Study-level data taken from ATP release meta-analysis. **(B)** Assuming sigmoidal model, parameters were estimated using *Fit Model MetaLab* module by randomly sampling data from distributions defined by study level data. Model parameters were estimated for each set of sampled data. **(C)** Final model using parameters estimated from 400 simulations. **(D)** Distributions of parameters estimated for given dataset are unimodal and symmetrical.

The study-level data can be fitted to a model using conventional fitting methods, in which the model parameter error terms depend on the goodness of fit and number of available observations. Alternatively, a Monte Carlo simulation approach (Cox et al., 2003) allows for the propagation of study-level variances (uncertainty in the model inputs) to the uncertainty in the model parameter estimates (Figure 5). Suppose that study *i* reported a set of *k* predictor variables *x* = {*x*_*j*_|1 ≤ *j* ≤ *k*} for a set of outcomes θ = {θ_*j*_|1 ≤ *j* ≤ *k*}, and that there is a corresponding set of standard deviations *sd*(θ) = {*sd*(θ_*j*_)|1 ≤ *j* ≤ *k*} and sample sizes *n* = {*n*_*j*_|1 ≤ *j* ≤ *k*} (Figure 5A). The Monte Carlo error propagation method assumes that outcomes are normally distributed, enabling pseudo random observations to be sampled from a distribution approximated by $N\left(\theta_{j},sd{\left(\theta_{j}\right)}^{2}\right)$. The pseudo random observations are then averaged to obtain a Monte-Carlo estimate $\theta_{j}^{*}$ for each observation such that

(1) $$\begin{array}{l} \theta_{j}^{*}=\frac{1}{n_{j}}\overset{n_{j}}{\underset{m=1}{\sum }}\left(\theta_{j,m}^{*}\right) \end{array}$$

where θ(*j*, *m*)^*^ represents a pseudo-random variable sampled *n*_*j*_ times from $N\left(\theta_{j},sd{\left(\theta_{j}\right)}^{2}\right)$. The relationship between *x* and $\theta^{*}=\left\{\theta_{j}^{*}|1\leq j\leq k\right\}$ is then fitted with the model of interest using the least-squares method to obtain an estimate of model parameters β (Figure 5B). After many iterations of resampling and fitting, a distribution of parameter estimates $N\left(\bar{\beta },\;sd{\left(\bar{\beta }\right)}^{2}\right)$ is obtained, from which the parameter means $\bar{\beta }$ and variances $sd{\left(\bar{\beta }\right)}^{2}$ can be estimated (Figures 5C,D). As the number of iterations *M* tend to infinity, the parameter estimate converges to the expected value *E*(β).

(2) $$\begin{array}{l} \underset{M\to \infty }{\lim}\frac{1}{M}\left(\beta_{1}+\beta_{2}+\ldots +\beta_{M}\right)=E(\beta ) \end{array}$$

It is critical for reviewers to ensure the data is consistent with the model such that the estimated parameters sufficiently capture the information conveyed in the underlying study-level data. In general, reliable model fittings are characterized by normal parameter distributions (Figure 5D) and have a high goodness of fit as quantified by *R*^2^. The advantage of using the Monte-Carlo approach is that it works as a black box procedure that does not require complex error propagation formulas, thus allowing handling of correlated and independent parameters without additional consideration.

#### Study-Level Effect Sizes

Depending on the purpose of the review product, study-level outcomes θ_*i*_ can be expressed as one of several effect size measures. The absolute effect size, computed as a mean outcome or absolute difference from baseline, is the simplest, is independent of variance, and retains information about the context of the data (Baguley, 2009). However, the use of absolute effect size requires authors to report on a common scale or provide conversion parameters. In cases where a common scale is difficult to establish, a scale-free measure, such as standardized, normalized or relative measures can be used. Standardized mean differences, such Hedges' g or Cohen d, report the outcome as the size of the effect (difference between the means of experimental and control groups) relative to the overall variance (pooled and weighted standard deviation of combined experimental and control groups). The standardized mean difference, in addition to odds or risk ratios, is widely used in meta-analysis of clinical studies (Vesterinen et al., 2014), since it allows to summarize metrics that do not have unified meaning (e.g., a pain score), and takes into account the variability in the samples. However, the standardized measure is rarely used in basic science since study outcomes are commonly a defined measure, sample sizes are small, and variances are highly influenced by experimental and biological factors. Other measures that are more suited for basic science are the normalized mean difference, which expresses the difference between the outcome and baseline as a proportion of the baseline (alternatively called the percentage difference), and response ratio, which reports the outcome as a proportion of the baseline. All discussed measures have been included in *MetaLab* (Table 2).

**Table 2:** Types of effect sizes.

**Measure**	**Mean**	**Standard error**
Absolute	$\theta_{i}\;=\;\{\begin{array}{c} \theta_{i}^{r}-\theta_{i}^{c},if\;\theta_{i}^{c}\;reported\\ \;\theta_{i}^{r},else \end{array}$	$se\left(\theta_{i}\right)\;=\;\{\begin{array}{c} \sqrt{\frac{n_{i}^{c}+n_{i}^{r}}{n_{i}^{c}n_{i}^{r}}}sd{\left(\theta_{i}\right)}^{2},if\;\theta_{i}^{c}\;reported\\ \frac{sd\left(\theta_{i}^{r}\right)}{\sqrt{n_{i}^{r}}},else \end{array}$ Where $sd(\theta_{i})=\sqrt{\frac{\left(n_{i}^{c}-1\right)sd\left(\theta_{i}^{c}\right)+(n_{i}^{r}-1)sd\left(\theta_{i}^{r}\right)}{n_{i}^{c}+n_{i}^{r}-2}}$
Standardized (Hedges' g)	$\theta_{i}=\frac{\theta_{i}^{r}-\theta_{i}^{c}}{sd(\theta_{i})}\cdot \left(1-\frac{3}{4\left(n_{i}^{c}+n_{i}^{r}\right)-9}\right)\;$ Where $sd(\theta_{i})=\sqrt{\frac{\left(n_{i}^{c}-1\right)sd\left(\theta_{i}^{c}\right)+(n_{i}^{r}-1)sd\left(\theta_{i}^{r}\right)}{n_{i}^{c}+n_{i}^{r}-\;2}}$	$se\left(\theta_{i}\right)=\sqrt{\frac{n_{i}^{c}+n_{i}^{r}}{n_{i}^{c}n_{i}^{r}}+\frac{\theta_{i}^{2}}{2\left(\left(n_{i}^{c}+n_{i}^{r}\right)-3.94\right)}}$
Normalized	$\theta_{i}=\frac{\theta_{i}^{r}-\theta_{i}^{c}}{\theta_{i}^{c}}$	$se\left(\theta_{i}\right)=\sqrt{\frac{{\left(\frac{sd\left(\theta_{i}^{c}\right)}{\theta_{i}^{c}}\right)}^{2}}{n_{i}^{c}}+\frac{{\left(\frac{sd\left(\theta_{i}^{r}\right)}{\theta_{i}^{r}}\right)}^{2}}{n_{i}^{r}}}$
Ratio	$\theta_{i}=\frac{\theta_{i}^{r}}{\theta_{i}^{c}}$	$se\left(\theta_{i}\right)=\sqrt{\frac{{\left(\theta_{i}^{r}\right)}^{2}}{{\left(\theta_{i}^{c}\right)}^{2}}\left(\frac{sd{\left(\theta_{i}^{r}\right)}^{2}}{n_{i}^{r}{\left(\theta_{i}^{r}\right)}^{2}}+\frac{sd{\left(\theta_{i}^{c}\right)}^{2}}{n_{i}^{c}{\left(\theta_{i}^{c}\right)}^{2}}\right)}$

*Provided are formulas to calculate the mean and standard error for the specified effect sizes*.

### Data Synthesis

The goal of any meta-analysis is to provide an outcome estimate that is representative of all study-level findings. One important feature of the meta-analysis is its ability to incorporate information about the quality and reliability of the primary studies by weighing larger, better reported studies more heavily. The two quantities of interest are the overall estimate and the measure of the variability in this estimate. Study-level outcomes θ_*i*_ are synthesized as a weighted mean $\hat{\theta }$ according to the study-level weights *w*_*i*_:

(3) $$\begin{array}{l} \hat{\theta }=\frac{\sum_{i}^{N}\left(\theta_{i}\cdot w_{i}\right)}{\sum_{i}\left(w_{i}\right)} \end{array}$$

where *N* is number of studies or datasets. The choice of a weighting scheme dictates how study-level variances are pooled to estimate the variance of the weighted mean. The weighting scheme thus significantly influences the outcome of meta-analysis, and if poorly chosen, potentially risks over-weighing less precise studies and generating a less valid, non-generalizable outcome. Thus, the notion of defining an *a priori* analysis protocol has to be balanced with the need to assure that the dataset is compatible with the chosen analytic strategy, which may be uncertain prior to data extraction. We provide strategies to compute and compare different study-level and global outcomes and their variances.

#### Weighting Schemes

To generate valid estimates of cumulative knowledge, studies are weighed according to their reliability. This conceptual framework, however, deteriorates if reported measures of precision are themselves flawed. The most commonly used measure of precision is the inverse variance which is a composite measure of total variance and sample size, such that studies with larger sample sizes and lower experimental errors are more reliable and more heavily weighed. Inverse variance weighting schemes are valid when (i) sampling error is random, (ii) the reported effects are homoscedastic, i.e., have equal variance and (iii) the sample size reflects the number of independent experimental observations. When assumptions (i) or (ii) are violated, sample size weighing can be used as an alternative. Despite sample size and sample variance being such critical parameters in the estimation of the global outcome, they are often prone to deficient reporting practices.

##### Potential problems with sample variance and sample size

The standard error *se*(θ_*i*_) is required to compute inverse variance weights, however, primary literature as well as meta-analysis reviewers often confuse standard errors with standard deviations *sd*(θ_*i*_) (Altman and Bland, 2005). Additionally, many assays used in basic research often have uneven error distributions, such that the variance component arising from experimental error depends on the magnitude of the effect (Bittker and Ross, 2016). Such uneven error distributions will lead to biased weighing that does not reflect true precision in measurement. Fortunately, the standard error and standard deviation have characteristic properties that can be assessed by the reviewer to determine whether inverse variance weights are appropriate for a given dataset. The study-level standard error *se*(θ_*i*_) is a measure of precision and is estimated as the product of the sample standard deviation *sd*(θ_*i*_) and margin of error $\frac{1}{\sqrt{n_{i}}}$ for study *i*. Therefore, the standard error is expected to be approximately inversely proportionate to the root of the study-level sample size *n*_*i*_

(4) $$\begin{array}{l} se(\theta_{i})\sim \frac{1}{\sqrt{n_{i}}} \end{array}$$

**Figure 6:**
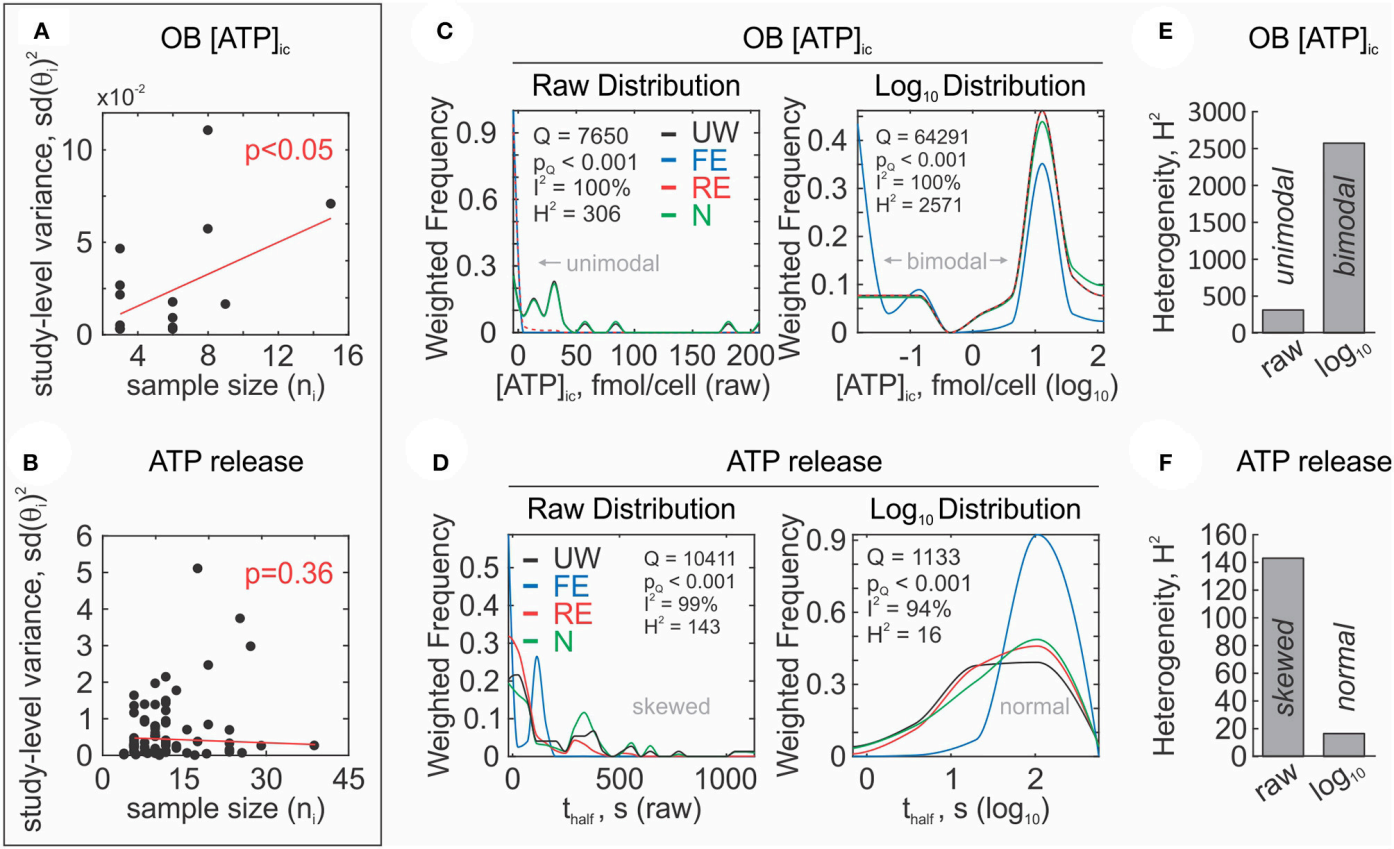
Assessment of study-level outcomes. **(A,B)** Reliability of study-level error measures. Relationship between study-level squared standard deviation $sd{\left(\theta_{i}\right)}^{2}$ and sample sizes *n*_*i*_ are assumed to be independent when reliably reported. Association between $sd{\left(\theta_{i}\right)}^{2}$ and *n*_*i*_ was present in OB [ATP]_ic_ data set **(A)** and absent in ATP release data set **(B)**, *red line*: linear regression. **(C,D)** Distributions of study-level outcomes. Assessment of unweighted (UW–*black*) and weighted (fixed effect; FE–*blue*, random effects; RE–*red*, sample-size weighting; N–*green*) study-level distributions of data from OB [ATP]_ic_ (**C**) and ATP release (**D**) data sets, before (*left*) and after log_10_ transformation (*right*). Heterogeneity was quantified by *Q, I*^2^, and *H*^2^ heterogeneity statistics. **(E,F**) After log_10_ transformation, *H*^2^ heterogeneity statistics increased for OB [ATP]_ic_ data set (**E**) and decreased for ATP release (**F**) data set.

Unlike the standard error, the standard deviation–a measure of the variance of a random variable *sd*(θ)^2^-is assumed to be independent of the sample size because it is a descriptive statistic rather than a precision statistic. Since the total observed study-level sample variance is the sum of natural variability (assumed to be constant for a phenomenon) and random error, no relationship is expected between reported standard deviations and sample sizes. These assumptions can be tested by correlation analysis and can be used to inform the reviewer about the reliability of the study-level uncertainty measures. For example, a relationship between sample size and sample variance was observed for the OB [ATP]_ic_ dataset (Figure 6A), but not for the ATP release data (Figure 6B). Therefore, in the case of the OB [ATP]_ic_ data set, lower variances are not associated with higher precision and inverse variance weighting is not appropriate. Sample sizes are also frequently misrepresented in the basic sciences, as experimental replicates and repeated experiments are often reported interchangeably (incorrectly) as sample sizes (Vaux et al., 2012). Repeated (independent) experiments refer to number of randomly sampled observations, while replicates refer to the repeated measurement of a sample from one experiment to improve measurement precision. Statistical inference theory assumes random sampling, which is satisfied by independent experiments but not by replicate measurements. Misrepresentative reporting of replicates as the sample size may artificially inflate the reliability of results. While this is difficult to identify, poor reporting may be reflected in the overall quality score of a study.

##### Inverse variance weighting

The inverse variance is the most common measure of precision, representing a composite measure of total variance and sample size. Widely used weighting schemes based on the inverse variance are fixed effect or random effects meta-analytic models. The fixed effect model assumes that all the studies sample one true effect γ. The observed outcome θ_*i*_ for study *i* is then a function of a within-study error ε_*i*_, θ_*i*_ = γ + ε_*i*_, where ε_*i*_ is normally distributed $\varepsilon_{i}\sim N\left(0,se{\left(\theta_{i}\right)}^{2}\right)$. The standard error *se*(θ_*i*_) is calculated from the sample standard deviation *sd*(θ_*i*_) and sample size *n*_*i*_ as:

(5) $$\begin{array}{l} se(\theta_{i})=\frac{sd(\theta_{i})}{\sqrt{n_{i}}} \end{array}$$

Alternatively, the random effects model supposes that each study samples a different true outcome μ_*i*_, such that the combined effect μ is the mean of a population of true effects. The observed effect θ_*i*_ for study *i* is then influenced by the intrastudy error *ε*_*i*_ and interstudy error *ξ*_*i*_, *θ*_*i*_ = *μ*_*i*_ + *ε*_*i*_ + ξ_*i*_, where ξ_*i*_ is also assumed to be normally distributed ξ_*i*_~ N(0, τ^2^), with τ^2^ representing the extent of heterogeneity, or between-study (interstudy) variance.

Study-level estimates for a fixed effect or random effects model are weighted using the inverse variance:

(6) $$\begin{array}{l} w_{i}=\{\begin{array}{cc} \frac{1}{se{\left(\theta_{i}\right)}^{2}},\; & \text{fixed effect}\\ \frac{1}{se{\left(\theta_{i}\right)}^{2}+\tau^{2}},\; & \text{random effects} \end{array} \end{array}$$

These weights are used to calculate the global outcome $\hat{\theta }$ (Equation 3) and the corresponding standard error $se\left(\hat{\theta }\right)$:

(7) $$\begin{array}{l} se\left(\hat{\theta }\right)\;=\frac{1}{\sqrt{\sum_{i}^{N}w_{i}}} \end{array}$$

where *N* = number of datasets/studies. In practice, random effects models are favored over the fixed effect model, due to the prevalence of heterogeneity in experimental methods and biological outcomes. However, when there is no between-study variability (τ^2^ = 0), the random effects model reduces to a fixed effect model. In contrast, when τ^2^ is exceedingly large and interstudy variance dominates the weighting term $\left[\tau^{2}\gg se{\left(\theta_{i}\right)}^{2}\right]$, random effects estimates will tend to an unweighted mean.

*Interstudy variance τ^2^ estimators*. Under the assumptions of a random effects model, the total variance is the sum of the intrastudy variance (experimental sampling error) and interstudy variance τ^2^ (variability of true effects). Since the distribution of true effects is unknown, we must estimate the value of τ^2^ based on study-level outcomes (Borenstein, 2009). The DerSimonian and Laird (DL) method is the most commonly used in meta-analyses (DerSimonian and Laird, 1986). Other estimators such as the Hunter and Schmidt (Hunter and Schmidt, 2004), Hedges (Hedges and Olkin, 1985), Hartung-Makambi (Hartung and Makambi, 2002), Sidik-Jonkman (Sidik and Jonkman, 2005), and Paule-Mandel (Paule and Mandel, 1982) estimators have been proposed as either alternatives or improvements over the DL estimator (Sanchez-Meca and Marin-Martinez, 2008) and have been implemented in *MetaLab* (Table 3). Negative values of τ^2^ are truncated at zero. An overview of the various τ^2^ estimators along with recommendations on their use can be found elsewhere (Veroniki et al., 2016).

**Table 3:** Interstudy variance estimators.

**Estimator**	**τ^2^ estimate**
DerSimonian-Laird (DL)*^†^	$\tau_{DL}^{2}=\frac{Q-\left(N-1\right)}{c}$
Hunter-Schmidt (HS)^*^	$\tau_{HS}^{2}=\frac{Q-N}{\sum_{i}se{\left(\theta_{i}\right)}^{-2}}$
Hedges (H)	$\tau_{H}^{2}=\frac{\sum_{i}{\left(\theta_{i}-\left(\frac{\sum_{i}\theta_{i}}{N}\right)\right)}^{2}}{N-1}-\frac{\sum_{i}se{\left(\theta_{i}\right)}^{-2}}{N}$
Hartung-Makambi (HM)*^†^	$\tau_{HM}^{2}=\frac{Q^{2}}{\left(2\left(N-1\right)+Q\right)\cdot c}$
Sidik-Jonkman (SJ)	$\tau_{SJ}^{2}=\frac{\underset{i}{\sum }\upsilon_{i}^{-1}{\left(\theta_{i}-\left(\frac{\underset{i}{\sum }\upsilon_{i}^{-1}\theta_{i}}{\underset{i}{\sum }\upsilon_{i}^{-1}}\right)\right)}^{2}}{N-\;1}$, Where $\upsilon_{i}=\left(\frac{se{\left(\theta_{i}\right)}^{2}}{\frac{\sum_{i}{\left(\theta_{i}-\bar{\theta }\right)}^{2}}{N}}+1\right)\text{ and }\bar{\theta }=\;\frac{1}{N}\sum_{i}\theta_{i}$
Paule-Mandel (PM)^#^	$\tau_{PM}^{2}=\frac{\sum_{i}w_{i}{\left(\theta_{i}-{\hat{\theta }}_{PM}\right)}^{2}-\left(\sum_{i}w_{i}^{2}se{\left(\theta_{i}\right)}^{2}-\left(\frac{\sum_{i}w_{i}^{2}se{\left(\theta_{i}\right)}^{2}}{{\sum_{i}w}_{i}}\right)\right)}{{\sum_{i}w}_{i}-\left(\frac{{\sum_{i}w}_{i}^{2}}{{\sum_{i}w}_{i}}\right)}$ Where ${\hat{\theta }}_{PM}=\frac{\underset{i}{\sum }\left(\theta_{i}\cdot w_{i}\right)}{\underset{i}{\sum }\left(w_{i}\;\right)}$

* *$Q=\sum_{i}\left(se{\left(\theta_{i}\right)}^{-2}{\left(\theta_{i}-\frac{\sum_{i}se{\left(\theta_{i}\right)}^{-2}}{\sum_{i}se{\left(\theta_{i}\right)}^{-2}\theta_{i}}\right)}^{2}\right)$. ^†^*c* = $\sum_{i}se{\left(\theta_{i}\right)}^{-2}-\frac{\sum_{i}{\left(se{\left(\theta_{i}\right)}^{-2}\right)}^{2}}{\sum_{i}se{\left(\theta_{i}\right)}^{-2}}$. ^#^iterative estimator*.

*N = number of datasets/studies*.

##### Sample-size weighting

Sample-size weighting is preferred in cases where variance estimates are unavailable or unreliable. Under this weighting scheme, study-level sample sizes are used in place of inverse variances as weights. The sampling error is then unaccounted for; however, since sampling error is random, larger sample sizes will effectively average out the error and produce more dependable results. This is contingent on reliable reporting of sample sizes which is difficult to assess and can be erroneous as detailed above. For a sample size weighted estimate, study-level sample sizes *n*_*i*_ replace weights that are used to calculate the global effect size $\hat{\theta }$, such that

(8) $$\begin{array}{l} w_{i}=n_{i} \end{array}$$

The pooled standard error $se\left(\hat{\theta }\right)$ for the global effect is then:

(9) $$\begin{array}{l} se\left(\hat{\theta }\right)\;=\sqrt{\frac{\sum_{i}^{N}\left(se{\left(\theta_{i}\right)}^{2}\cdot \left(n_{i}-1\right)\right)}{\sum_{i}^{N}\left(n_{i}-1\right)}} \end{array}$$

While sample size weighting is less affected by sampling variance, the performance of this estimator depends on the availability of studies (Marin-Martinez and Sanchez-Meca, 2010). When variances are reliably reported, sample-size weights should roughly correlate to inverse variance weights under the fixed effect model.

#### Meta-Analytic Data Distributions

One important consideration the reviewer should attend to is the normality of the study-level effects distributions assumed by most meta-analytic methods. Non-parametric methods that do not assume normality are available but are more computationally intensive and inaccessible to non-statisticians (Karabatsos et al., 2015). The performance of parametric meta-analytic methods has been shown to be robust to non-normally distributed effects (Kontopantelis and Reeves, 2012). However, this robustness is achieved by deriving artificially high estimates of heterogeneity for non-normally distributed data, resulting in conservatively wide confidence intervals and severely underpowered results (Jackson and Turner, 2017). Therefore, it is prudent to characterize the underlying distribution of study-level effects and perform transformations to normalize distributions to preserve the inferential integrity of the meta-analysis.

##### Assessing data distributions

Graphical approaches, such as the histogram, are commonly used to assess the distribution of data; however, in a meta-analysis, they can misrepresent the true distribution of effect sizes that may be different due to unequal weights assigned to each study. To address this, we can use a weighted histogram to evaluate effect size distributions (Figure 6). A weighted histogram can be constructed by first binning studies according to their effect sizes. Each bin is then assigned weighted frequencies, calculated as the sum of study-level weights within the given bin. The sum of weights in each bin are then normalized by the sum of all weights across all bins

(10) $$\begin{array}{l} P_{j}=\frac{\sum_{i}w_{ij}}{\sum_{j}^{nBins}\sum_{i}w_{ij}} \end{array}$$

where *P*_*j*_ is the weighted frequency for bin *j*, *w*_*ij*_ is the weight for the effect size in bin *j* from study *i*, and *nBins* is the total number of bins. If the distribution is found deviate from normality, the most common explanations are that (i) the distribution is skewed due to inconsistencies between studies, (ii) subpopulations exist within the dataset giving rise to multimodal distributions or (iii) the studied phenomenon is not normally distributed. The source of inconsistencies and multimodality can be explored during the analysis of heterogeneity (i.e., to determine whether study-level characteristics can explain observed discrepancies). Skewness may however be inherent to the data when values are small, variances are large, and values cannot be negative (Limpert et al., 2001) and has been credited to be characteristic of natural processes (Grönholm and Annila, 2007). For sufficiently large sample sizes the central limit theorem holds that the means of a skewed data are approximately normally distributed. However, due to common limitation in the number of studies available for meta-analyses, meta-analytic global estimates of skewed distributions are often sensitive to extreme values. In these cases, data transformation can be used to achieve a normal distribution on the logarithmic scale (i.e., lognormal distribution).

##### Lognormal distributions

Since meta-analytic methods typically assume normality, the log transformation is a useful tool used to normalize skewed distributions (Figures 6C–F). In the ATP release dataset, we found that log transformation normalized the data distribution. However, in the case of the OB [ATP]_ic_ dataset, log transformation revealed a bimodal distribution that was otherwise not obvious on the raw scale.

Data normalization by log transformation allows meta-analytic techniques to maintain their inferential properties. The outcomes synthesized on the logarithmic scale can then be transformed to the original raw scale to obtain asymmetrical confidence intervals which further accommodate the skew in the data. Study-level effect sizes θ_*i*_ can be related to the logarithmic mean Θ_*i*_ through the forward log transformation, meta-analyzed on the logarithmic scale, and back-transformed to the original scale using one of the back-transformation methods (Table 4). We have implemented three different back-transformation methods into MetaLab, including geometric approximation (anti-log), naïve approximation (rearrangement of forward-transformation method) and tailor series approximation (Higgins et al., 2008). The geometric back-transformation will yield an estimate of $\hat{\theta }$ that is approximately equal to the median of the study-level effects. The naïve or tailor series approximation differ in how the standard errors are approximated, which is used to obtain a point estimate on the original raw scale. The naïve and tailor series approximations were shown to maintain adequate inferential properties in the meta-analytic context (Higgins et al., 2008).

**Table 4:** Logarithmic Transformation Methods.

**Forward-Transformation (raw to log**_****10****_**)**		
	**Mean**	**Standard error**
	$\Theta_{i}=\log_{10}\left(\theta_{i}\right)-\left(\frac{se{\left(\Theta_{i}\right)}^{2}}{2}\right)$	$se\left(\Theta_{i}\right)=\;\sqrt{\log_{10}\left(\frac{se{\left(\theta_{i}\right)}^{2}}{{\theta_{i}}^{2}}+1\right)}$
**Back-Transformation (log**_**10**_ **to raw)**		
**Method**	**Mean**	**Standard error**
Geometric	$\hat{\theta }=10^{\hat{\Theta }}$	$\pm CI_{1-\alpha /2}\left(\hat{\theta }\right)=10^{\hat{\Theta }\pm v_{1-\alpha /2\;}\cdot se\left(\hat{\Theta }\right)}$$se\left(\hat{\theta }\right)=\frac{\left(+CI_{1-\alpha /2}\left(\hat{\theta }\right)\right)\;-\left(-CI_{1-\alpha /2}\left(\hat{\theta }\right)\right)\;}{2v_{1-\alpha /2\;}}$Where *v*_1−α/2_ corresponds to critical value
Naïve approximately	$\hat{\theta }=10^{\left(\hat{\Theta }+\frac{se{\left(\hat{\Theta }\right)}^{2}}{2}\right)}$	$se\left(\hat{\theta }\right)=\frac{1}{\sqrt{n_{i}}}\left(10^{sd{\left(\hat{\Theta }\right)}^{2})}-1\right)10^{2\hat{\Theta }+sd{\left(\hat{\Theta }\right)}^{2}}$
Tailor Series approximately	$\hat{\theta }=10^{\left(\hat{\Theta }+\frac{se{\left(\hat{\Theta }\right)}^{2}}{2}\right)}$	$se\left(\hat{\theta }\right)=\sqrt{\frac{1}{n_{i}}10^{\left(2\hat{\Theta }+{sd\left(\hat{\Theta }\right)}^{2}\right)}{sd\left(\hat{\Theta }\right)}^{2}\left(1+\left(\frac{sd{\left(\hat{\Theta }\right)}^{2}}{2}\right)\right)}$

*Forward-transformation of study-level estimates θ_i_ to corresponding log-transformed estimates Θ_i_, and back-transformation of meta-analysis outcome $\hat{\Theta }$ to the corresponding outcome $\hat{\theta }$ on the raw scale (Higgins et al., 2008). v_1−α/2_: confidence interval critical value at significance level α*.

#### Confidence Intervals

Once the meta-analysis global estimate and standard error has been computed, reviewers may proceed to construct the confidence intervals (CI). The CI represents the range of values within which the true mean outcome is contained with the probability of 1-α. In meta-analyses, the CI conveys information about the significance, magnitude and direction of an effect, and is used for inference and generalization of an outcome. Values that do not fall in the range of the CI may be interpreted as significantly different. In general, the CI is computed as the product of the standard error $se\left(\hat{\theta }\right)\;$ and the critical value *v*_1−α/2_:

(11) $$\begin{array}{l} \pm CI=\pm v_{1-\alpha /2\;}\cdot se\left(\hat{\theta }\right) \end{array}$$

##### CI estimators

The critical value *v*_1−α/2_ is derived from a theoretical distribution and represents the significance threshold for level α. A theoretical distribution describes the probability of any given possible outcome occurrence for a phenomenon. Extreme outcomes that lie furthest from the mean are known as the tails. The most commonly used theoretical distributions are the z-distribution and *t*-distribution, which are both symmetrical and bell-shaped, but differ in how far reaching or “heavy” the tails are. Heavier tails will result in larger critical values which translate to wider confidence intervals, and vice versa. Critical values drawn from a z-distribution, known as z-scores (*z*), are used when data are normal, and a sufficiently large number of studies are available (>30). The tails of a z-distribution are independent of the sample size and reflect those expected for a normal distribution. Critical values drawn from a t-distribution, known as t-scores (t), also assume data are normally-distributed, however, are used when there are fewer available studies (<30) because the t-distribution tails are heavier. This produces more conservative (wider) CIs, which help ensure that the data are not misleading or misrepresentative when there is limited evidence available. The heaviness of the t-distribution tails is dictated by the degree of freedom *df*, which is related to the number of available studies *N* (*df* = *N*−*1*) such that fewer studies will result in heavier t-distribution tails and therefore larger critical values. Importantly, the t-distribution is asymptotically normal and will thus converge to a z-distribution for a sufficiently large number of studies, resulting in similar critical values. For example, for a significance level α = 0.05 (5% false positive rate), the z-distribution will always yield a critical value *v* = 1.96, regardless of how many studies are available. The t-distribution will however yield *v* = 2.78 for 5 studies, *v* = 2.26 for 10 studies, *v* = 2.05 for 30 studies and *v* = 1.98 for 100 studies, gradually converging to 1.96 as the number of studies increases. We have implemented the z-distribution and t-distribution CI estimators into MetaLab.

#### Evaluating Meta-Analysis Performance

In general, 95% of study-level outcomes are expected to fall within the range of the 95% global CI. To determine whether the global 95% CI is consistent with the underlying study-level outcomes, the coverage of the CI can be computed as the proportion of study-level 95% CIs that overlap with the global 95% CI:

(12) $$\begin{array}{l} |\hat{\theta }-\theta_{i}|\leq v_{1-\frac{\alpha }{2}}\cdot se\left(\hat{\theta }\right)+v_{1-\frac{\alpha }{2}}\cdot se\left(\theta_{i}\right),\text{ covered}\\ |\hat{\theta }-\theta_{i}|>v_{1-\alpha /2\;}\cdot se\left(\hat{\theta }\right)+v_{1-\frac{\alpha }{2}}\cdot se\left(\theta_{i}\right),\text{not covered} \end{array}$$

The coverage is a performance measure used to determine whether inference made on the study-level is consistent with inference made on the meta-analytic level. Coverage that is less than expected for a specified significance level (i.e., <95% coverage for α = 0.05) may be indicative of inaccurate estimators, excessive heterogeneity or inadequate choice of meta-analytic model, while coverage exceeding 95% may indicate an inefficient estimator that results in insufficient statistical power.

**Figure 7:**
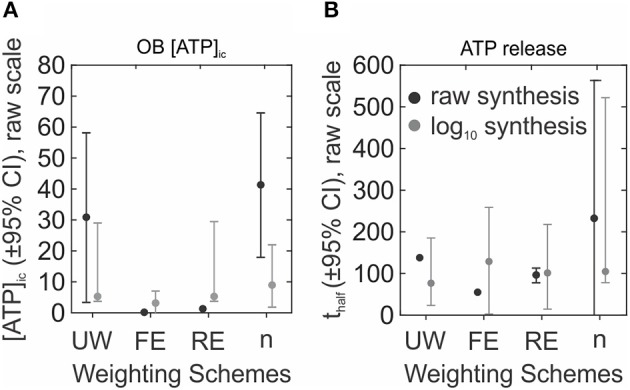
Comparison of global effect estimates using different weighting schemes. **(A,B)** Global effect estimates for OB [ATP]_ic_ **(A)** and ATP release **(B)** following synthesis of original data (raw, *black*) or of log_10_-transformed data followed by back-transformation to original scale (log_10_, *gray*). Global effects ± 95% CI were obtained with unweighted data (UW), or using fixed effect (FE), random effects (RE), and sample-size (*n*) weighting schemes.

Overall, the performance of a meta-analysis is heavily influenced by the choice of weighting scheme and data transformation (Figure 7). This is especially evident in the smaller datasets, such as our OB [ATP]_i_ example, where both the global estimates and the confidence intervals are dramatically different under different weighting schemes (Figure 7A). Working with larger datasets, such as ATP release kinetics, allows to somewhat reduce the influence of the assumed model (Figure 7B). However, normalizing data distribution (by log transformation) produces much more consistent outcomes under different weighting schemes for both datasets, regardless of the number of available studies (Figures 7A,B, *log*_10_ *synthesis*).

### Analysis of Heterogeneity

Heterogeneity refers to inconsistency between studies. A large part of conducting a meta-analysis involves quantifying and accounting for sources of heterogeneity that may compromise the validity of meta-analysis. Basic research meta-analytic datasets are expected to be heterogeneous because (*i*) basic research literature searches tend to retrieve more studies than clinical literature searches and (*ii*) experimental methodologies used in basic research are more diverse and less standardized compared to clinical research. The presence of heterogeneity may limit the generalizability of an outcome due to the lack of study-level consensus. Nonetheless, exploration of heterogeneity sources can be insightful for the field in general, as it can identify biological or methodological factors that influence the outcome.

#### Quantifying of Heterogeneity

Higgins and Thompson emphasized that a heterogeneity metric should be (i) dependent on magnitude of heterogeneity, (ii) independent of measurement scale, (iii) independent of sample size and (iv) easily interpretable (Higgins and Thompson, 2002). Regrettably, the most commonly used test of heterogeneity is the Cochrane's *Q* test (Borenstein, 2009), which has been repeatedly shown to have undesirable statistical properties (Higgins et al., 2003). Nonetheless, we will introduce it here, not because of its widespread use, but because it is an intermediary statistic used to obtain more useful measures of heterogeneity, *H*^2^ and *I*^2^. The measure of total variation *Q*_*total*_ statistic is calculated as the sum of the weighted squared differences between the study-level means θ_*i*_ and the fixed effect estimate ${\hat{\theta }}_{FE}$:

(13) $$\begin{array}{l} Q_{total}=\overset{N}{\underset{i=1}{\sum }}\left(w_{i}\cdot {\left(\theta_{i}-{\hat{\theta }}_{FE}\right)}^{2}\right)\\ \text{where }{\hat{\theta }}_{FE}=\frac{\sum_{i}{se\left(\theta_{i}\right)}^{-2}\theta_{i}}{\sum_{i}{se\left(\theta_{i}\right)}^{-2}}\text{and}w_{i}={se\left(\theta_{i}\right)}^{-2} \end{array}$$

The *Q*_*total*_ statistic is compared to a chi-square (χ^2^) distribution (*df* = *N-1*) to obtain a *p*-value, which, if significant, supports the presence of heterogeneity. However, the *Q*-test has been shown to be inadequately powered when the number of studies is too low (*N* < 10) and excessively powered when study number is too high (N > 50) (Gavaghan et al., 2000; Higgins et al., 2003). Additionally, the *Q*_*total*_ statistic is not a measure of the magnitude of heterogeneity due to its inherent dependence on the number of studies. To address this limitation, *H*^2^ heterogeneity statistics was developed as the relative excess in *Q*_*total*_ over degrees of freedom *df*:

(14) $$\begin{array}{l} H^{2}=\frac{Q_{total}}{df} \end{array}$$

*H*^2^ is independent of the number of studies in the meta-analysis and is indicative of the magnitude of heterogeneity (Higgins and Thompson, 2002). For values <1, *H*^2^ is truncated at 1, therefore values of *H*^2^ can range from one to infinity, where *H*^2^ = 1 indicates homogeneity. The corresponding confidence intervals for *H*^2^ are

(15) $$\begin{array}{l} H^{2}\pm 95\text{\% CI}={\left(e^{\ln\;\left(H\right)\pm 1.96\cdot \sqrt{\frac{1}{2\left(df-1\right)}\left(1-\frac{1}{3{\left(df\right)}^{2}}\right)}}\right)}^{2} \end{array}$$

Intervals that do not overlap with 1 indicate significant heterogeneity. A more easily interpretable measure of heterogeneity is the *I*^2^ statistic, which is a transformation of *H*^2^:

(16) $$\begin{array}{l} I^{2}=\frac{H^{2}-1}{H^{2}}\cdot 100\% \end{array}$$

The corresponding 95% CI for *I*^2^ is derived from the 95% CI for *H*^2^

(17) $$\begin{array}{l} I^{2}\pm 95\%\;CI=\frac{(H^{2}\pm 95\%\;CI)-1}{\left(H^{2}\pm 95\%\;CI\right)}\cdot 100\% \end{array}$$

Values of *I*^2^ range between 0 and 100% and describe the percentage of total variation that is attributed to heterogeneity. Like *H*^2^, *I*^2^ provides a measure of the magnitude of heterogeneity. Values of *I*^2^ at 25, 50, and 75% are generally graded as low, moderate and high heterogeneity, respectively (Higgins and Thompson, 2002; Pathak et al., 2017). However, several limitations have been noted for the *I*^2^ statistic. *I*^2^ has a non-linear dependence on τ^2^, thus *I*^2^ will appear to saturate as it approaches 100% (Huedo-Medina et al., 2006). In cases of excessive heterogeneity, if heterogeneity is partially explained through subgroup analysis or meta-regression, residual unexplained heterogeneity may still be sufficient to maintain *I*^2^ near saturation. Therefore, *I*^2^ will fail to convey the decline in overall heterogeneity, while *H*^2^ statistic that has no upper limit will allow to track changes in heterogeneity more meaningfully. In addition, a small number of studies (<10) will bias *I*^2^ estimates, contributing to uncertainties inevitable associated with small meta-analyses (von Hippel, 2015). Of the three heterogeneity statistics *Q*_*total*_, *H*^2^ and *I*^2^ described, we recommend that *H*^2^ is used as it best satisfies the criteria for a heterogeneity statistic defined by Higgins and Thompson (2002).

##### Identifying bias

Bias refers to distortions in the data that may result in misleading meta-analytic outcomes. In the presence of bias, meta-analysis outcomes are often contradicted by higher quality large sample-sized studies (Egger et al., 1997), thereby compromising the validity of the meta-analytic study. Sources of observed bias include publication bias, methodological inconsistencies and quality, data irregularities due to poor quality design, inadequate analysis or fraud, and availability or selection bias (Egger et al., 1997; Ahmed et al., 2012). At the level of study identification and inclusion for meta-analysis, systematic searches are preferred over rapid review search strategies, as narrow search strategies may omit relevant studies. Withholding negative results is also a common source of publication bias, which is further exacerbated by the small-study effect (the phenomenon by which smaller studies produce results with larger effect sizes than larger studies) (Schwarzer et al., 2015). By extension, smaller studies that produce negative results are more likely to not be published compared to larger studies that produce negative results. Identifying all sources of bias is unfeasible, however, tools are available to estimate the extent of bias present.

**Figure 8:**
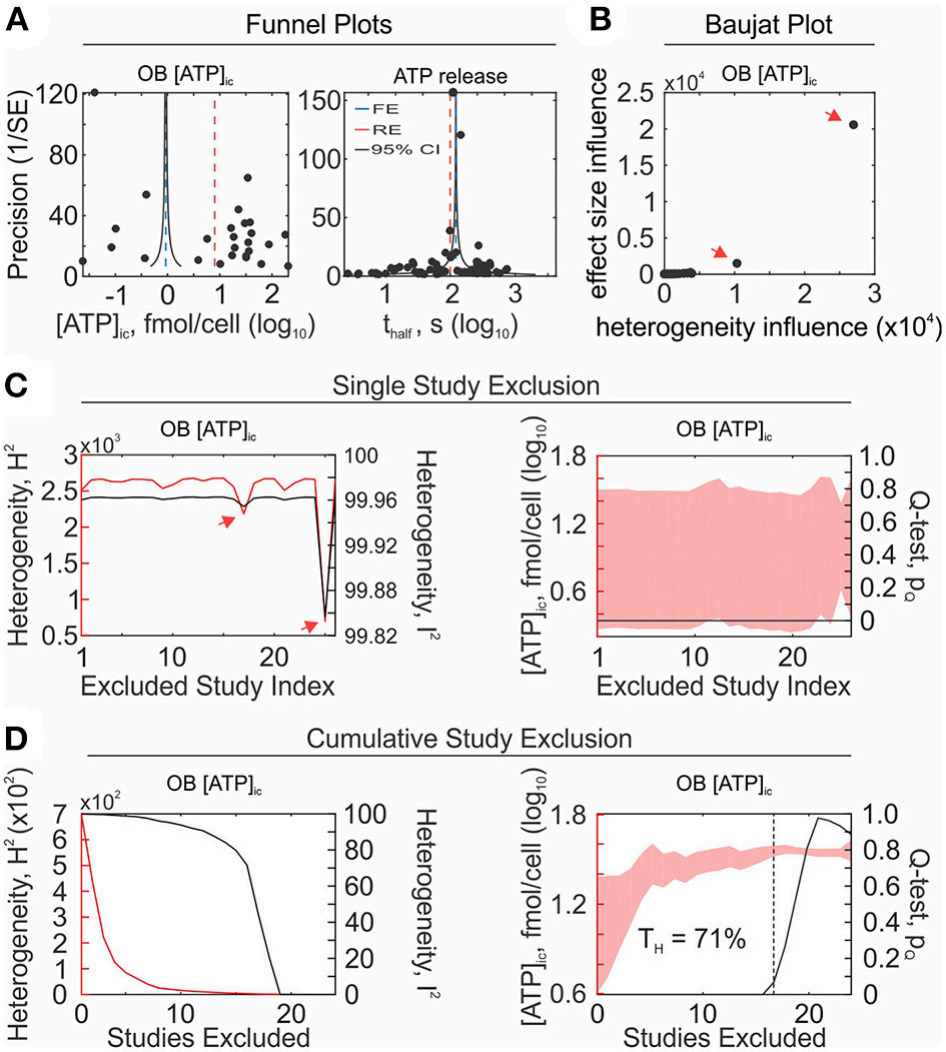
Analysis of heterogeneity and identification of influential studies. **(A)** Bias and heterogeneity in OB [ATP]_ic_ (*left*) and ATP release (*right*) data sets were assessed with funnel plots. Log_10_-transformed study-level effect sizes (black markers) were plotted in relation to their precision assessed as inverse of standard error (1/SE). *Blue dashed line*: fixed effect estimate, *red dashed line*: random effects estimate, *gray lines*: Expected 95% confidence interval (95% CI) in the absence of bias/heterogeneity. **(B)** OB [ATP]_ic_ were evaluated using Baujat plot and inconsistent and influential studies were identified in top right corner of plot (*arrows*). **(C,D)** Effect of the single study exclusion **(C)** and cumulative sequential exclusion of the most inconsistent studies **(D)**. *Left*: heterogeneity statistics, *H*^2^ (*red line*) and *I*^2^ (*black line*). *Right*: 95% CI (*red band*) and *Q*-test *p*-value (*black line*). *Arrows*: influential studies contributing to heterogeneity (same as those identified on Baujat Plot). *Dashed Black line*: homogeneity threshold T_H_ where *Q*-test *p* = 0.05.

*Funnel plots*. Funnel plots have been widely used to assess the risk of bias and examine meta-analysis validity (Light and Pillemer, 1984; Borenstein, 2009). The logic underlying the funnel plot is that in the absence of bias, studies are symmetrically distributed around the fixed effect size estimate, due to sampling error being random. Moreover, precise study-level estimates are expected to be more consistent with the global effect size than less precise studies, where precision is inversely related to the study-level standard error. Thus, for an unbiased set of studies, study-level effects θ_*i*_ plotted in relation to the inverse standard error 1/*se*(θ_*i*_) will produce a funnel shaped plot. Theoretical 95% CIs for the range of plotted standard errors are included as reference to visualize the expected distribution of studies in the absence of bias (Sterne and Harbord, 2004). When bias is present, study-level effects will be asymmetrically distributed around the global fixed-effect estimate. In the past, funnel plot asymmetries have been attributed solely to publication bias, however they should be interpreted more broadly as a general presence of bias or heterogeneity (Sterne et al., 2011). It should be noted that rapid reviews (Figure 8A, *left*) are far more subject to bias than systematic reviews (Figure 8A, *right*), due to the increased likelihood of relevant study omission.

##### Heterogeneity sensitivity analyses

Inconsistencies between studies can arise for a number of reasons, including methodological or biological heterogeneity (Patsopoulos et al., 2008). Since accounting for heterogeneity is an essential part of any meta-analysis, it is of interest to identify influential studies that may contribute to the observed heterogeneity.

*Baujat plot*. The Baujat Plot was proposed as a diagnostic tool to identify the studies that contribute most to heterogeneity and influence the global outcome (Baujat, 2002). The graph illustrates the contribution $Q_{i}^{\text{in}f}$ of each study to heterogeneity on the x-axis

(18) $$\begin{array}{l} Q_{i}^{\text{in}f}=\frac{\theta_{\text{i}}-{\hat{\theta }}_{FE}}{se{\left(\theta_{i}\right)}^{2}} \end{array}$$

and contribution $\theta_{\text{i}}^{\text{in}f}$ to global effect on the y-axis

(19) $$\begin{array}{l} \theta_{i}^{\text{in}f}=\frac{{\hat{\theta }}_{-i}-{\hat{\theta }}_{FE}}{se{\left({\hat{\theta }}_{-i}\right)}^{2}} \end{array}$$

Studies that strongly influence the global outcome and contribute to heterogeneity are visualized in the upper right corner of the plot (Figure 8B). This approach has been used to identify outlying studies in the past (Anzures-Cabrera and Higgins, 2010).

*Single-study exclusion sensitivity*. Single-study exclusion analysis assesses the sensitivity of the global outcome and heterogeneity to exclusion of single studies. The global outcomes and heterogeneity statistics are computed for a dataset with a single omitted study; single study exclusion is iterated for all studies; and influential outlying studies are identified by observing substantial declines in observed heterogeneity, as determined by *Q*_*total*_, *H*^2^, or *I*^2^, and by significant differences in the global outcome (Figure 8C). Influential studies should not be blindly discarded, but rather carefully examined to determine the reason for inconsistency. If a cause for heterogeneity can be identified, such as experimental design flaw, it is appropriate to omit the study from the analysis. All reasons for omission must be justified and made transparent by reviewers.

*Cumulative-study exclusion sensitivity*. Cumulative study exclusion sequentially removes studies to maximize the decrease in total variance *Q*_*total*_, such that a more homogenous set of studies with updated heterogeneity statistics is achieved with each iteration of exclusion (Figure 8D).

(20) $$\begin{array}{l} \;{\hat{\theta }}_{-j}\pm 95\%\;CI_{-j}\\ \text{where}j=\arg\underset{i}{\max}{\left(Q-Q_{-i}\right)}^{2} \end{array}$$

This method was proposed by Patsopoulos et al. to achieve desired levels of homogeneity (Patsopoulos et al., 2008), however, Higgins argued that its application should remain limited to (i) quantifying the extent to which heterogeneity permeates the set of studies and (ii) identifying sources of heterogeneity (Higgins, 2008). We propose the homogeneity threshold T_H_ as a measure of heterogeneity that can be derived from cumulative-study exclusion sensitivity analysis. The homogeneity threshold describes the percentage of studies that need to be removed (by the maximal Q-reduction criteria) before a homogenous set of studies is achieved. For example, in the OB [ATP]_ic_ dataset, the homogeneity threshold was 71%, since removal of 71% of the most inconsistent studies resulted in a homogeneous dataset (Figure 8D, *right*). After homogeneity is attained by cumulative exclusion, the global effect generally stabilizes with respect to subsequent study removal. This metric provides information about the extent of inconsistency present in the set of studies that is scale invariant (independent of the number of studies), and is easily interpretable.

#### Exploratory Analyses

The purpose of an exploratory analysis is to understand the data in ways that may not be represented by a pooled global estimate. This involves identifying sources of observed heterogeneity related to biological and experimental factors. Subgroup and meta-regression analyses are techniques used to explore known data groupings define by study-level characteristics (i.e., covariates). Additionally, we introduce the cluster-covariate dependence analysis, which is an unsupervised exploratory technique used to identify covariates that coincide well will natural groupings within the data, and the intrastudy regression analysis, which is used to validate meta-regression outcomes.

##### Cluster-covariate dependence analysis

**Figure 9:**
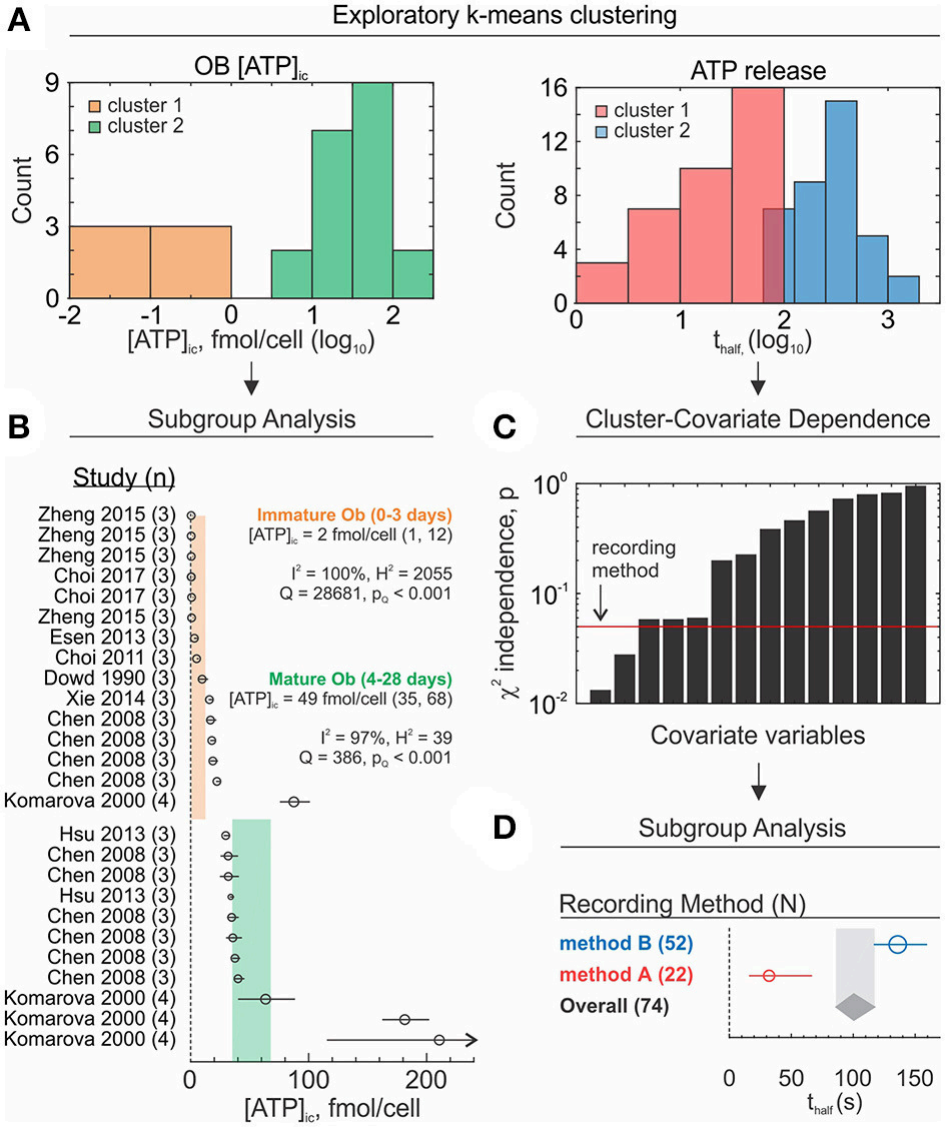
Exploratory subgroup analysis. **(A)** Exploratory k-means clustering was used to partition OB [ATP]_ic_ (*left*) and ATP release (*right*) data into potential clusters/subpopulations of interest. **(B)** Subgroup analysis of OB [ATP]_ic_ data by differentiation status (immature – 0 to 3 day osteoblasts vs. mature – 4 to 28 day osteoblasts). Subgroup outcomes (fmol ATP/cell) estimated using sample-size weighting-scheme; *black markers*: Study-level outcomes ± 95% CI, marker sizes are proportional to sample size *n*. *Orange and green bands*: 95% CI for immature and mature osteoblast subgroups, respectively. **(C)** Dependence between ATP release cluster membership and known covariates/characteristics was assessed using Pearson's χ^2^ independence test. *Black bars*: χ^2^ test *p*-values for each covariate-cluster dependence test. *Red line*: α = 0.05 significance threshold. *Arrow*: most influential covariate (ex. recording method). **(D)** Subgroup analysis of ATP release by recording method. Subgroup outcomes (t_half_) estimated using random effects weighting, τ^2^ computed using DerSimonian-Laird estimator. *Round markers*: subgroup estimates ± 95% CI, marker sizes are proportional to number of studies per subgroup *N*. *Gray band/diamond*: global effect ± 95% CI.

Natural groupings within the data can be informative and serve as a basis to guide further analysis. Using an unsupervised k-means clustering approach (Lloyd, 1982), we can identify natural groupings within the study-level data and assign cluster memberships to these data (Figure 9A). Reviewers then have two choices: either proceed directly to subgroup analysis (Figure 9B) or look for covariates that co-cluster with cluster memberships (Figure 9C) In the latter case, dependencies between cluster memberships and known data covariates can be tested using Pearson's Chi-Squared test for independence. Covariates that coincide with clusters can be verified by subgroup analysis (Figure 9D). The dependence test is limited by the availability of studies and requires that at least 80% of covariate-cluster pairs are represented by at least 5 studies (McHugh, 2013). Clustering results should be considered exploratory and warrant further investigation due to several limitations. If the subpopulations were identified through clustering, however they do not depend on extracted covariates, reviewers risk assigning misrepresentative meaning to these clusters. Moreover, conventional clustering methods always converge to a result, therefore the data will still be partitioned even in the absence of natural data groupings. Future adaptations of this method might involve using different clustering algorithms (hierarchical clustering) or independence tests (G-test for independence) as well as introducing weighting terms to bias clustering to reflect study-level precisions.

##### Subgroup analysis

Subgroup analyses attempt to explain heterogeneity and explore differences in effects by partitioning studies into characteristic groups defined by study-level categorical covariates (Figures 9B,D; Table 5). Subgroup effects are estimated along with corresponding heterogeneity statistics. To evaluate the extent to which subgroup covariates contribute to observed inconsistencies, the explained heterogeneity *Q*_*between*_ and unexplained heterogeneity *Q*_*within*_ can be calculated.

(21) $$\begin{array}{l} Q_{within}=\sum_{j=1}^{S}\left(\sum_{i=1}^{N_{j}}\left(se{\left(\theta_{i}\right)}^{-2}\cdot {\left(\theta_{i}-{\hat{\theta }}_{\left(FE\right)j}\right)}^{2}\right)\;\right) \end{array}$$

where *S* is the total number of subgroups per given covariate and each subgroup *j* contains *N*_*j*_ studies. The explained heterogeneity *Q*_*between*_ is then the difference between total and subgroup heterogeneity:

(22) $$\begin{array}{l} Q_{between}=Q_{total}-Q_{within} \end{array}$$

**Table 5:** Exploratory subgroup analysis.

**Subgroup summary statistics**							
**Group (N)**				**$\hat{\theta }$±** **95% CI**	***I***^**2**^ **(%)**	***H***^**2**^	***Q***
Total (74)				101 (86, 117)	94	16	1133
Method A (22)				32 (16, 66)	94	17	358
Method B (52)				136 (117, 159)	92	13	669
**Accounting for heterogeneity with subgroup analysis**							
	***Q***	***df***	***p*****-value**	**Interpretation**			
Total	1,133	73	<0.001	Data are heterogeneous			
Method A	358	21	<0.001	Data are heterogeneous			
Method B	669	51	<0.001	Data are heterogeneous			
Between	106	1	<0.001	Subgrouping explained significant heterogeneity			
Within	1,027	72	<0.001	Significant heterogeneity remains			

*Effect and heterogeneity estimates of ATP release by recording method*.

If the *p*-value for the χ^2^ distributed statistic *Q*_*between*_ is significant, the subgrouping can be assumed to explain a significant amount of heterogeneity (Borenstein, 2009). Similarly, *Q*_*within*_ statistic can be used to test whether there is any residual heterogeneity present within the subgroups.

The $R_{explained}^{2}$ is a related statistic that can be used to describe the percent of total heterogeneity that was explained by the covariate and is estimated as

(23) $$\begin{array}{l} R_{explained}^{2}=\left(1-\frac{\tau_{within}^{2}}{\tau_{total}^{2}}\right)\cdot 100\% \end{array}$$

Where pooled heterogeneity within subgroups $\tau_{within}^{2}$ represents the remaining unexplained variation (Borenstein, 2009):

(24) $$\begin{array}{l} \;\tau_{within}^{2}=\frac{\sum_{j=1}^{s}Q_{(within)j}-\sum_{j=1}^{s}df_{j}}{\sum_{j=1}^{s}c_{j}}\\ \text{where }c_{j}=\sum_{i=1}^{N_{j}}se{\left(\theta_{i}\right)}^{-2}-\frac{\sum_{i}{\left(se{\left(\theta_{i}\right)}^{-2}\right)}^{2}}{\sum_{i}se{\left(\theta_{i}\right)}^{-2}} \end{array}$$

Subgroup analysis of the ATP release dataset revealed that recording method had a major influence on ATP release outcome, such that method A produced significantly lower outcomes than method B (Figure 9D; Table 5, significance determined by non-overlapping 95% CIs). Additionally, recording method accounted for a significant amount of heterogeneity (*Q*_*between*_, *p* < 0.001), however it represented only 4% ($R_{explained}^{2}$) of the total observed heterogeneity. Needless to say, the remaining 96% of heterogeneity is significant (*Q*_*within*_, *p* < 0.001). To explore the remaining heterogeneity, additional subgroup analysis can be conducted by further stratifying method A and method B subgroups by other covariates. However, in many meta-analyses multi-level data stratification may be unfeasible if covariates are unavailable or if the number of studies within subgroups are low.

*Multiple comparisons*. When multiple subgroups are present for a given covariate, and the reviewer wishes to investigate the statistical differences between the subgroups, the problem of multiple comparisons should be addressed. Error rates are multiplicative and increase substantially as the number of subgroup comparisons increases. The Bonferroni correction has been advocated to control for false positive findings in meta-analyses (Hedges and Olkin, 1985) which involves adjusting the significance threshold:

(25) $$\begin{array}{l} \alpha^{*}=\frac{\alpha }{m} \end{array}$$

α^*^ is the adjusted significance threshold to attain intended error rates α for *m* subgroup comparisons. Confidence intervals can then be computed using α^*^ in place of α:

(26) $$\begin{array}{l} \pm CI=\pm v_{1-\alpha^{*}/2\;}\cdot se\left(\hat{\theta }\right) \end{array}$$

##### Meta-regression

**Figure 10:**
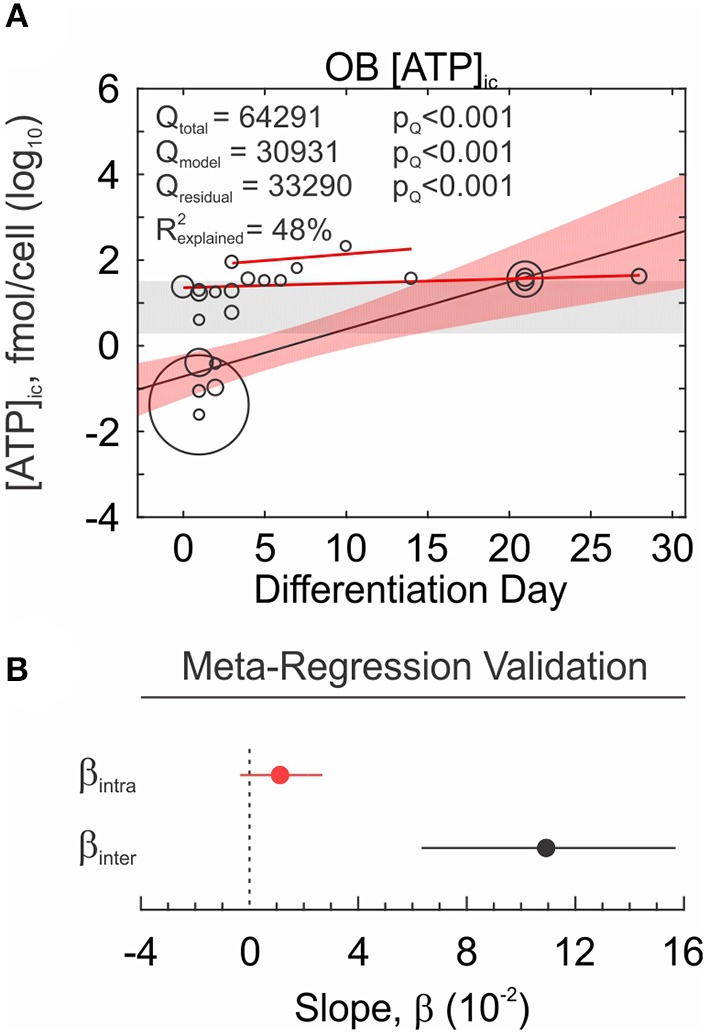
Meta-regression analysis and validation. **(A)** Relationship between osteoblast differentiation day (covariate) and intracellular ATP content (outcome) investigated by meta-regression analysis. Outcomes are on log_10_ scale, meta-regression markers sizes are proportional to weights. *Red bands*: 95% CI. *Gray bands*: 95% CI of intercept only model. *Solid red lines*: intrastudy regression. **(B)** Meta-regression coefficient β_inter_ (*black*) compared to intrastudy regression coefficient β_intra_ (*red*). Shown are regression coefficients ± 95% CI.

Meta-regression attempts to explain heterogeneity by examining the relationship between study-level outcomes and continuous covariates while incorporating the influence of categorical covariates (Figure 10A). The main differences between conventional linear regression and meta-regression are (i) the incorporation of weights and (ii) covariates are at the level of the study rather than the individual sample. The magnitude of the relationship β_*n*_ between the covariates *x*_*n,i*_ and outcome *y*_*i*_ for study *i* and covariate *n* are of interest when conducting a meta-regression analysis. It should be noted that the intercept β_0_ of a meta-regression with negligible effect of covariates is equivalent to the estimate approximated by a weighted mean (Equation 3). The generalized meta-regression model is specified as

(27) $$\begin{array}{l} y_{i}=\beta_{0}+\beta_{1}x_{1,i}+\ldots +\beta_{n}x_{n,i}+\eta_{i}+\varepsilon_{i} \end{array}$$

where intrastudy variance ε_*i*_ is

(28) $$\begin{array}{l} \varepsilon_{i}\sim N\left({0,se\left(\theta_{i}\right)}^{2}\right) \end{array}$$

and the deviation from the distribution of effects η_*i*_ depends on the chosen meta-analytic model:

(29) $$\begin{array}{l} \eta_{i}\sim \{\begin{array}{cc} 0,\; & fixed\;effect\\ N(0,\tau^{2}),\; & random\;effets \end{array} \end{array}$$

The residual Q statistic that explains the dispersion of the studies from the regression line is calculated as follows

(30) $$\begin{array}{l} Q_{residual}=\overset{N}{\underset{i=1}{\sum }}\left(w_{i}\cdot {\left(\theta_{i}-y_{i}\right)}^{2}\right)\; \end{array}$$

Where *y*_*i*_ is the predicted value at *x*_*i*_ according to the meta-regression model. *Q*_*residual*_ is analogous to *Q*_*between*_ computed during subgroup analysis and is used to test the degree of remaining unaccounted heterogeneity. *Q*_*residual*_ is also used to approximate the unexplained interstudy variance $\tau_{residual}^{2}$

(31) $$\begin{array}{l} \;\tau_{residual}^{2}=\frac{Q_{residual}-df}{c_{total}}\\ \text{where }c_{total}=\sum_{i}se{\left(\theta_{i}\right)}^{-2}-\frac{\sum_{i}{\left(se{\left(\theta_{i}\right)}^{-2}\right)}^{2}}{\sum_{i}se{\left(\theta_{i}\right)}^{-2}} \end{array}$$

Which can be used to calculate $R_{explained}^{2}$ estimated as

(32) $$\begin{array}{l} R_{explained}^{2}=\left(1-\frac{\tau_{residual}^{2}}{\tau_{total}^{2}}\right)\cdot 100\% \end{array}$$

*Q*_*model*_ quantifies the amount of heterogeneity explained by the regression model and is analogous to *Q*_*within*_ computed during subgroup analysis.

(33) $$\begin{array}{l} Q_{model}=Q_{total}-Q_{residual} \end{array}$$

*Intrastudy regression analysis* The challenge of interpreting results from a meta-regression is that relationships that exist within studies may not necessarily exist across studies, and vice versa. Such inconsistencies are known as aggregation bias and in the context of meta-analyses can arise from excess heterogeneity or from confounding factors at the level of the study. This problem has been acknowledged in clinical meta-analyses (Thompson and Higgins, 2002), however cannot be corrected without access to individual patient data. Fortunately, basic research studies often report outcomes at varying predictor levels (ex. dose-response curves), permitting for intrastudy (within-study) relationships to be evaluated by the reviewer. If study-level regression coefficients can be computed for several studies (Figure 10A, *red lines*), they can be pooled to estimate an overall effect β_*intra*_. The meta-regression interstudy coefficient β_*inter*_ and the overall intrastudy-regression coefficient β_*intra*_ can then be compared in terms of magnitude and sign. Similarity in the magnitude and sign validates the existence of the relationship and characterizes its strength, while similarity in sign but not the magnitude, still supports the presence of the relationship, but calls for additional experiments to further characterize it. For the Ob [ATP]_i_ dataset, the magnitude of the relationship between osteoblast differentiation day and intracellular ATP concentration was inconsistent between intrastudy and interstudy estimates, however the estimates were of consistent sign (Figure 10B).

##### Limitations of exploratory analyses

When performed with knowledge and care, exploratory analysis of meta-analytic data has an enormous potential for hypothesis generation, cataloging current practices and trends, and identifying gaps in the literature. Thus, we emphasize the inherent limitations of exploratory analyses:

*Data dredging*. A major pitfall in meta-analyses is data dredging (also known as p-hacking), which refers to searching for significant outcomes only to assign meaning later. While exploring the dataset for potential patterns can identify outcomes of interest, reviewers must be wary of random patterns that can arise in any dataset. Therefore, if a relationship is observed it should be used to generate hypotheses, which can then be tested on new datasets. Steps to avoid data dredging involve defining an *a priori* analysis plan for study-level covariates, limiting exploratory analysis of rapid review meta-analyses and correcting for multiple comparisons.

*Statistical power*. The statistical power reflects the probability of rejecting the null hypothesis when the alternative is true. Meta-analyses are believed to have higher statistical power than the underlying primary studies, however this is not always true (Hedges and Pigott, 2001; Jackson and Turner, 2017). Random effects meta-analyses handle data heterogeneity by accounting for between-study variance, however this weakens the inference properties of the model. To maintain statistical powers that exceed those of the contributing studies in a random effects meta-analysis, at least five studies are required (Jackson and Turner, 2017). This consequently limits subgroup analyses that partition studies into smaller groups to isolate covariate-dependent effects. Thus, reviewers should ensure that group are not under-represented to maintain statistical power. Another determinant of statistical power is the expected effect size, which if small, will be much more difficult to support with existing evidence than if it is large. Thus, if reviewers find that there is insufficient evidence to conclude that a small effect exists, this should not be interpreted as evidence of no effect.

*Causal inference*. Meta-analyses are not a tool for establishing causal inference. However, there are several criteria for causality that can be investigated through exploratory analyses that include consistency, strength of association, dose-dependence and plausibility (Weed, 2000, 2010). For example, consistency, the strength of association, and dose-dependence can help establish that the outcome is dependent on exposure. However, reviewers are still posed with the challenge of accounting for confounding factors and bias. Therefore, while meta-analyses can explore various criteria for causality, causal claims are inappropriate, and outcomes should remain associative.

## Conclusions

Meta-analyses of basic research can offer critical insights into the current state of knowledge. In this manuscript, we have adapted meta-analytic methods to basic science applications and provided a theoretical foundation, using OB [ATP]_i_ and ATP release datasets, to illustrate the workflow. Since the generalizability of any meta-analysis relies on the transparent, unbiased and accurate methodology, the implications of deficient reporting practices and the limitations of the meta-analytic methods were discussed. Emphasis was placed on the analysis and exploration of heterogeneity. Additionally, several alternative and supporting methods have been proposed, including a method for validating meta-regression outcomes—intrastudy regression analysis, and a novel measure of heterogeneity—the homogeneity threshold. All analyses were conducted using *MetaLab*, a meta-analysis toolbox that we have developed in MATLAB R2016b. *MetaLab* has been provided for free to promote meta-analyses in basic research (https://github.com/NMikolajewicz/MetaLab).

In its current state, the translational pipeline from benchtop to bedside is an inefficient process, in one case estimated to produce ~1 clinically favorable clinical outcome for ~1,000 basic research studies (O'Collins et al., 2006). The methods we have described here serve as a general framework for comprehensive data consolidation, knowledge gap-identification, evidence-driven hypothesis generation and informed parameter estimation in computation modeling, which we hope will contribute to meta-analytic outcomes that better inform translation studies, thereby minimizing current failures in translational research.

## Author Contributions

Both authors contributed to the study conception and design, data acquisition and interpretation and drafting and critical revision of the manuscript. NM developed MetaLab. Both authors approved the final version to be published.

### Conflict of Interest Statement

The authors declare that the research was conducted in the absence of any commercial or financial relationships that could be construed as a potential conflict of interest.
