# Supplementary material for: Meta-Analytic Methodology for Basic Research: A Practical Guide
Source: Front Physiol. 2019 Mar 27;10:203. doi: 10.3389/fphys.2019.00203 (PMC6445886; doi:10.3389/fphys.2019.00203)
Supplement: Supplementary file 1 [file Data_Sheet_1.PDF]

MetaLab User Guide

Version 1.0

Nicholas Mikolajewicz, Svetlana V. Komarova

Contents

Introduction 3
Scope of Guide 3
Prerequisites 3
Usage 3
Citation 3
Contact Us 3
Overview of MetaLab Modules 4
1. Data Extraction Module 6
2. Fit Model Module 8
3. Prepare Data Module 12
4. Heterogeneity Module 15
5. Meta-Analysis Module 18
6. Meta-Regression Module 20
MATLAB Tips 23
Useful MATLAB Tips 23
Figures in MATLAB 23
MATLAB Resources 26

Introduction

MetaLab is a toolbox for conducting meta-analysis of basic research in MATLAB. It uses a graphical interface, allowing reviewers with minimal prior computation experience to conduct meta-analyses with ease. Data is imported directly from spreadsheet into MATLAB where the analysis is conducted. Conventional meta-analytic techniques have been implemented in MetaLab with extended methods intended to accommodate analysis of basic research studies.

Scope of Guide

This user guide is intended as a guide on how to use MetaLab and not as guide on how to conduct a meta-analysis. The various modules, required (and optional) inputs and generated results are explained in sufficient detail to conduct a meta-analytic project. The underlying equations and meta-analytic techniques are not included in this guide; They can be found described in detail in the corresponding manuscript.

Prerequisites

Users must have MATLAB R2016b or later installed. Spreadsheet formats supported by MetaLab are XLS, XLSX, XLSM, XLTX and XLTM files. All files provided in the MetaLab installation folder must be kept in a designated directory.

Usage

To run MetaLab, open the ‘Main.m’ file and press ‘RUN’. Users can navigate through the tool box using the provided graphical interface and user prompts.

Citation

MetaLab is provided as a free computational meta-analytic toolbox developed in MATLAB R2016b.

Contact Us

Please report any problems/bugs to Nicholas.Mikolajewicz@mail.mcgill.ca

Suggestions/improvements are also welcome.

Overview of MetaLab Modules

MetaLab is organized into six modules, each equipped to facilitate different stages of the meta-analytic process. Here we briefly outline the function of each module along with expected inputs and outputs (shown in parentheses are formats) before going into more detail in subsequent sections. The organization and flow of data in MetaLab is illustrated in **Fig. 1**.

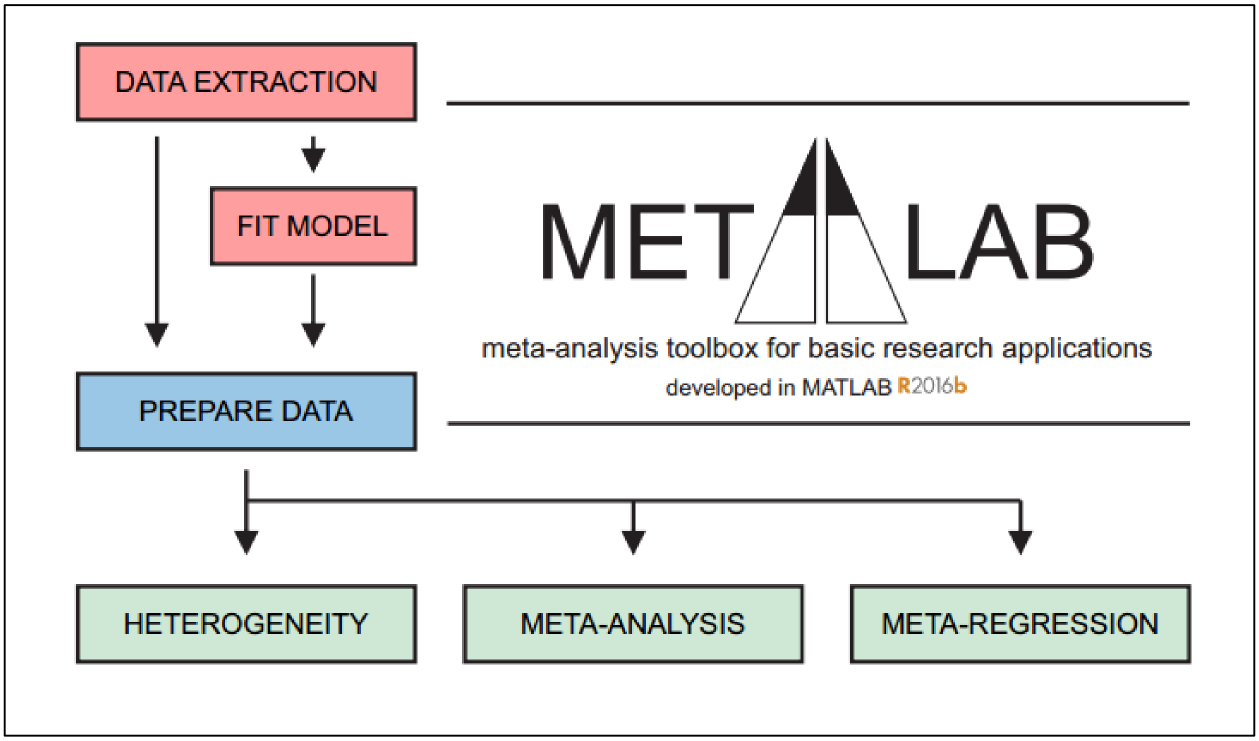

Figure 1. Metalab organization and flow of data.

1. **Data Extraction**: Facilitates systematic and unbiased data extraction. Study figures are used as inputs, axes are then calibrated at reference points specified by the reviewer, and data is extracted.
   Input: Study figures (*.PNG format*)
   Output:
   - Extracted study-level data (*MATLAB structure*, *spreadsheet*)

2. **Fit Model**: Complex relationships, including response kinetics and dose-dependencies are commonly studied in basic science. This module allows reviewers to fit extracted study-level data with linear, quadratic, exponential, hyperbolic or sigmoidal model and extract parameters of interest for subsequent meta-analysis
   Input: multi-observational data sets (*spreadsheet*)
   Output:
   - Model parameter estimates & statistics (*spreadsheet*)
   - Input data (*plot*)
   - Monte-carlo samples and model fittings (*plot*)
   - Model parameter distributiosn (*plot*)
   - Final model (*plot*)

3. **Prepare Data**: Imports prepared data from spreadsheet into MATLAB and stores data in MATLAB structure for subsequent use and compatibility with heterogeneity, meta-analysis and meta-regression modules.
    Input: meta-analytic data set (*spreadsheet*)
    Output:
    - Meta-analysis data set (*MATLAB structure*)

4. **Heterogeneity**: Evaluates heterogeneity and bias in meta-analytic data set.
    Input: meta-analysis data set (*MATLAB structure*)
    Output(s):
    - Study-level data distribution: weighted histograms (*plot*) and normal probability (*plot*)
    - Cluster-covariate dependence analysis: k-means clustered distributions (*plot*), independence test of covariate-clusters (*plot*)
    - Funnel plot (*plot*)
    - Variance vs sample size (*plot*)
    - Comparison of weighting schemes: distribution of study-level weights (*plot*), effect sizes compared under different weighting schemes (*plot*)
    - Tau-squared estimator comparison (*plot*)
    - Baujat plot (*plot*)
    - Sensitivity analyses: singly study exclusion analysis (*plot*), cumulative study exclusion analysis (*plot*)

5. **Meta-Analysis**: Synthesis of meta-analysis data set, along with subgroup analyses.
    Input: meta-analysis data set (*MATLAB structure*)
    Output(s):
    - Meta-analysis results and statistics (*spreadsheet*)
    - Subgroup analysis results and statistics (*spreadsheet*)
    - Unweighted data distributions (*plot*)
    - Study-level forest plot (*plot*)
6. **Meta-Regression**: Conducts linear meta-regression analysis with option to validate results through intrastudy (within-study) regression analysis.
    Input: meta-analysis data set (*MATLAB structure*)
    Output(s):
    - Meta-regression results and statistics (*spreadsheet*)
    - Regression diagnostic plot (*plot*)
    - Meta-regression plot (*plot*)

These features are discussed in more detail below.

1. Data Extraction Module

Overview: This module facilitates extraction of quantitative data from graphical or tabular figures. Using a ‘point and click’ interface, users calibrate graph axes and extract data from figures that are provided as input to this module. Extracted data are subsequently stored in MATLAB structure with option to export to spreadsheet.

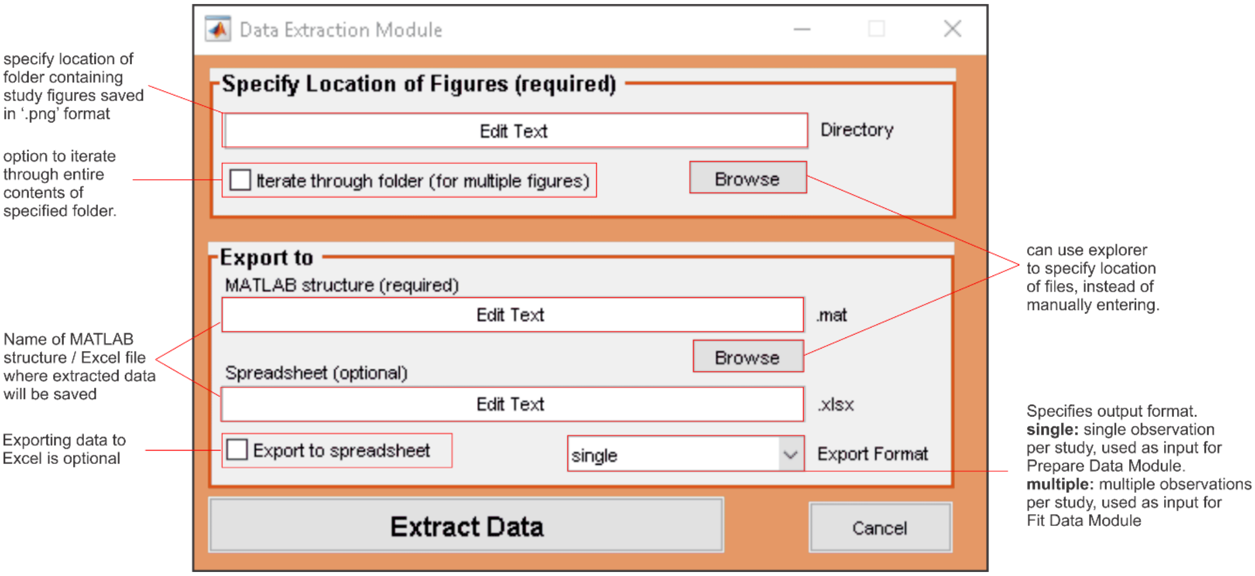

Figure 2. Data extraction module user interface

Usage

Input. To begin data extraction, users are *required* to specify the location of figures and name of MATLAB structure to which extracted data are exported (**Fig. 2**). Study figures must be saved as .PNG format in a designated folder.

> *Tip 1*: Data is extracted from one figure at a time (*default*). To iterate through all figures in saved in folder automatically, select ‘iterate’ option
>
> *Tip 2*: Save all study figures in designated directory prior to data extraction rather then adding them intermittently.
>
> *Tip 3*: Study figure inputs can be prepared by taking screenshots of graphical data with Print Screen function, pasted into image editor (ex. Microsoft Paint), cropped to size and saved as .PNG file. Saved figures should store in a folder designated for study figures. See for **Fig. 3** for example.

Name	Date	Type	Size	Tags
1_Karpatkin 1969.png	2017-02-19 3:25 PM	PNG File	109 KB	
2_Williams 1980_peakReleasevsMagitude.png	2017-02-19 4:04 PM	PNG File	43 KB	
2_Williams 1980_timelapse.png	2017-02-19 4:03 PM	PNG File	39 KB	
3_Miller 1983_peakReleasevsexposureDuration.png	2017-02-19 4:35 PM	PNG File	41 KB	
3_Miller 1983_peakReleasevsMagitude.png	2017-02-19 4:35 PM	PNG File	44 KB	
4_Milner 1990_timelapse.png	2017-02-19 4:38 PM	PNG File	59 KB	
5_Bodin 1992.png	2017-02-19 5:08 PM	PNG File	134 KB	

Figure 3. Study figures saved in designated folder. All figures are saved in .PNG format.

Tracking Extraction Progress. Once data extraction process has started, a progress tracking variable (‘currentProgress.mat’) will be created which tracks progress within the study figure directory. This ensures continuity across multiple data extraction sessions. If user is starting new set of data extraction, ensure no prior ‘currentProgress.mat’ variable exists (found in same folder as MetaLab files) otherwise the order of figures retrieved by the module will be wrong.

Axes Calibration & Data Extraction. Data Extraction Module will open study figures. Users will be directed to select figure area of interest with pointer and double click to crop and zoom in. Series of prompts will then enable user to specify key graphical features required to proceed with data extraction. Importantly, the quality of extracted data depends on how precisely the axes are calibrated and data are selected by the user. Not all inputs are required, those that are, are specified.

Extracted data is saved to MATLAB structure by default. To access extracted data, specify spreadsheet name. Export ‘single’ format assumes single observation per study and is used as input for Prepare Data Module. Export ‘multiple’ format assumes multiple observations per study and is used as input for Fit Model Module.

> *Tip 4*: If mistake is made during data extraction, users will be presented with option to redo current figure.
>
> *Tip 5*: Make sure to label figures and data during data extraction to be able to map datasets back to original studies!

2. Fit Model Module

Overview: Complex relationships are commonly studied in basic science. Fit Model Module allows reviewers to fit study-level data with linear, quadratic, exponential, hyperbolic or sigmoidal model using Monte-Carlo error propagation method. Model parameters and error terms are extracted from fitted model and can then be used for subsequent meta-analysis

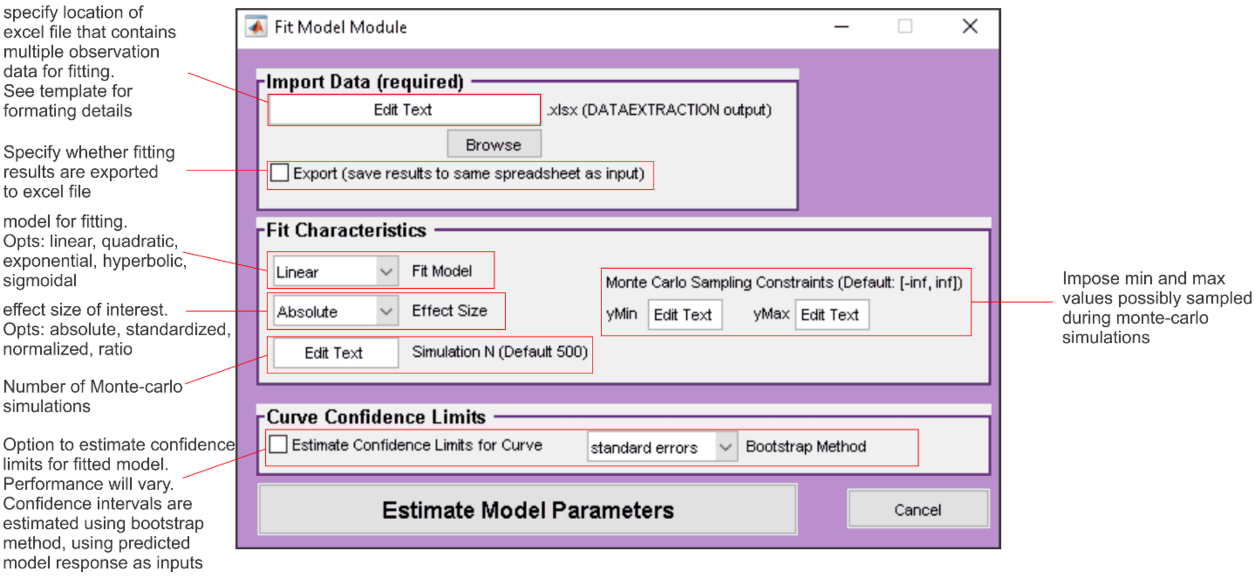

Figure 4. Fit model module user interface

Usage

Input. As input, the Fit Model Module requires a spreadsheet where study-level data sets are provided in separate sheets (**Fig. 4**). Each data set must consist of multiple independent observations organized with the following column headers (headers are case sensitive, see **Fig. 5** for template):

<u>Required Headers</u>

Study: name of study (string)

exposure: predictor variable (number)

xr: response variable (number)

ser: standard error of xr (number)

nr: sample size of xr (number)

<u>Optional Headers</u>

xc: basal/control variable (number)

sec: standard error of xc (number)

nc: sample size of xc (number)

Tip 6: Data exported in 'multiple' export format from Data Extraction Module (**Fig. 5)** is compatible as input into Fit Model Module. Otherwise, spreadsheet must be manually prepared by user.

Tip 7: To fit a model to standardized, normalized or ratio data, the optional control variable inputs (i.e., 'xc', 'sec' and 'nc') are necessary to compute the effect size of interest prior to fitting the data.

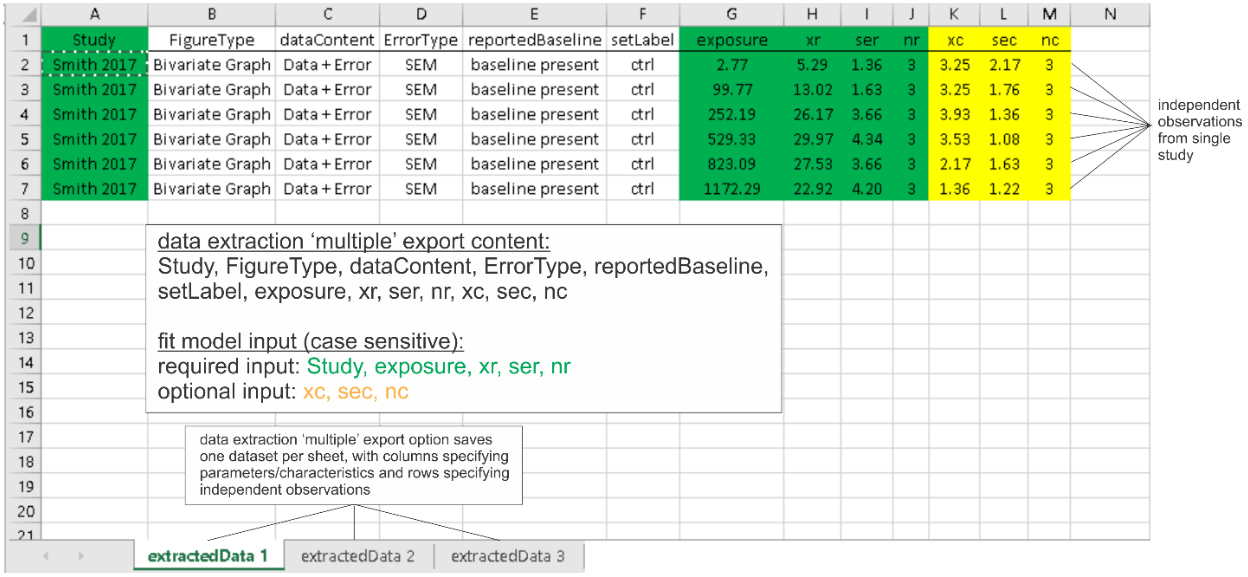

Figure 5. Fit model input data format in spreadsheet is shown. The format of exported results from the Data Extraction Module, with 'multiple' export option selected is shown. Manually prepared spreadsheet should follow same format.

Specifying Fit Characteristics: Data can be fit to linear, quadratic, exponential, hyperbolic or sigmoidal model and parameters computed in terms of absolute, normalized, response ratio or Hedges' standardized effect size **(Fig. 4)**. Parameter estimates will improve as number of simulations N increases (processing time increases with number of simulations, i.e., from seconds to minutes). Restrictions on sampled values can be imposed as yMin and yMax. To generate confidence intervals, standard errors or 95% percentiles are bootstrapped. Standard error-derived intervals are parametric and will be symmetrical, while percentile-derived intervals are non-parametric and can accommodate non-symmetrical intervals.

Tip 8: Lower bound restriction at 0 (yMin = 0, yMax = inf) is advised if experimental values are known to be non-negative.

Model Parameter Estimation. Upon initiating Fit Model Module, Meta Lab will proceed with Monte-Carlo model fitting. For each set of study-level data, user is prompted to visually evaluate fit.

In cases where fitting algorithm fails to converge to solution, new set of data will be resampled and fitting procedure will be attempted again. This will continue until the desired number of fits (specified by user are attained. Data that is incompatible with the specified model may result in MetaLab running indefinitely. If solutions are not found within ~ 2-5 minutes, cancel the fitting procedure and ensure the input data set is compatible with the specified model, otherwise select a different model or omit data set.

Tip 9: When prompted to evaluate quality of model fit, this is good opportunity to save figures of interest since figures are not automatically saved by MetaLab.

Output(s): For each data set (i.e., sheet in input spreadsheet), MetaLab will generate figures of (i) initial input data, (ii) Monte-Carlo samples and model fittings, (iii) distributions for estimated parameters and (iv) final model with reported fit results that include model name, function, mean estimates and standard errors.

If export option was specified, results are exported into same spreadsheet file that was initially specified (i.e., spreadsheet with input datasets). Otherwise, only figures are generated.

Example plots are provided as unmodified MetaLab outputs:

(i) Input data

Study-level data means (y) ± standard errors are plotted with respect to exposure (x). Study-level means and variances are used to approximate distributions from which data are resampled and fit accordingly.

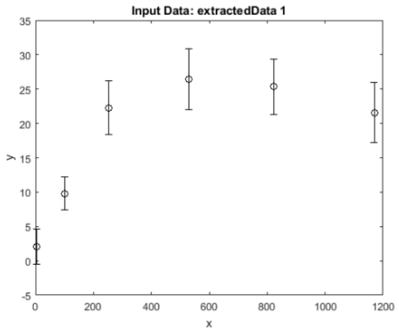

(ii) Monte-Carlo samples and model fits

Monte-Carlo sampled data is plotted along with corresponding model fits (*left*). Outlying fits are omitted (*right*) prior to estimation of final model parameters. Outliers are identified using fitted model parameters. Parameters that exceeded three median absolute deviations away from the median are omitted. See MATLAB isoutlier() function for more details on outlier detection.

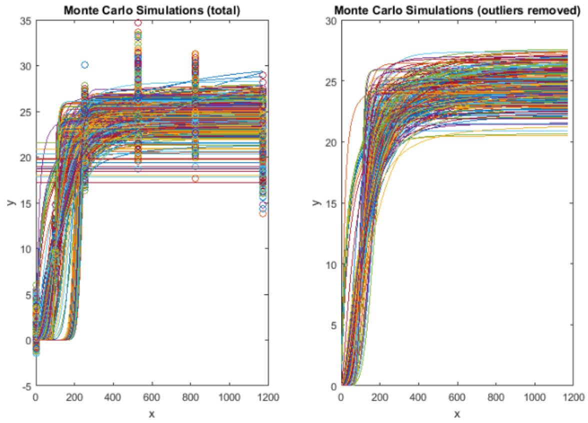

(iii) Model parameter distributions

For each set of Monte-Carlo samples, specified model is fit to data and set of parameters are extracted. Distribution of all fitted parameters are shown. Parameter error terms are derived from these distributions. Normally distributed parameter estimates are typically indicative of reliable parameter estimation.

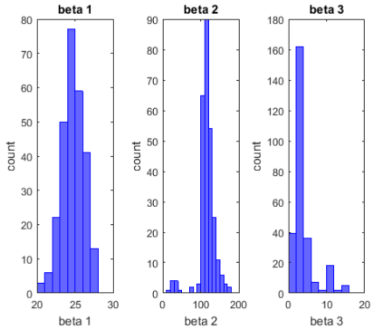

<u>(iv) Final model</u>

Final model (red curve) and study-level data (black markers) are shown. Optionally, 95% confidence limits are bootstrapped and shown (red bands). Summary of the fit results are displayed, including type of model, model function, parameter means and standard errors along with corresponding R^2 value. Parameters β are numbered as shown in model function, and mean and standard errors are listed in order (i.e. means for β_1, β_2, β_3, etc.). These statistics are exported to same spreadsheet as original input if export option was specified.

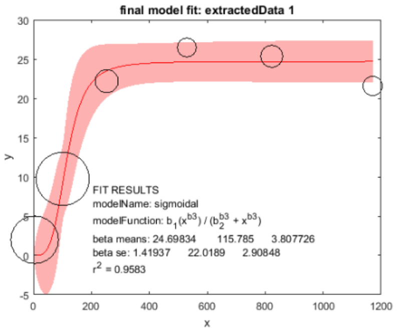

3. Prepare Data Module

Overview: Once study-level data has been extracted and prepared (Data Extraction and/or Fit Model Modules), this module will import the entire meta-analytic data set from a spreadsheet into MATLAB and save it in a standardized MATLAB format that is compatible with Heterogeneity, Meta-analysis and Meta-regression modules. This module also enables optional data stratification for downstream stratified subgroup analysis.

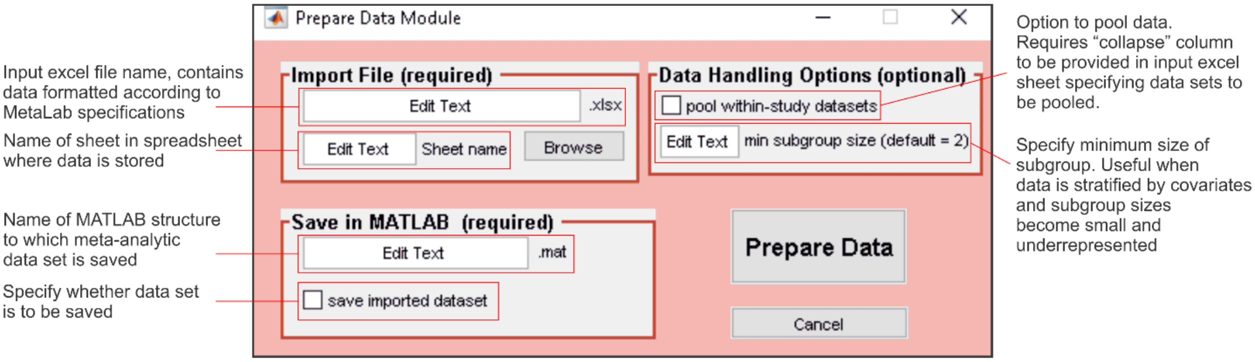

Figure 6. Prepare data module user interface

Usage

Input: To import meta-analytic data set into a MATLAB structure (**Fig 6**), data must be prepared in a spreadsheet according to MetaLab specifications, as shown in **Fig 7**. All data must be stored in a single spreadsheet, with the following column headers (headers are case sensitive):

<u>Required Headers</u>

ID: unique numeric identified for each independent observation

Study: name/label for study (string)

xr: response variable (number)

- Common units of measure must be used for study-level means if the absolute mean/difference is going to be used as effect size. If normalized, standardized or ratio effect sizes will be used, xc, sec and nc are required as inputs as well, however in these cases common units of measure are not required.

ser: standard error of xr (number)

nr: sample size of xr (number)

<u>Optional Headers</u>

xc: basal/control variable (number)

sec: standard error of xc (number)

nc: sample size of xc (number)

collapse: specifies which data sets are to be pooled together

- If the study contains multiple datasets, they can be either pooled together, or treated as independent observations. By default, MetaLab will treat each row as an independent observation. However multiple observations can be pooled together by adding a column in the excel sheet labeled "collapse", where unique numbers are assigned to the datasets that are treated as independent observations, while observations intended to be pooled together receive common values. MetaLab will pool data by calculating weighted means, using sample sizes as weights.

ISR: Specifies which data are grouped together for intrastudy regression (ISR)

- If reviewers wish to perform ISR analysis, additional column titled "ISR" must be included and numbered like the "collapse" column; Data that belong to same data set (i.e., within-study dataset) are designated by common numbers.

Covariates: Additional columns that have not been specified above can be included as covariates

- Categorical covariates must be coded numerically (ex. human – 1; mouse – 2; rat – 3) if reviewers wish to use these in subsequent data stratification or subgroup analysis.
- Current version of MetaLab will treat numerical continuous covariates like numerically-coded categorical covariates for subgroup analysis, however can be used in meta-regression analysis as continuous predictor.

To summarize, the minimum input excel sheet must contain "ID", "Study", "xr", "ser" and "nr" headers with optional columns including "xc", "sec", "nc", "collapse", "ISR", and any additional columns that are to be treated as covariates. Do not include unnecessary columns or rows input. Everything in input spreadsheet will be included part of the meta-analytic data set.

	A	B	C	D	E	F	G	H	I	J
1	ID	Study	xr	ser	nr	collapse	ISR	categorical covariate	continuous covariate	
2	1	Chen2008Fig3C.png	23.03609	0.796768	3	1	1	1	0	
3	2	Chen2008Fig3C.png	19.55603	1.586798	3	1	1	1	1	
4	3	Chen2008Fig3C.png	17.54757	1.952982	3	2	1	1	2	
5	4	Chen2008Fig3C.png	18.71036	1.098552	3	2	1	1	3	
6	5	Chen2008Fig3C.png	35.57895	2.430949	3	2	1	1	4	
7	6	Chen2008Fig3C.png	32.66385	4.028025	3	2	1	1	5	
8	7	Chen2008Fig3C.png	32.55814	3.722872	3	2	1	1	6	
9	8	Chen2008Fig3C.png	36.54661	3.363799	3	3	1	1	14	
10	9	Chen2008Fig3C.png	38.31579	1.64089	3	3	1	1	21	
11	10	Chen2008Fig3C.png	40.90909	2.197105	3	3	1	1	28	
12	11	Choi 2011 Toxicology in Vitro 25 1603–1608	5.82	0.36	3	4	2	3	3	
13	12	Choi 2014 Free Radical Research, 48:7, 729-739	Have	0.04825	3	5	2	3	2	
14	13	Choi 2007	0.103	0.005	3	6	2	3	2	
15	14	Dowd 1990 J Biol Chem 265 34 20833-20838	10.5	2	3	7	3	2	0	
16	15	Esen2013Fig2F.png	3.952381	0.568186	3	8	4	3	1	
17	16	Hsu2013Fig3C.png	30.50093	1.328227	3	9	5	1	21	
18	17	Hsu2013Fig3C.png	34.73098	0.814075	3	9	5	1	21	
19	18	Komarova2000Fig6.png	88.23529	6.417112	4	10	6	2	3	
20	19	Komarova2000Fig6.png	64.28571	12.32143	4	10	6	2	7	

Input format for Prepare Data Module[&]

required input: ID[$], Study, xr, ser, nr
optional input: xc*, sec*, nc*, collapse, ISR
additional inputs: treated as covariates[#]

[&]Header labels are case sensitive
[$]ID must be unique for each row
*omit headers for control variable if not available/required
[#]categorical covariates must be numerically coded, no restrictions imposed on what covariate header labels can be

Figure 7. Prepare data input format in spreadsheet. Required and optional headers are shown.

***Data stratification*:** Once Prepare Data Module has been initiated, user will be prompted to optionally stratify data by categorical covariates (**Fig 8**). This has been included as option to conduct stratified-subgroup analysis, however is not necessary and users can proceed by selecting "no stratification" option.

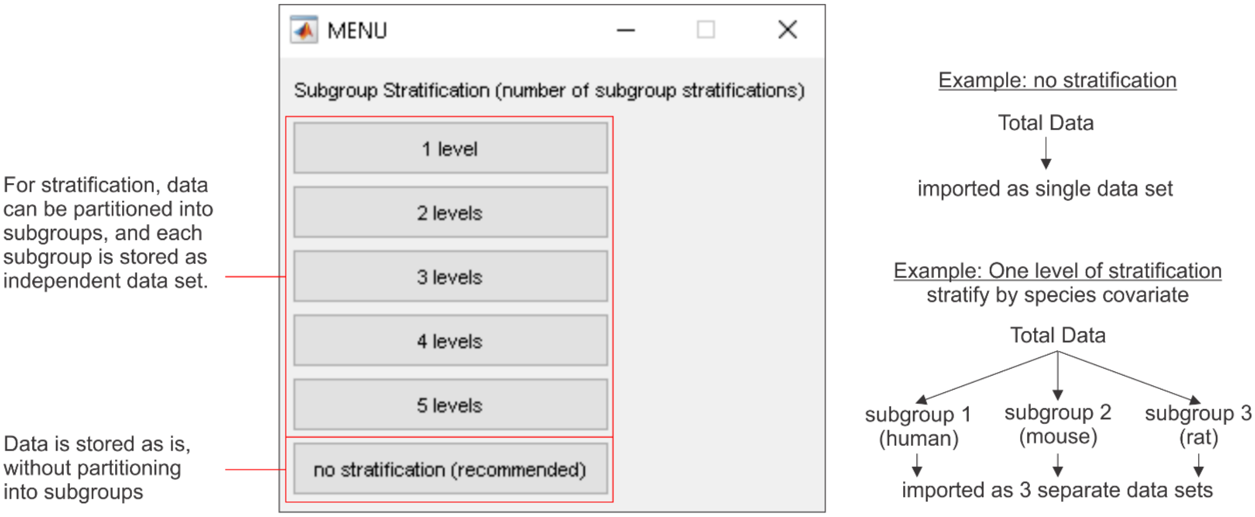

Figure 8. Data sets can be stratified according to categorical covariates in Prepare Data Module. Once level of stratification is selected, users can select from list of available covariates to stratify data by.

***Output*:** Imported data will be saved into MATLAB structure and is now ready for analysis in subsequent MetaLab modules. Multiple data sets can be saved to the same MATLAB structure by simply specifying that newly imported data be saved to a pre-existing MATLAB data structure. When these data structures are used in subsequent analyses (Heterogeneity, Meta-Analysis or Meta-Regression Modules), MetaLab will produce results for each data set independently.

The following prompt will be displayed when data has been successfully imported:

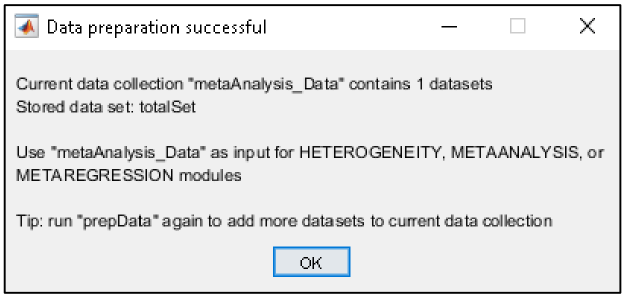

4. Heterogeneity Module

Overview: This module is designed to assess the extent of heterogeneity and bias present in a meta-analytic data set. It can be used to generate study-level data distributions, funnel plots and Baujat plots, evaluate variance/sample size assumptions, compare weighting schemes and tau^2 estimates and conduct sensitivity analyses and cluster-covariate dependence analyses.

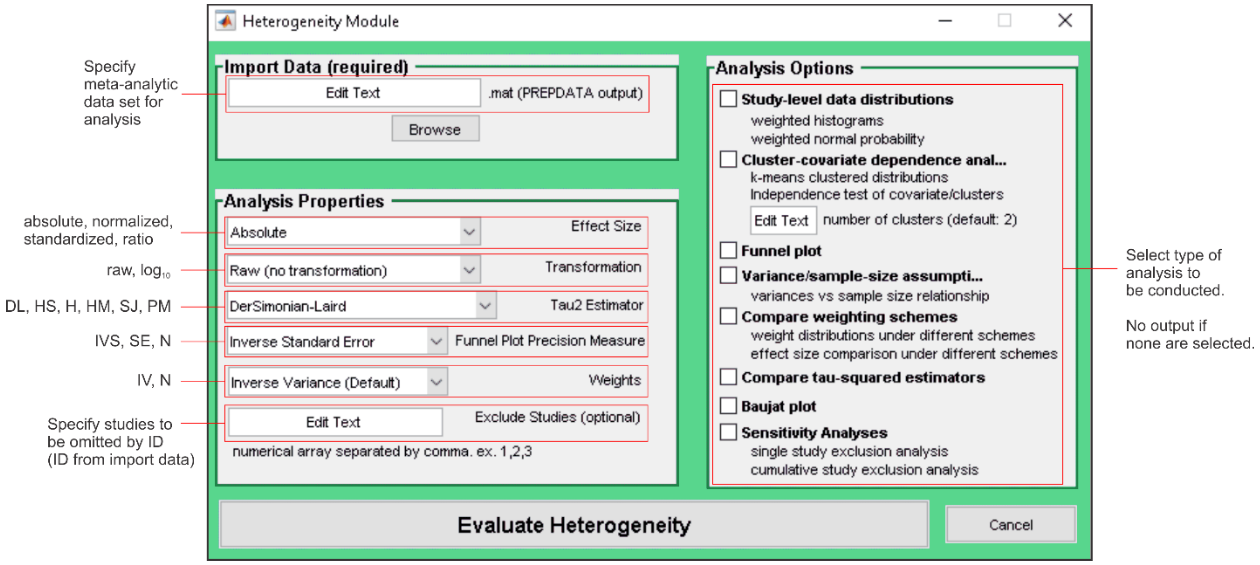

Figure 9. Heterogeneity module user interface. Tau^2 estimator options are DL: DerSimonian-Laird, HS: Hunter-Schmidt, H: Hedges, HM: Hatung-Makambi, SJ: Sidik-Jonkman, PM: Paule-Mandel. Funnel plot precision measures options are IVS: inverse standard error, SE: standard error and N: sample size. Weighting options are IV: inverse variance and N: sample size.

Usage

Input. Meta-analytic data set(s) that have been imported using the Prepare Data Module can be directly loaded into the Heterogeneity module. Several analysis options are provided along with analysis properties (**Fig 9**).

Typical settings used in meta-analyses are set as default. Analyses will be conducted using specified effect size and transformation method.

***Output*:** Heterogeneity module will produce figures for each set of specified analyses, which can then be manually saved. Plots that are provided below are unmodified outputs from MetaLab.

Study-level data distributions

Weighted histograms (unweighted, fixed effects weighted and random effects weighted).

Weighted normal probability plots (unweighted, fixed effects weighted and random effects weighted)

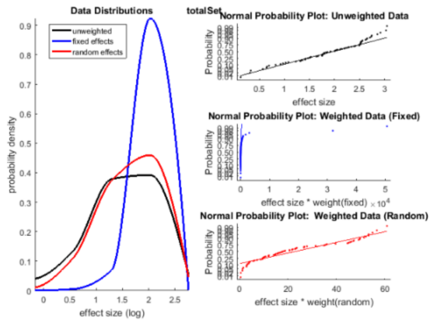

Cluster-covariate dependence analyses

Data are clustered according to k-means algorithm and distributions of clusters are visualized using histograms. Default number of clusters is two however can be modified if more clusters are expected.

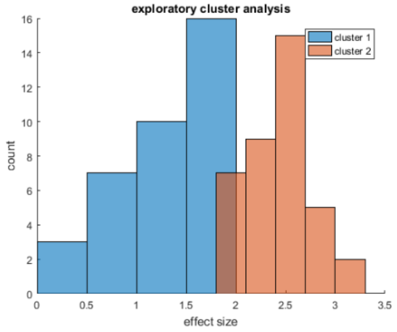

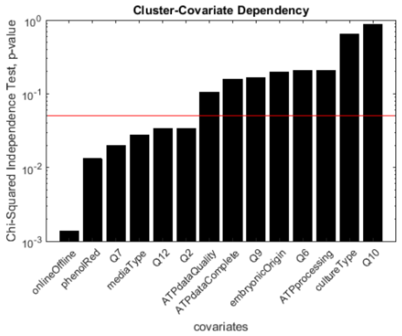

p-values for χ^2-test of independence between cluster membership and covariates are plotted (*black bars*) in ascending order with significance threshold (*red*) shown for reference. This analysis is useful for identifying potentiate covariates of interest, which may account for observed heterogeneity.

Funnel plot

Study-level precisions are plotted with respect to study-level effect sizes (*black markers*). Precision measure (inverse standard error, standard error or sample size) along with transformation (raw or $\log_{10}$) of effect size can be specified. Theoretical 95% confidence intervals (*black curves*) are centered around fixed effects estimate (*blue*), and random effects estimate (*red*) is shown for reference.

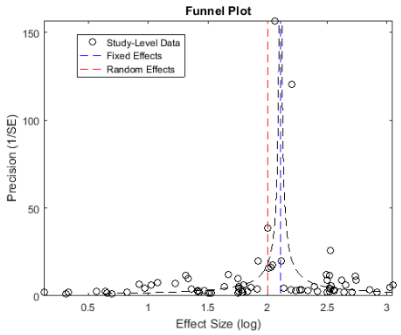

Variance/sample size assumption

Study-level variances (squared standard deviation, not squared standard errors) are plotted with respect to sample size, and monotonic relationship is evaluated. Used to test independence assumption between study level variance and sample size. If relationship exists, alternative weighting scheme may be considered (i.e., sample size weighting)

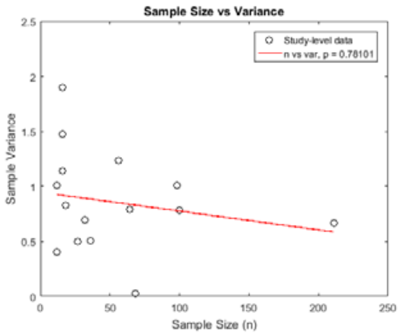

Comparison of weighting schemes

Distribution of weights across studies are visualized, comparing unweighted (UW), fixed effects weighting (FE), random effects weighting (RE) and sample size weighting (N). Proportion of total weight is plotted on Y-axis.

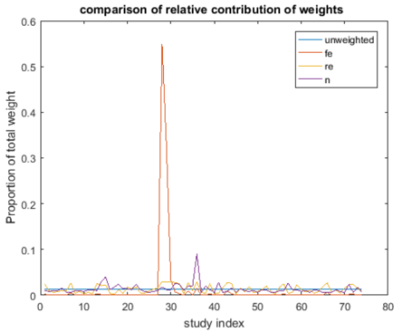

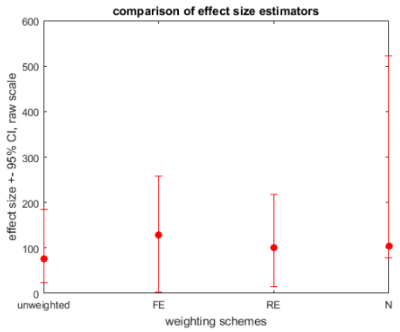

Effect size estimates ± 95% confidence intervals are compared between weighting schemes. If data was synthesized on logarithmic scale, effect size estimates are back-transformed and presented on raw scale.

Comparison of τ^2 estimators

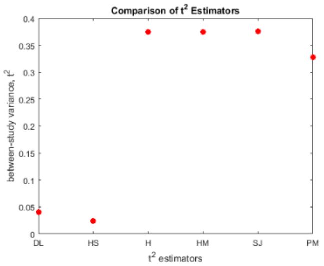

τ^2 estimates are computed using different estimators and compared with one another. τ^2 estimators are DL: DerSimonian-Laird, HS: Hunter-Schmidt, H: Hedges, HM: Hatung-Makambi, SJ: Sidik-Jonkman, PM: Paule-Mandel

Baujat plot

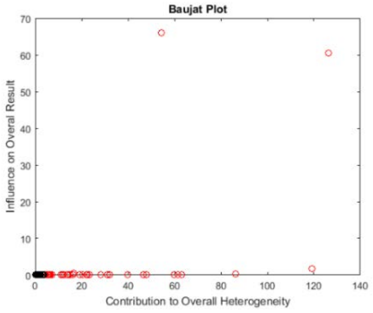

Extent of influence each study has on heterogeneity (x-axis) and effect size (y-axis) are plotted and outlying studies can be identified in the upper right corner of the plot.

Studies that have significant influence on heterogeneity are plotted in red, as determined by Q-test.

Sensitivity analyses

Single study exclusion plots reveal influential studies, as determined by shift in effect size and Q, H^2 and I^2 heterogeneity statistics after exclusion of single studies. Cumulative study exclusion plots are generated to visualize how effect size and Q, H^2 and I^2 heterogeneity statistics shift with cumulative exclusion of studies according to Q-reduction criteria. Homogeneity threshold T_H is indicated.

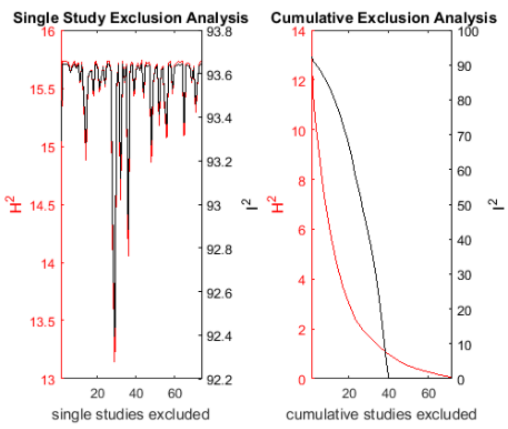

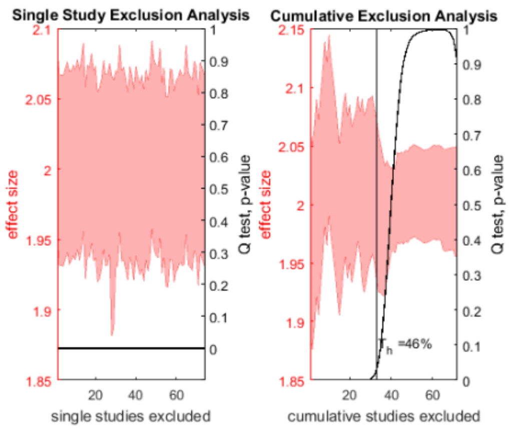

5. Meta-Analysis Module

Overview: Synthesize meta-analytic data and conduct subgroup analysis if covariates present.

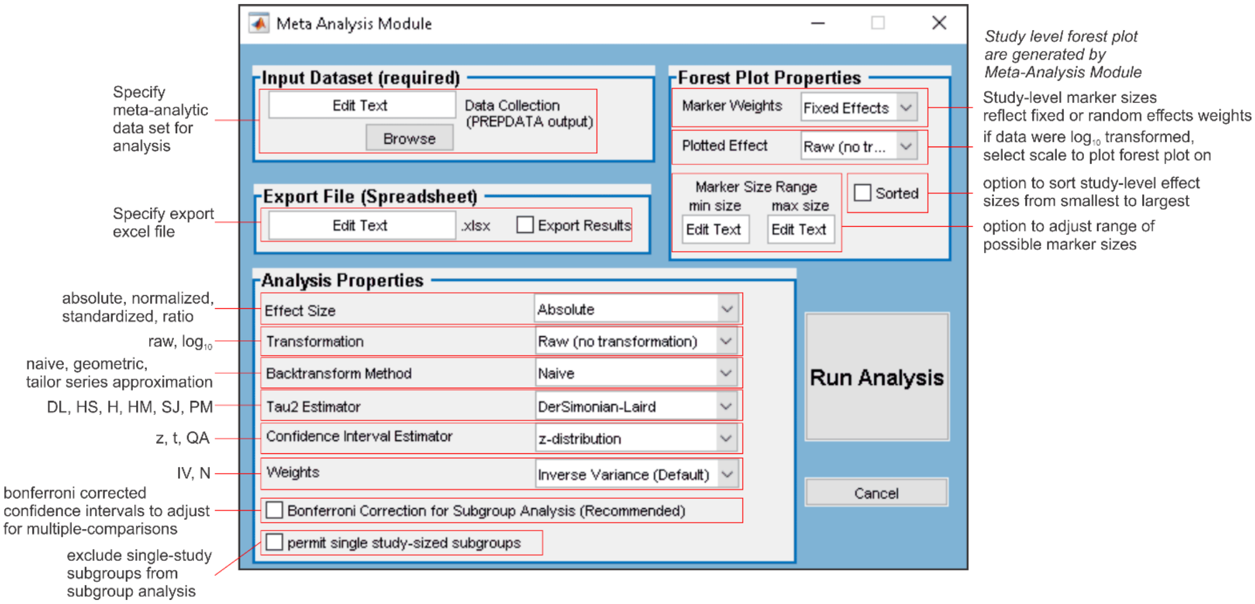

Figure 10. Meta-analysis module user interface. Tau2 estimator options are DL: DerSimonian-Laird, HS: Hunter-Schmidt, H: Hedges, HM: Hatung-Makambi, SJ: Sidik-Jonkman, PM: Paule-Mandel. Confidence interval estimator options are z: z-distribution, t: t-distribution or QA: quantile approximation method. Weighting options are IV: inverse variance weighting or N: sample size weighting. options are IVS: inverse standard error, SE: standard error and N: sample size.Weighting options are IV: inverse variance and N: sample size. Back-transformation methods are used when data are $\log_{10}$ transformed.

Usage

Input: Meta-analytic data set(s) that have been imported with Prepare Data Module can be directly loaded into the Meta-Analysis module. Several analysis options are provided along with analysis properties (**Fig. 10**). Typical settings used in meta-analyses are set as default.

Subgroup Analyses: Meta-analysis Module will always proceed to conduct subgroup analysis if covariates are present in data set (see Prepare Data Module for details about including covariates in data set). That is, for a given data set, Meta-Analysis Module will estimate the overall effect size for the total data set (labelled 'totalSet' in output spreadsheet) along with subgroup estimates for each available covariate. This is the simplest form of subgroup analysis which can be extended to accommodate multiple levels of stratification using the stratification option in the Prepare Data Module. For example, if a data set is stratified by 'Species' covariate, the data is partitioned into human, mouse and rat data subsets which are then saved in a single MATLAB data structure as three independent data sets. When this MATLAB structure loaded into the Meta-Analysis Module, the overall effect size will be estimated for each of the three independent data sets (i.e., human, mouse and rat subsets) along with subgroup estimates nested within these data subsets. Hence, the spreadsheet output will report subgroup estimates nested within each of the species-level data subsets, rather than for the total unpartitioned data set. Note that subgroup estimates are only saved in spreadsheet, and are not represented graphically by the Meta-Analysis Module. Only the 'totalSet' (i.e., overall effect size for given data set) is illustrated in the output study-level forest plot.

Tip 10: While subgroup analysis is useful for identifying sources of heterogeneity, extensive data stratification can result in a diluted data pool, leading to subgroup under-representation and erroneous findings.

Output: Meta-analysis module will produce unweighted distributions (*left*) with specified data transformation and forest plot (*right*) representing study-level effect sizes along with global fixed (*blue band*) and random (*red band*) effects estimates. Y-axis labels correspond with study-level IDs. Example figures are provided as unmodified MetaLab outputs:

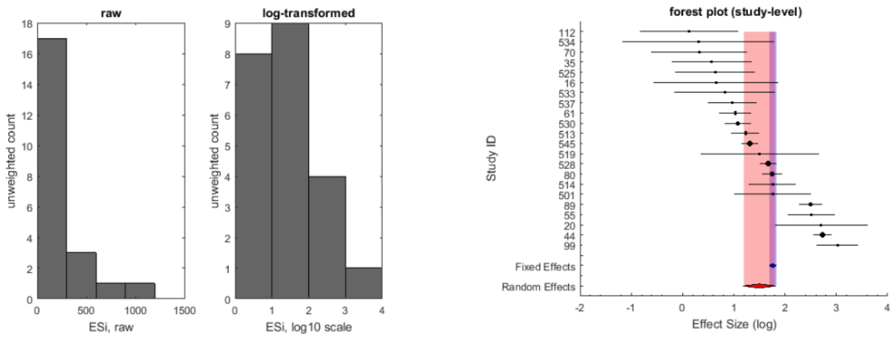

If export option was selected and export file name was specified, results along with input data will be exported to spreadsheet where more detailed statistics are provided, including heterogeneity statistics (I^2, H^2, Q), between-study variance estimate (t^2) and effect size estimates with error terms (**Figure 11**).

meta-analysis of total data set → Total; subgroup analysis of "method" covariate*. → method_1, method_2

Heterogeneity and Performance Statistics (rows I2 to coverage_fe); Meta-Analytic Effect Size Estimates (rows ESre to ciEstimator).

If data was synthesized on log_{10} scale, exported results will be reported on log_{10} scale (green) and back-transformed estimates on raw scale (pink)

in the absense of log_{10} transformation, only one set of estimates will be reported on raw scale

bonferroni adjusted confidence intervals are reported only if option is specified. Will influence width of confidence intervals when there are >2 subgroups per covariate

name of dataset	subgroup	Total	method_1	method_2
I2 heterogeneity statistic	I2	93.56	94.15	92.38
I2 lower bound 95% confidence interval	I2lo	93.05	93.30	91.62
I2 upper bound 95% confidence interval	I2hi	94.03	94.90	93.07
H2 heterogeneity statistic	H2	15.52	17.09	13.13
degrees of freedom	df	73.00	21.00	51.00
Q heterogeneity statistic	Q	1133.14	358.99	669.50
Q-test p-value	Qp	0.00	0.00	0.00
number of subgroups for given covariate	nSubgroups	1.00	2.00	2.00
c-scaling variable (used for t2 calculation)	C	26499.50	759.53	25291.99
tau2 estimator	tauEstimator	DL	DL	DL
t2 estimate	t2	0.04	0.44	0.02
random effects confidence interval coverage	coverage_re	6.76	31.82	7.69
fixed effects confidence interval coverage	coverage_fe	1.35	13.64	1.92
log-transformed random effect estimate	ESre	2.00	1.50	2.13
random effect standard error	SEre	0.03	0.16	0.03
log-transformed fixed effects estimate	ESfe	2.11	1.76	2.11
fixed effects standard error	SEfe	0.00	0.03	0.00
random effects 95% confidence interval width	ciWidth_re	0.07	0.31	0.07
fixed effects 95% confidence interval width	ciWidth_fe	0.01	0.07	0.01
confidence interval critical value	critValue	1.96	1.96	1.96
bonferroni corrected confidence interval (random effects)	adjCiWidth_re	0.07	0.31	0.07
bonferroni corrected confidence interval (fixed effects)	adjCiWidth_fe	0.01	0.07	0.01
bonferroni corrected confidence interval critical value	adjCritValue	1.96	1.96	1.96
confidence interval estimator	ciEstimator	zdist	zdist	zdist
back-transformed random effects estimate	ESre_raw	100.51	32.46	136.27
random effects standard error	SEre_raw	27.65	62.64	51.01
random effects lower-bound 95% confidence interval	ciLre_raw	86.04	15.89	116.61
random effects upper-bound 95% confidence interval	ciUre_raw	117.42	66.34	159.23
bonferroni-corrected random effects lower-bound 95% confidence interval	adjCiLre_raw	86.04	15.89	116.61
bonferroni-corrected random effects upper-bound 95% confidence interval	adjCiUre_raw	117.42	66.34	159.23
back-transformed fixed effects estimate	ESfe_raw	127.97	57.69	129.98
fixed effects standard error	SEfe_raw	0.84	8.98	0.89
fixed effects lower-bound 95% confidence interval	ciLfe_raw	125.27	49.43	127.21
fixed effects upper-bound 95% confidence interval	ciUfe_raw	130.73	67.32	132.81
bonferroni-corrected fixed effects lower-bound 95% confidence interval	adjCiLfe_raw	125.27	49.43	127.21
bonferroni-corrected fixed effects upper-bound 95% confidence interval	adjCiUfe_raw	130.73	67.32	132.81
back-transformation method	log2rawMethod	naive transformation	naive transformation	naive transformation

*method_1 & method_2 correspond with numerical coding of categorical covariate specified in initial data input for Prepare Data Module

Figure 11. Meta-analysis results exported to spreadsheet.

6. Meta-Regression Module

Overview: Conducts linear meta-regression analysis. Users can optionally validate between-study (meta)-regression results with intrastudy regression analysis, which conducts linear regression on within-study data sets.

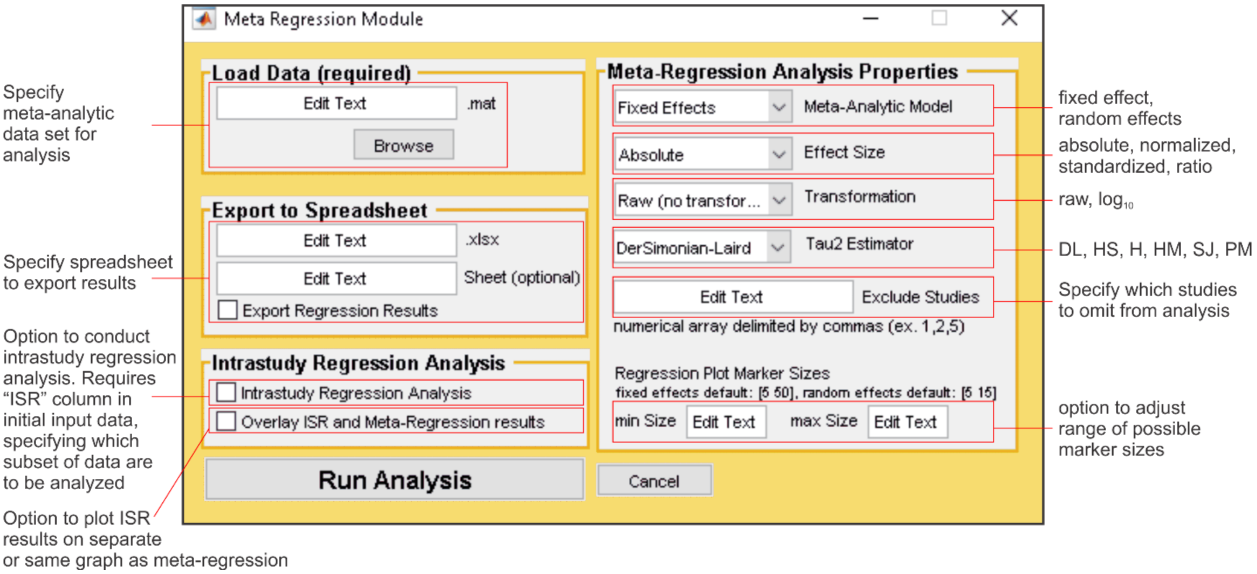

Figure 12. Meta-Regression module user interface. Tau2 estimator options are DL: DerSimonian-Laird, HS: Hunter-Schmidt, H: Hedges, HM: Hatung-Makambi, SJ: Sidik-Jonkman, PM: Paule-Mandel

Usage

Input: Meta-analytic data set(s) that have been imported with Prepare Data Module can be directly loaded into the meta-regression module (**Figure 12**). Continuous/categorical covariates must be included in dataset to conduct meta-regression analysis. If reviewers wish to perform intrastudy regression analysis (ISR) analysis, additional column titled "ISR" must be included when preparing data set from spreadsheet using Prepare Data Module. The ISR variable specifies which study-level data belong to the same data set such that within-study data sets that differ by level of exposure/predictor are designated by common values (see Prepare Data Module for details/example). Each study must have numerical value assigned in ISR column, those that do not have >2 observations will be automatically omitted from ISR analysis.

Meta-regression analysis: One meta-regression analysis has been started, MetaLab requires users to specify the outcome of interest along with predictor variables (**Figure 13**). The effect size of interest will be labeled "Study-level outcome". MetaLab will additionally present all available covariates that were provided in the initial data input (See Prepare Data Module). Univariate and Multi-variate regression analyses are supported by MetaLab. Linear regression models are created using MATLAB's fitlm() function.

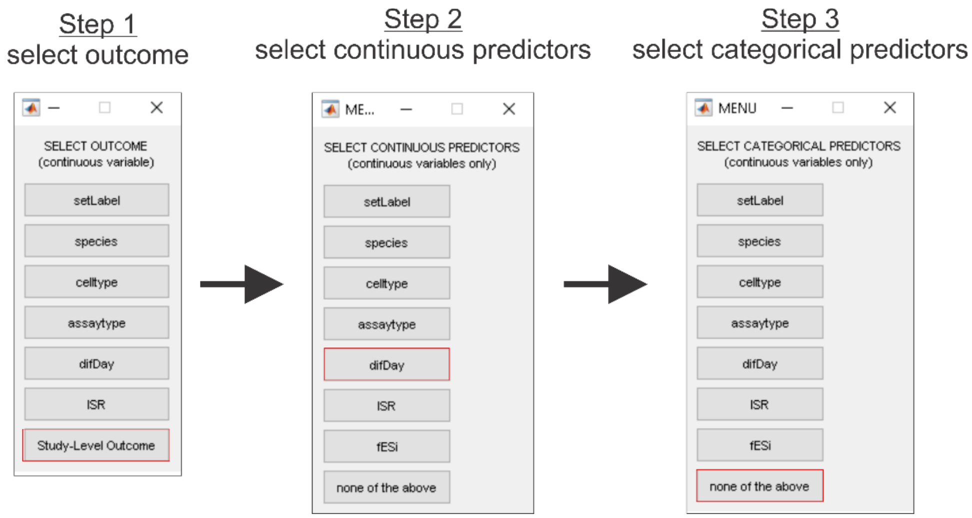

Figure 13. Specification of outcome and predictor variables for meta-regression analysis.

Output: Meta-regression module will produce regression diagnostic plots and meta-regression plots. Shown graphs are presented as unmodified MetaLab outputs:

Regression diagnostic plots

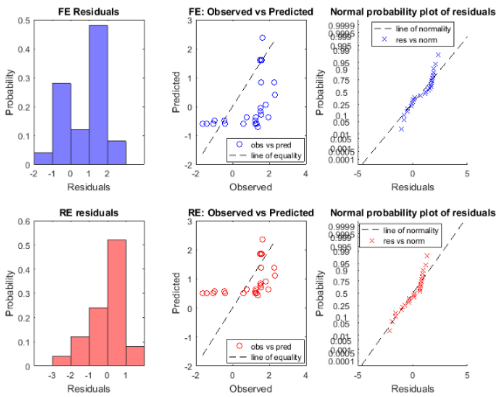

The regression diagnostic plot presents the distribution of regression residuals (histograms) and normal probability plots under the assumptions of a fixed (*blue*) and random effects (*red*) model. Moreover, observed outcomes are plotted in relation to model prediction, with the line of equality provided as a reference. Normally distributed residuals and agreement between observed and predicted values are indicative of a decent meta-regression model.

Linear meta-regression plot

The meta-regression plot shows study-level effect sizes (*black markers*), fitted meta-regression curve (*dashed black line*) with associated 95% confidence (dark red band) and prediction (*light red band*) intervals and 95% confidence interval for intercept-only model (*grey band*). Marker sizes are proportional to weights under the respective meta-analytic model (fixed or random effects). Optional ISR analyses are shown (overlaid as *red sold curves*). *difDay*: differentiation day, predictor variable. *fESi*: study-level effect sizes, outcome variable.

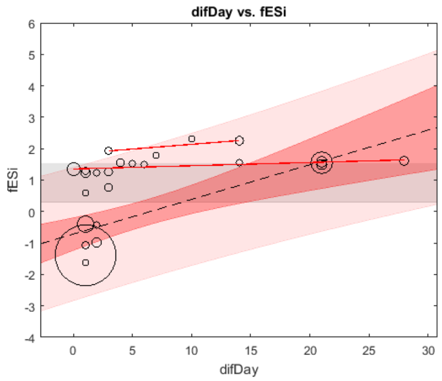

For multivariate meta-regression analyses, MetaLab will plot an adjusted response model for each given predictor variable. The fitted response is then a function of the predictor variable, with all other predictors averaged over the range of data used in the model. See MATLAB plotAdjustedResponse() function for more details.

If export option was selected and export file name was specified, meta-regression results will be exported to spreadsheet where more detailed statistics are provided (**Fig 14**). Model specifications are reported along with diagnostic statistics which include analysis of heterogeneity. Intercept only model statistics are shown for reference. Note that intercept-only model estimates may differ from those acquired in Meta-Analysis module due to exclusion of studies where predictor covariates are missing. If ISR analysis was conducted, slopes are compared with those acquired from meta-regression analysis. Validity of results are supported if ISR and meta-regression results are consistent (i.e. p-value > 0.05).

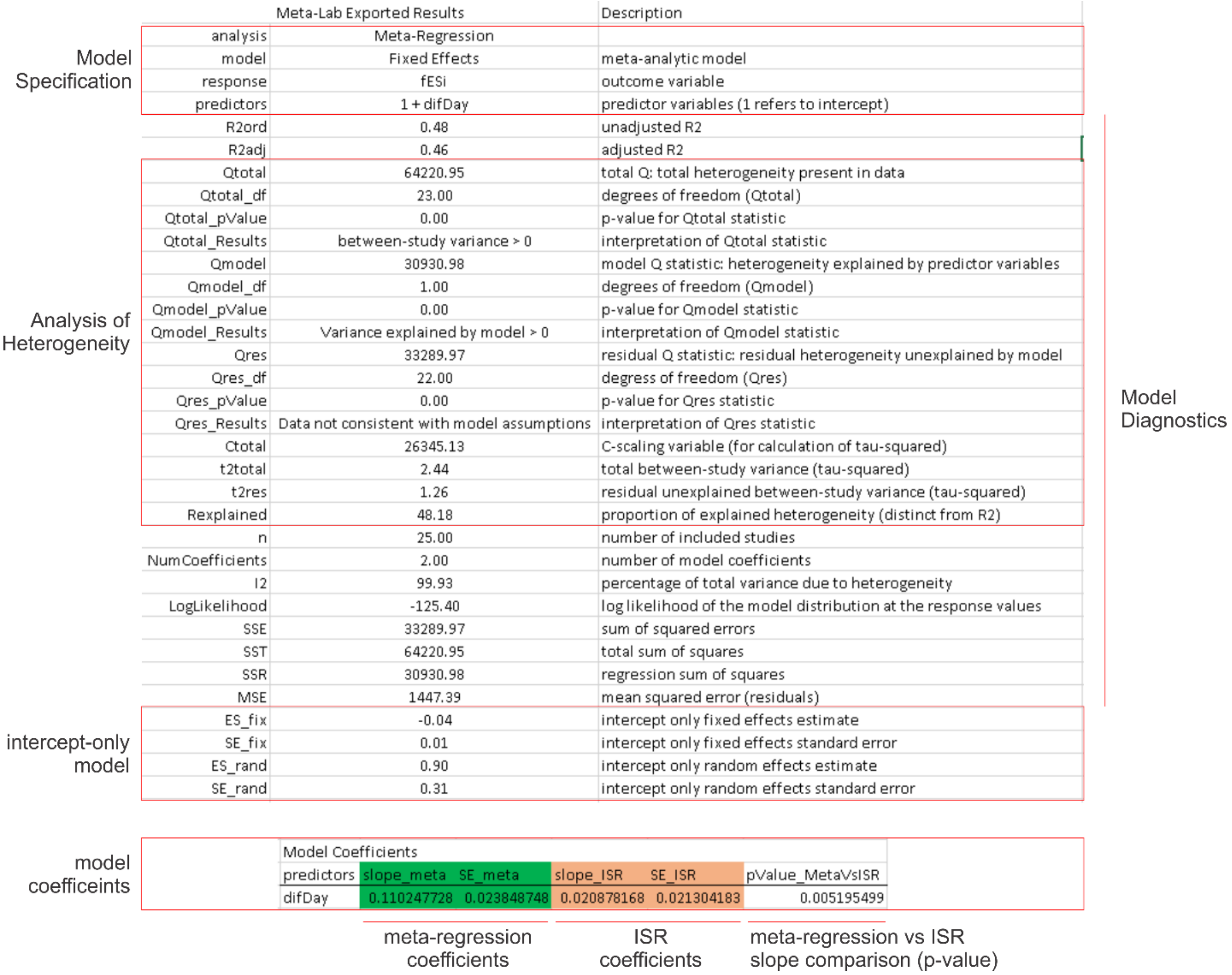

	Meta-Lab Exported Results	Description
analysis	Meta-Regression	
model	Fixed Effects	meta-analytic model
response	fESi	outcome variable
predictors	1 + difDay	predictor variables (1 refers to intercept)
R2ord	0.48	unadjusted R2
R2adj	0.46	adjusted R2
Qtotal	64220.95	total Q: total heterogeneity present in data
Qtotal_df	23.00	degrees of freedom (Qtotal)
Qtotal_pValue	0.00	p-value for Qtotal statistic
Qtotal_Results	between-study variance > 0	interpretation of Qtotal statistic
Qmodel	30930.98	model Q statistic: heterogeneity explained by predictor variables
Qmodel_df	1.00	degrees of freedom (Qmodel)
Qmodel_pValue	0.00	p-value for Qmodel statistic
Qmodel_Results	Variance explained by model > 0	interpretation of Qmodel statistic
Qres	33289.97	residual Q statistic: residual heterogeneity unexplained by model
Qres_df	22.00	degress of freedom (Qres)
Qres_pValue	0.00	p-value for Qres statistic
Qres_Results	Data not consistent with model assumptions	interpretation of Qres statistic
Ctotal	26345.13	C-scaling variable (for calculation of tau-squared)
t2total	2.44	total between-study variance (tau-squared)
t2res	1.26	residual unexplained between-study variance (tau-squared)
Rexplained	48.18	proportion of explained heterogeneity (distinct from R2)
n	25.00	number of included studies
NumCoefficients	2.00	number of model coefficients
I2	99.93	percentage of total variance due to heterogeneity
LogLikelihood	-125.40	log likelihood of the model distribution at the response values
SSE	33289.97	sum of squared errors
SST	64220.95	total sum of squares
SSR	30930.98	regression sum of squares
MSE	1447.39	mean squared error (residuals)
ES_fix	-0.04	intercept only fixed effects estimate
SE_fix	0.01	intercept only fixed effects standard error
ES_rand	0.90	intercept only random effects estimate
SE_rand	0.31	intercept only random effects standard error

Model Coefficients

predictors	slope_meta	SE_meta	slope_ISR	SE_ISR	pValue_MetaVsISR
difDay	0.110247728	0.023848748	0.020878168	0.021304183	0.005195499

Figure 14. Meta-regression results exported to spreadsheet.

MATLAB Tips

Useful MATLAB Tips

Here we provide a selection of MATLAB operation-related tips that may come in handy while using MetaLab:

When initiating MetaLab and presented with this dialog window…

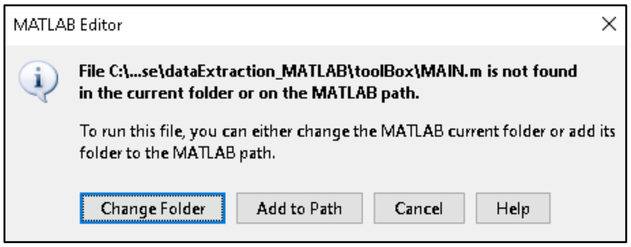

This dialog will be presented if MetaLab is not in MATLAB's current folder. Simply press 'Change Folder' and the MATLAB will automatically redirect the current folder to where MetaLab files are found.

To terminate MetaLab while it is running…

Press 'Ctrl+C' on keyboard while MATLAB window is open and running

Useful when MetaLab has been running for an exceedingly long time (i.e., > 5 mins). Terminate the operation and check to see that inputs are all formatted according to MetaLab specifications.

To close all figures…

enter following into command line

>> close all

Prior to entering command through command line, no scripts can be running in background.

Certain MetaLab modules may generate many figures at a time. To minimize clutter and avoid having to close all figures one at a time, use the close all command.

To clear variables from workspace…

enter following into command line

>> clear all

Figures in MATLAB

The MATLAB graphical environment provides users with the flexibility to modify and save figures in a variety of formats. The native MATLAB figure format is *.fig and can be saved as such directly from the MATLAB figure window. For figures of interest, we recommend saving the original figure as a MATLAB figure prior to exporting in alternative formats.

To save figures…

1. For a given figure, go to File > Save As…

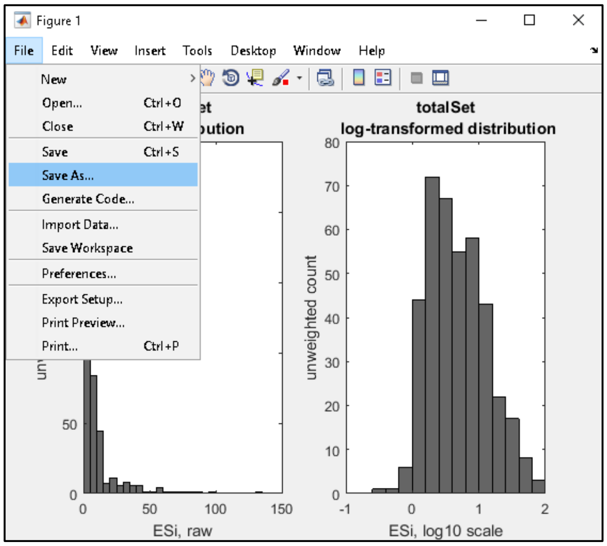

2. Specify save format and save figure.

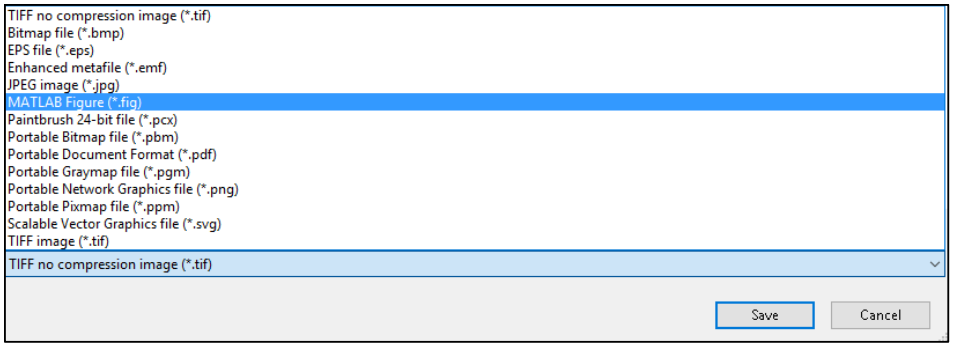

There are several available formats, enabling users to save MetaLab figures to raster (i.e., *.tif, *.bmp, *.png, etc.) or vector-based formats (i.e., *.svg, *.eps, etc.). Raster images (i.e. bitmap images) are composed of pixels, hence rescaling these will have an impact on the quality. Alternatively, vector-based images are constructed mathematically such that rescaling the images results in no loss of quality. Vector-based images are preferred for image editors such as Adobe Photoshop or Corel Draw due to superior quality and versatility. For vector-based images, we recommend saving MATLAB figures in *.svg format.

To modify figure output resolution…

1. Raster-based image resolutions are adjusted automatically by MATLAB. However, users can modify the resolution for a given figure by going to File > Export Setup

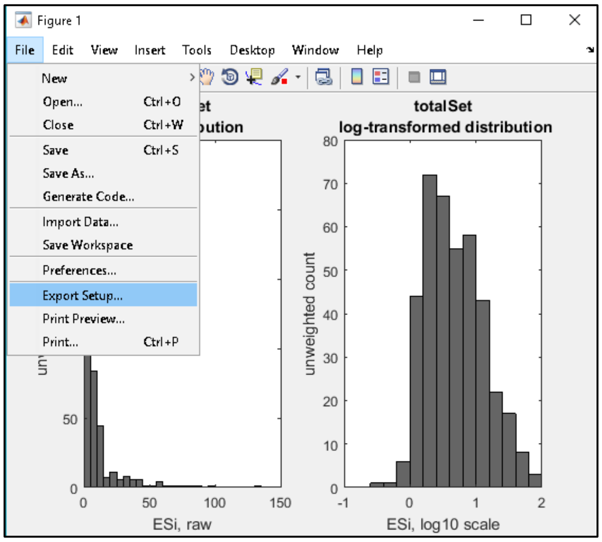

2. Under ‘Rendering Properties’, resolution can be changed. Other image properties of interest can also be found in this menu. Once changes have been made, click on ‘apply to figure’ and then ‘ok’.

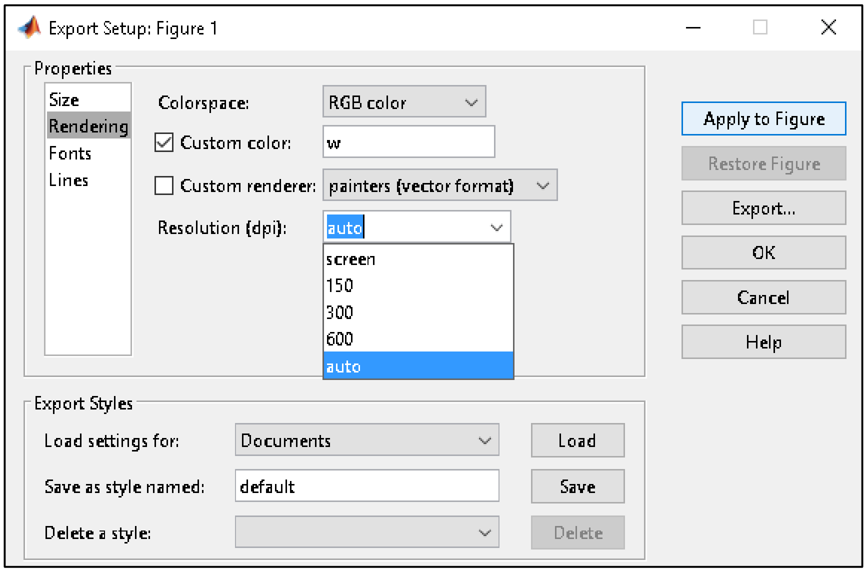

3. Once changes have been applied, users can save figure as specified above.

To customize figures using plot tools…

1. To customize figures directly in MaTLAB, select the ‘edit plot pointer’

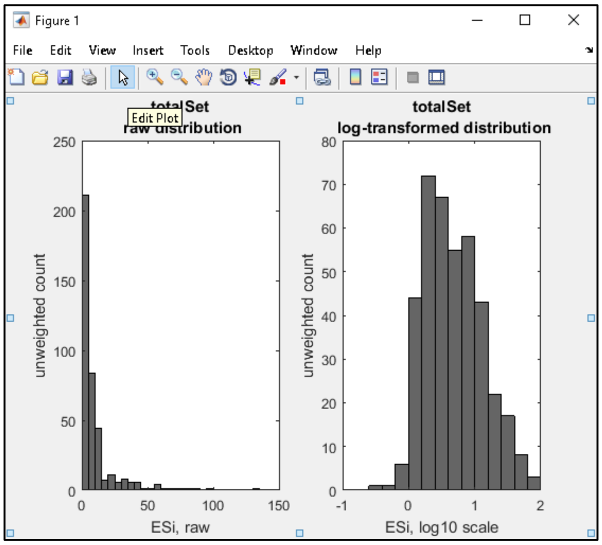

2. Double click on figure features to open property editor from which figure can be customized.

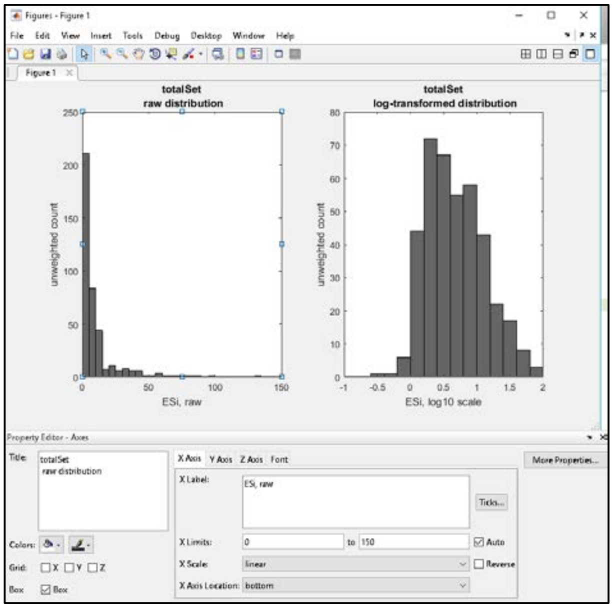

MATLAB Resources

For those interested in learning more about MATLAB, free resources are available online:

1. **MathWorks MATLAB documentation**: Detailed documentations MATLAB.
   https://www.mathworks.com/help/matlab/
2. **Tutorials point MATLAB tutorial**: MATLAB tutorial targeted towards beginners with little prior knowledge.
   https://www.tutorialspoint.com/matlab/
3. **University of Michigan MATLAB Quick MATLAB Tutorial**: Quick start guide with examples and further resources
   http://web.eecs.umich.edu/~aey/eecs451/matlab.pdf

Credit: Gowtham Bellala
