# Supplementary material for: Meta-Analytic Methodology for Basic Research: A Practical Guide
Source: Front Physiol. 2019 Mar 27;10:203. doi: 10.3389/fphys.2019.00203 (PMC6445886; doi:10.3389/fphys.2019.00203)
Supplement: Supplementary file 2 [file Data_Sheet_2.ZIP › working example/exampleCodingLegend.docx]

Coding Legend

# mechStim

**description**: type of mechanical stimulus applied to cells prior to measure ATP release kinetics

**variable type**: coded categorical

| **Code** | **Label** |
| --- | --- |
| 1 | FSS |
| 2 | Strain |
| 3 | Osmotic Pressure |
| 4 | Ultrasound |

# CyclicStimulus

**description**: mechanical stimulus was either cyclic or static

**variable type**: coded categorical

| **Code** | **Label** |
| --- | --- |
| 1 | Static stimulation |
| 2 | Cyclic stimulation |

# onOff

**description**: ATP release kinetics were measured either online (real-time recording) or offline (sample first, and measure later)

**variable type**: coded categorical

| **Code** | **Label** |
| --- | --- |
| 1 | Offline measurement |
| 2 | Online measurement |
