# Supplementary material for: Meta-Analytic Methodology for Basic Research: A Practical Guide
Source: Front Physiol. 2019 Mar 27;10:203. doi: 10.3389/fphys.2019.00203 (PMC6445886; doi:10.3389/fphys.2019.00203)
Supplement: Supplementary file 2 [file Data_Sheet_2.ZIP › working example/Working example.docx]

**Working example.** ATP release kinetics dataset (stored in example.xlsx) is used to illustrate a typical workflow using MetaLab. This example provides step-by-step instructions how to import data into MetaLab and evaluate heterogeneity and perform meta-analysis.

The analysis and interpretation of the dataset used in this working example has been published:

Mikolajewicz N., Mohammed A., Morris M., Komarova SV. (2018). Mechanically-stimulated ATP release from mammalian cells: systematic review and meta-analysis. *J Cell Sci*, 131(22). doi: 10.1242/jcs.223354.

**Prior to using MetaLab, data must be prepared:**

The example.xlsx spreadsheet has been prepared in compliance with specifications provided in the MetaLab User Guide.


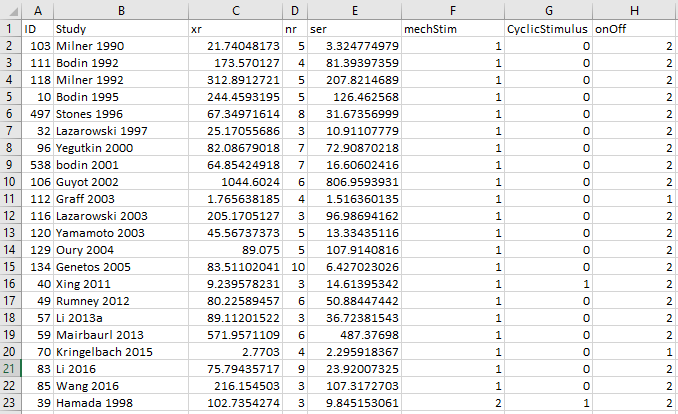


**ID**: study index, **Study**: study name, **xr**: effect size, **ser**: standard error, **nr**: sample size, **mechStim**: coded categorical covariate (optional), **CyclicStimulus**: coded categorical covariate (optional), **onOff**: coded categorical covariate (optional)

The corresponding coding legend is provided as a separate document (exampleCodingLegend.docx):


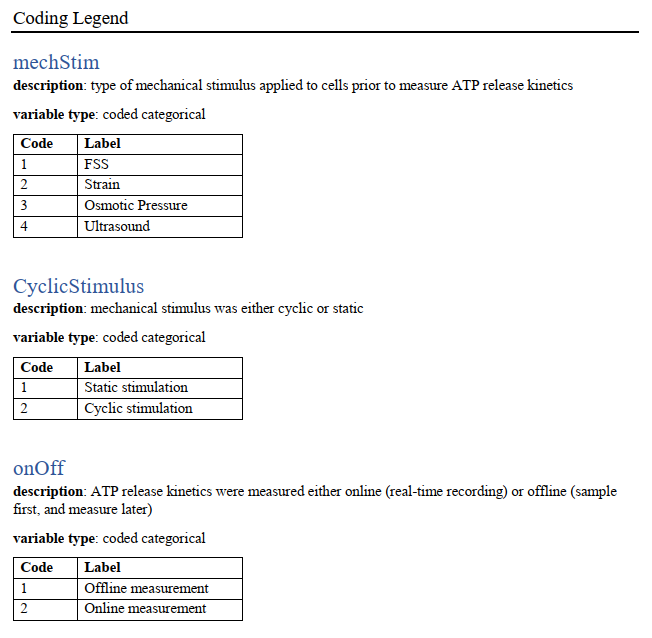


**Step-by-step instructions**

1. Save example.xlsx in same folder as MetaLab MATLAB files.
2. Open ‘MAIN.m’ in MATLAB
3. Press *Run* to initiate MetaLab
4. Select *Prepare Input* to open *Prepare Data Module*
5. Specify input and output file names in *Prepare Data Module*
   - Input data: name of excel file (e.g., example.xlsx) and sheet (e.g., Sheet1) in which input data are stored
   - Output data: name of Matlab data structure (e.g., example.mat) in which data will be stored. Ensure ‘save imported dataset’ option is selected.


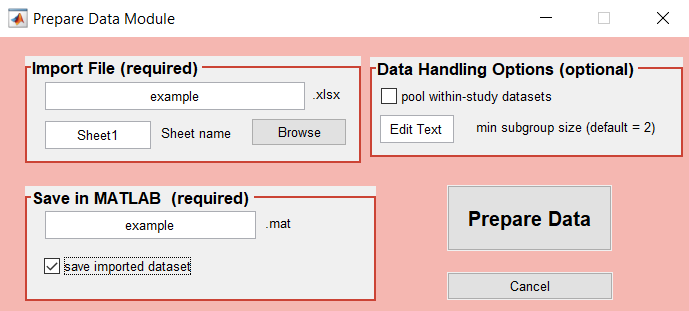


1. Press *Prepare Data* to being data preparation. *Subgroup Stratification* menu will open.


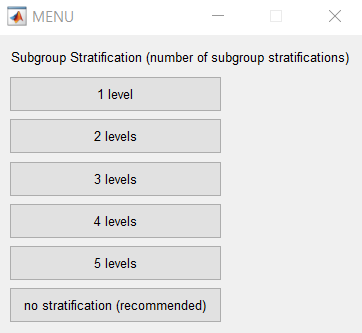


1. Select *no stratification* option. This will import excel sheet to MATLAB as one complete dataset labelled ‘totalSet’.
   - *Data preparation successful* prompt will appear. This indicates successful data import.


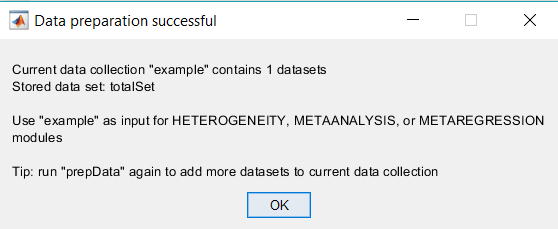


1. (Optional) To stratify data into subgroups prior to analysis, repeat steps 2-6 using same input (example.xlsx) and output (example.mat) datafiles. In the *Subgroup stratification* menu, select *1 level* of stratification to open *Covariate Selection* menu.
2. In the *Covariate Selection* menu, select which covariate (e.g., *onOff*) to stratify data by (ensure covariate is categorical variable).
   - Note. *onOff* covariate is a binary categorical variable that specifies which method was used to measure kinetics of ATP release (online measurement, *on=1*; offline measurement, *off=2*).


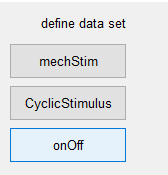


1. Data will be stratified into two subgroups and saved in ‘example.mat’ file. The ‘example.mat’ file is now ready for analysis.
   - ‘example.mat’ will have 3 datasets saved to it: ‘totalSet’, ‘onOff1’ and ‘onOff2’.
   - Note that all categorical covariates are coded prior to importing the data from excel. Users need to refer to the coding legend for subgroup labels for ‘onOff1’ and ‘onOff2’.


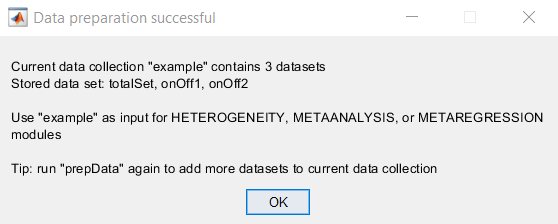


1. One data import and preparation are complete, press *Cancel* in *Prepare Data Module* to close module.


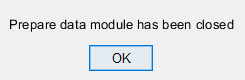


1. **To evaluate heterogeneity**, initiate MetaLab by pressing *Run* in ‘MAIN.m’ and selecting *Heterogeneity* in the *Module Selection* menu.
2. Import ‘example.xlsx’ and specify the analysis properties and options in the *Heterogeneity Module*
   - In our working example, the analysis properties were specified as shown below.
     - Note. the Log_10_ transformation method was selected because the data was determined to be skewed (i.e., lognormal).
   - The ‘Funnel plot’ and ‘Compare weighting schemes’ analysis options were selected.


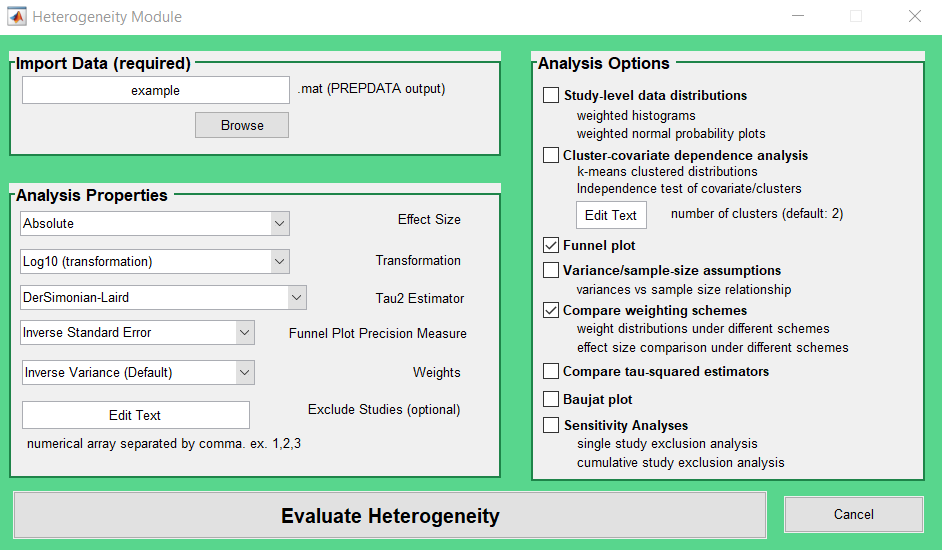


1. Press *Evaluate Heteroengeneity*
   - 3 sets of plots will be generated, one for each dataset saved in ‘example.mat’: ‘totalSet’, ‘onOff1’, ‘onOff2’.
   - The plots generated for the ‘totalSet’ using the selected analysis options are shown below. Additional options are detailed in the *MetaLab User guide*.

Funnel plot option:


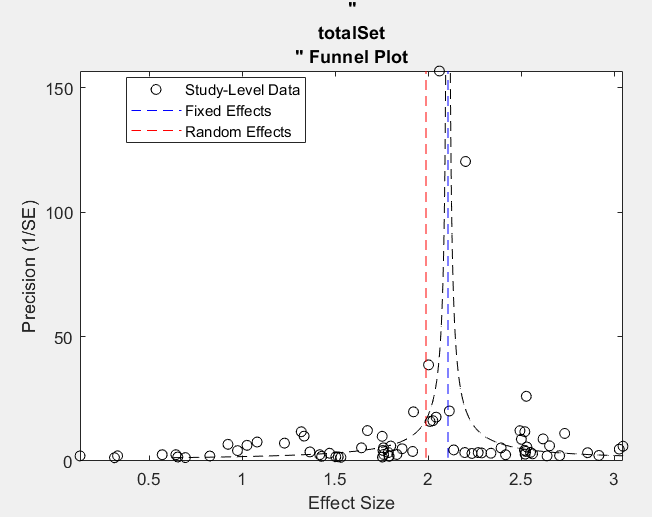


Compare weighting scheme option (compares unweighted [UW], fixed effects [FE], random effects [RE] and sample-size [N] weighting schemes):


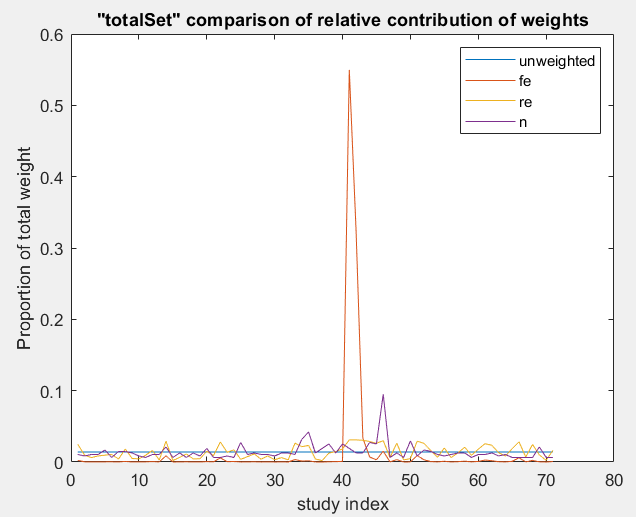

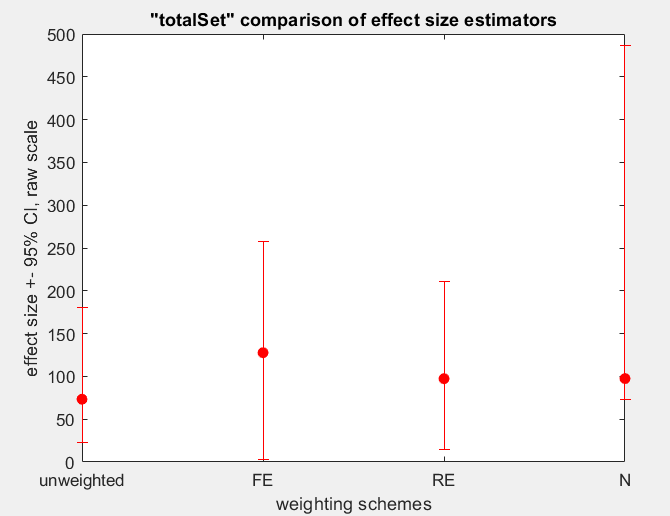


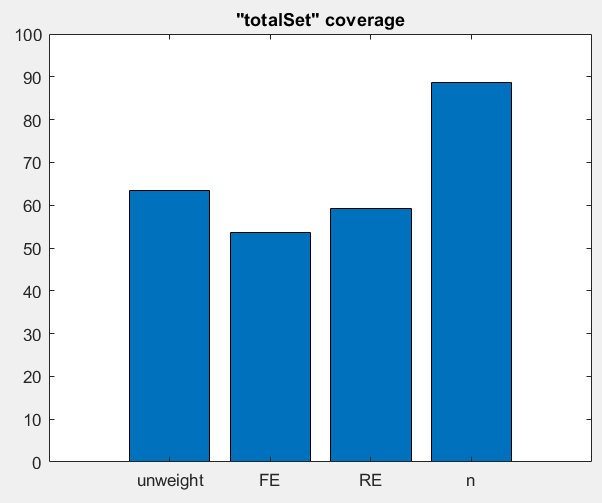


1. Save MetaLab generated figures and close *Heterogeneity Module* by pressing *Cancel*


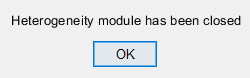


1. **For** **meta-analysis**, initiate MetaLab by pressing *Run* in ‘MAIN.m’ and selecting *Meta-Analysis* in the *Module Selection* menu.
2. Import ‘example.xlsx’ and specify the export file, analysis properties and forest plot properties in the *Meta-Analysis* *Module*
   - In our working example, the analysis properties were specified as shown below.
   - All the numerical results from the meta-analysis will be saved in the export file as an excel spreadsheet if the *export results* option is selected.


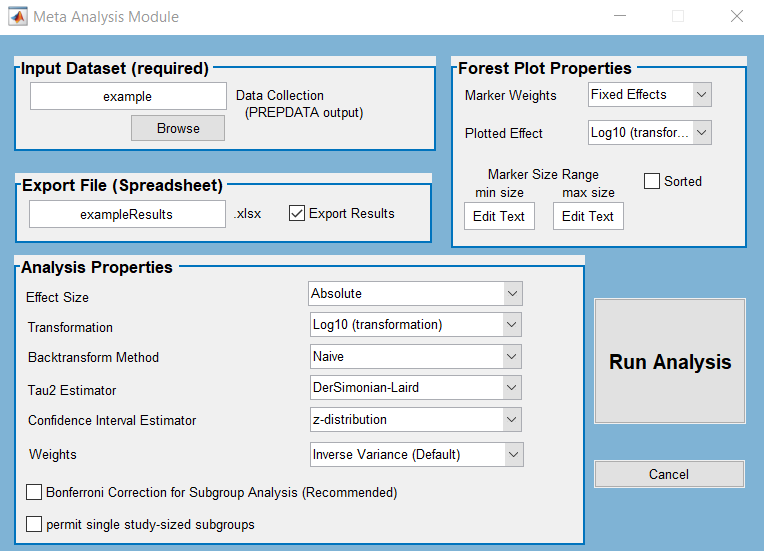


1. Press *Run Analysis* to conduct meta-analysis
   - For each dataset provided in the input dataset ‘example.mat’ (‘totalSet’, ‘onOff1’, ‘onOff2’), the following outputs will be generated:
     - ***Graphical output***: histogram and forest plot
     - ***Numerical output***: meta-analytic results and input data will be saved to export file
   - The plots generated for the ‘totalSet’ are shown below. Similar plots are generated for the ‘onOff1’ and ‘onOff2’ subgroups of data.


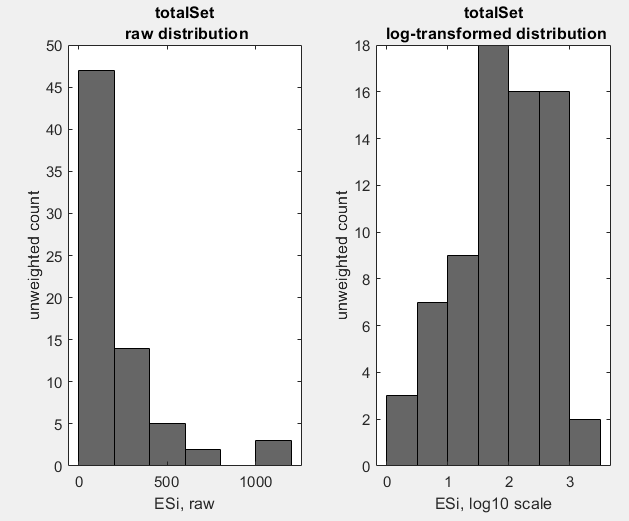

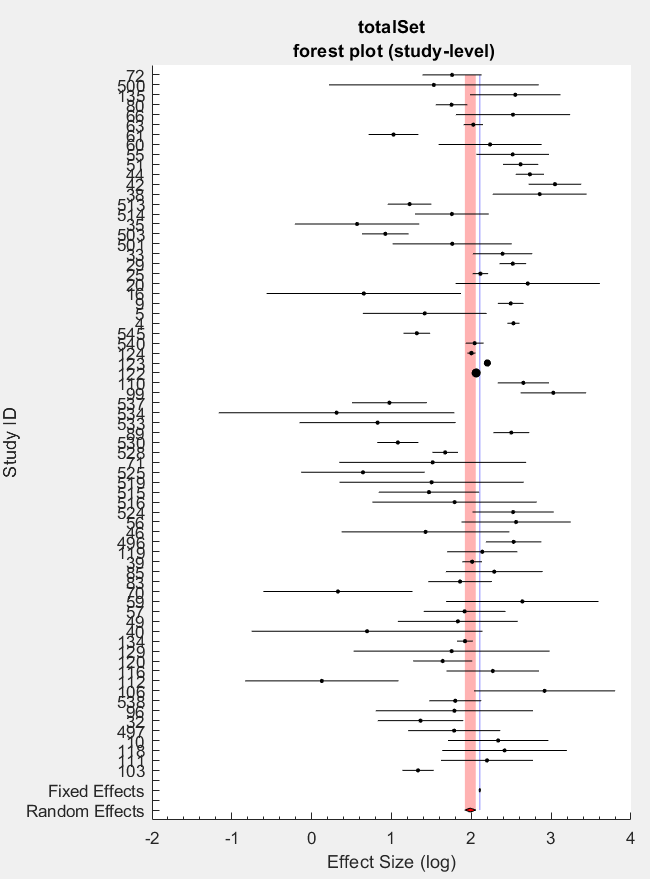


- - ‘exampleResults.xlsx’ will be saved to the same folder as the input data ‘example.mat’.
    - For each dataset provided in the ‘example.mat’ file, the ‘exampleResults.xlsx’ file will contain a corresponding *input data* sheet (e.g., ‘inputData_totalSet’) and *results* sheet (e.g., ‘totalSet’)

**Input Data (‘inputData_totalSet’) Sheet**


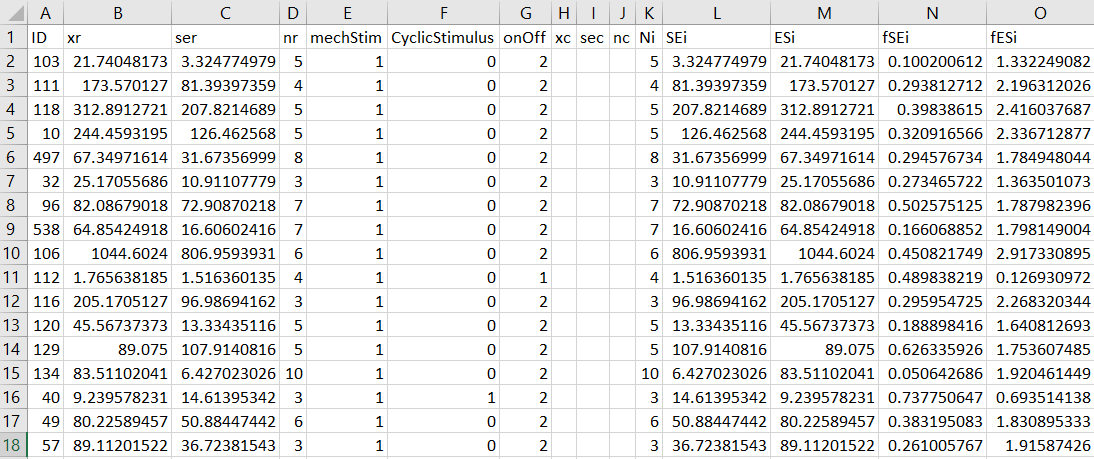


**ID**: study-index (required input), **xr**: case effect (required input), **ser**: case standard error (required input), **nr**: case sample size (required input), **mechStim**: categorical covariate (optional input), **CyclicStimulus**: categorical covariate (optional input), **onOff**: categorical covariate (option input, used in analysis), **xc**: control effect (optional input, n/a for example dataset), **sec**: control standard error effect (optional input, n/a for example dataset), **nc**: control sample size effect (optional input, n/a for example dataset), **Ni**: study-level sample size, **SEi**: study-level standard error (raw scale), **ESi**: study-level effect size (raw scale), **fSEi**: study-level standard error (log10 scale), **fESi**: study-level effect size (log10 scale; fSEi and fESi are on log10 scale because log10 transformation option was selected, overwise would be on raw scale).

**Results (‘totalSet’) Sheet**

**
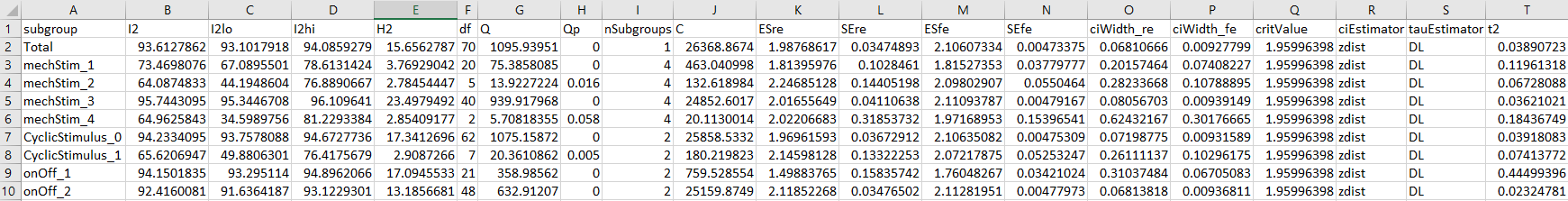
**

**Subgroup**: subset of data analysis (refer to coding legend for labels), **I2**: I^2^ heterogeneity statistics, **I2lo**: lower bound confidence interval for I2, **I2hi**: upper bound confidence interval of I2, **H2**: H^2^ heterogeneity statistics, **df**: degrees of freedom (number of datasets N = df+1), **Q**: Q heterogeneity statistic, **Qp**: p-value for Q-test, **nSubgroups**: number of subgroups, C**:** scaling parameter for heterogeneity statistics, **ESre**: random effects effect size, **SEre**: random effects standard error, **ESfe**: fixed effect size, **SEfe**: fixed effect standard error, **ciWidth**_re: random effects confidence interval width, **ciWidth**_fe: fixed effect confidence interval, **critValue**: critical value used to compute confidence interval width, **ciEstimate**: distribution use to estimate confidence interval width, **tauEstimator**: tau^2^ estimator to estimate between-study variance, **tau2**: tau^2^ heterogeneity statistic.

1. Interpret results
   - Results provided in ‘exampleResults.xlsx’ can now be interpreted and reported as needed.

The MetaLab User Guide also provided details on additional analysis options which are not covered in this working example.
